# Atypical cytokine profiles in people on the autism spectrum: a comprehensive systematic review and meta-analysis including 54 cytokines

**DOI:** 10.1038/s41380-026-03659-4

**Published:** 2026-05-28

**Authors:** Theresia Volk, Steve Lukito, Joaquim Radua, Hella Luksch, Veit Roessner, Nicole Beyer

**Affiliations:** 1 Department of Child and Adolescent Psychiatry and Psychotherapy, Medical Faculty Carl Gustav Carus at the TU Dresden, Dresden, Germany; 2 Department of Child & Adolescent Psychiatry, Institute of Psychiatry, Psychology and Neuroscience, King’s College London, London, UK; 3 Institut d’Investigacions Biomèdiques August Pi I Sunyer; Department of Medicine, University of Barcelona, Institute of Neuroscience, Barcelona, Spain; 4 Centro de Investigación Biomédica en Red de Salud Mental (CIBERSAM), Instituto de Salud Carlos III, Madrid, Spain; 5 Department of Paediatrics and the Institute for Immunology, Medical Faculty Carl Gustav Carus at the TU Dresden, Dresden, Germany; 6 German Centre for Child and Adolescent Health (DZKJ), partner site Leipzig/Dresden, Dresden, Germany

**Keywords:** Diagnostic markers, Psychology

## Abstract

Atypical peripheral blood cytokine concentrations have been shown in autism, but no clear pattern has been observed. This systematic review and meta-analysis summarised current state of findings, expanded the range of cytokines, accounted for study risk of bias, and examined relations between cytokines and autism traits. Literature comparing peripheral blood cytokine in autistic and non-autistic people was systematically searched in Ovid^®^ Embase, MEDLINE and APA PsycINFO, Web of Science^™^ and Scopus, resulting in 98 studies and 54 cytokines (4236 autistic, 3333 non-autistic controls; age 2 to 65 years) in the meta-analysis. Study risk of bias was assessed using adapted Newcastle-Ottawa Scale. Compared to controls, autistic people had elevated levels of IL1-beta (Hedges’ *g* = 0.620, 95%CI[0.32, 0.92]), IL4 (*g* = 0.245, 95%CI [0.07, 0.42]), IL6 (*g* = 0.365, 95%CI [0.011, 0.62]), IL8 (*g* = 0.384, 95%CI [0.15, 0.62]), IFN-gamma (*g* = 0.404, 95%CI [0.09, 0.72]), TNF-alpha (*g* = 0.31, 95%CI [0.11, 0.51]), CXCL1/GRO-α (*g* = 0.364, 95%CI [0.058, 0.670]) and MIF (*g* = 0.560, 95%CI [0.14, 0.98]). Over a third of studies were classified as having a high risk of bias; their removal revealed higher IL7 and IL1RA in autism relative to controls. Narrative synthesis produced no strong evidence for an association between cytokine and autism traits among autistic individuals. Altogether, our findings support a predominance of pro-inflammatory cytokines, while also indicating potential modulatory contributions from inhibitory cytokines, which reflect group-level differences between autistic and non-autistic individuals, but not variations of autism traits within the autistic population. However, higher-quality studies with low risk of bias are needed before firm conclusions can be drawn.

## Introduction

Autism spectrum disorder (ASD) is a psychiatric diagnosis for a childhood-onset condition [1]. People with a diagnosis of ASD, referred to here as ‘autistic people’ or people ‘on the autism spectrum’ [2, 3], are characterised by difficulties in social communication and restricted, repetitive behaviour [1]. Autism is highly genetic, but the influence of environmental factors and their interaction with the genetics of the condition has been increasingly recognised [4, 5]. Various environmental prenatal factors associated with inflammatory processes, including maternal autoimmune diseases, infections, and stress during pregnancy, have been linked to autism [6–10]. An increasing number of studies have shown atypical cytokine profiles in autistic people compared to non-autistic controls in cerebrospinal fluid, brain tissue and peripheral blood [11–13], strengthening the evidence for the atypically regulated inflammatory processes in the condition.

Cytokines are pleiotropic messenger proteins consisting of different subgroups including chemokines, interferons, and growth factors, that moderate the activity of the immune system [14]. Together, they play a crucial role in the shaping and functioning of the central nervous system [14, 15]. Firstly, there are networks in the brain composed of neurons and glial cells, which can both secrete cytokines and be activated by them. These networks can therefore be referred to as ‘cytokine networks’ [15, 16]. Secondly, these cytokine networks are also affected by peripheral cytokines as they can signal to the brain via cellular, neural, and humoral pathways [17]. Since cytokines influence brain development and homeostasis, dysregulation of cytokine networks—usually detected in forms of atypical levels of peripheral cytokines—may therefore be associated with pathophysiological effects on neurodevelopment and related behaviours [14, 15].

In line with that, various cohort studies have reported associations between atypically regulated neonatal cytokine profiles and an increased probability of being given the diagnosis of ASD later in life [18, 19]. Higher concentrations of cytokines have also been found to be associated with increased autistic symptom scores in autism in some studies [20, 21]. As has been shown in previous meta-analyses, altered cytokine concentration in peripheral blood could also be observed in older autistic children and adults compared to non-autistic controls [22–25]. By understanding the specific cytokine profile in autism, we could perhaps elucidate what kind of anti-inflammatory and immunomodulatory interventions could benefit the condition [26, 27].

To date, no coherent pattern of atypical cytokine profiles in peripheral blood has been identified among autistic people, although previous findings suggested the predominance of pro-inflammatory cytokines in the condition [22, 24]. Since the last meta-analyses on this topic [22–25] many studies examining cytokine concentrations in the peripheral blood of autistic people have been published [12, 28–34]. Furthermore, recent meta-analyses tend to lack assessment of individual study risk of bias as well as the consideration of potential moderators such as autism symptom scores [23–25]. Therefore, we update previous findings by conducting a current meta-analysis of all published studies comparing the cytokine profiles (i.e., cytokine concentration in peripheral blood serum or plasma) in autistic people with non-autistic controls while considering the influence of risk of bias across studies and autism symptom scores, and potential moderators including age, sex, control participant type, medication status, and peripheral blood sample type.

## Methods

### Search strategy

Our systematic literature search strategy combined key terms related to autism (i.e., autis* *or* ASD *or* “pervasive development* disorder*” *or* Asperger*) *and* cytokines (e.g., cytokin* *or* interleukin* *or* chemokin*; see the full list in Supplementary Tables S1-3). The literature search used the databases Embase, MEDLINE, and APA PsycINFO on the Ovid^®^ platform, Web of Science^®^, and Scopus^®^. The first wave of search was carried out from the databases’ inception to 9th June 2021, a second search was conducted for published studies from 1^st^ January 2021 to 30th December 2022, and a third wave of literature search was conducted from 1^st^ January 2023 to 17^th^ March 2025. These were supplemented by a manual reference search using past meta-analyses [22–25]. The systematic review and meta-analysis protocol was pre-registered in PROSPERO (CRD42022304200). We observed the Preferred Reporting Items for Systematic Reviews and Meta-Analysis (PRISMA2020) guidelines (Table S4) [35].

### Eligibility criteria

Studies were included if they (a) contained comparisons of concentrations of peripheral blood cytokines (i.e., in blood plasma or serum) between autistic people and non-autistic controls; (b) were peer-reviewed publications; (c) were written the English language. We excluded (c) studies involving groups of autistic people with neurological conditions (such as seizures) or genetic syndromes, gastrointestinal and autoimmune disorders, or those with acute infections. We also excluded (d) studies containing secondary data analyses such as systematic reviews and meta-analyses, (e) studies that only analysed cytokine concentrations in neonatal blood, peripheral blood mononuclear cells (PBMC), monocytes, leukocytes, lymphocytes, or after mitogen stimulation, and studies examining gene expression profiles. Studies involving participants who were taking medication were not excluded; however, the variable was included in the analyses as a moderator. When studies with overlapping samples were encountered, the authors of these studies were contacted to assist in selecting the studies to include in the meta-analysis.

### Study selection

Identified studies underwent semi-automated removal of duplicates, assisted by the software EndNote20^®^ (Philadelphia, PA). The screening of studies was conducted in two stages, independently by two reviewers (TV, SL), using the online tool Rayyan (https://www.rayyan.ai/). The screening was conducted first by title and abstract, and second, by reading the full text of these studies. To achieve inter-rater reliability, screening was piloted on batches of ~10% of the initially identified studies (n = 490) until an agreement rate of Cohen’s Kappa (ĸ) = 0.80 was reached. The overall screening agreement rate was ĸ = 0.87 (i.e., 99.2%), indicating a “strong agreement” [36]. Screening discrepancies were resolved through consensus, involving a third researcher (NB) if necessary.

### Data extraction

The extracted data included (a) information about the study, including authors’ names and publication year, (b) sample sizes and characteristics, including measures of central tendency/spread of age, sex (i.e., percent of males), autism symptoms indexed by validated measures, diagnosis and ascertainment, and where available, IQ measures and medication status; (c) information about cytokine concentration measurement, including number and types of cytokine analysed, and measures of central tendency/spread of the cytokine concentration, type of cytokines, measures of an effect size of the group differences where available, *p*-values, and the peripheral blood type (i.e., serum or plasma). If necessary, further information and unpublished data were requested from the authors. Data were extracted independently by TV and SL, and discrepancies were discussed to reach a consensus, if necessary, involving the third researcher, NB.

### Risk of bias assessment

The risk of bias in each study was evaluated by two independent reviewers (TV, NB) using the Newcastle-Ottawa scale adapted for cross-sectional studies [37]. The risk of bias was judged as low (7 to 8 points), medium (5 to 6 points), or high (1 to 4 points), according to prespecified criteria based on sample selection, comparability of case and control participants, and the assessments of cytokine concentration (see Tables S5, S6).

### Statistical analysis plan

Extracted data were visually inspected and, where possible, we estimated missing means and standard deviations from the reported sample sizes, medians, ranges, and/or interquartile ranges using established formulas [38]. Cytokine concentration differences between the autistic and the non-autistic groups were synthesised using quantitative meta-analyses when there were at least three unique datasets, following previous approaches [22–25].

The meta-analyses were conducted on each cytokine type, organised by cytokine families (i.e., interleukin, interferons, tumour necrosis factors, colony-stimulating factors, chemokines, growth factors, adipokines, and other cytokines), using a random effect model with the *R* package *Metafor* [39]. Effect sizes (Hedge’s *g*) were calculated from means, standard deviations (alternatively, *p*-values), and sample sizes. When studies reported median, and lower and upper quartiles instead, mean and standard deviation were first estimated following the default approach in the *R* package. The *p*-value of 0.05 was used as a threshold of significance for assessing differences between autistic and non-autistic groups, corrected for false discovery rate (FDR) using Benjamini-Hochberg’s method in each cytokine family. Forest plots were used to visualise observed effects, 95% confidence interval (CI), and the weight of individual studies, as well as the overall effect. Heterogeneity across studies was quantified in Cochran’s *Q*, with *p *= 0.10 due to the low power of the test. The *I*^2^ of 0.25,0.50, and 0.75 were used to index small, moderate, and large levels of heterogeneity.

Cytokines found to be significantly different in concentration (at *p* < 0.05 after FDR correction) between the autistic and non-autistic groups were submitted to follow-up multiple meta-regression analyses. Age, sex, control participant type, medication status, peripheral blood sample type, as well as DSM diagnostic criteria were explored as moderators of the heterogeneous findings in cytokine concentration differences. This was conducted separately in the first instance, then with all moderators combined using the available reported data. Then this was repeated by applying the multiple imputation by chained equations (MICE) [40] method to account for missing data.

Where applicable, analyses were repeated excluding studies judged to have a high risk of bias on the adapted Newcastle Ottawa Scale [37]. Publication bias was assessed using Egger’s test of the Funnel plot’s asymmetry [41].

Finally, our data extraction identified several studies simply reporting a non-significant difference between groups, without providing any statistics (these are termed nonsignificant unreported effects [NSUE] [42]). Traditionally, such studies would be excluded from a meta-analysis; consequently, this would inflate the estimation of effects. Therefore, we conducted additional analyses to adjust effect size estimates in those cytokines for completion using the R package *MetaNSUE* [42].

### Narrative synthesis

Relations between cytokine concentration and autism traits were explored using narrative synthesis as a secondary examination. This approach was employed since there was an insufficient number of studies using homogeneous measures for meta-analysis. Syntheses were undertaken using findings from studies examining the association between cytokine concentrations and autism traits using validated measures, including Childhood Autism Rating Scale (CARS) [43], Autism Diagnostic Observation Schedule (ADOS) [44], Autism Diagnostic Interview-Revised (ADI-R) [45], Social Communication Questionnaire (SCQ) [46], Social Responsiveness Scale (SRS) [47], and Aberrant Behaviour Checklist [48] among others. The association between cytokine levels and traits could be investigated in group comparisons of autistic individuals with different levels of traits, usually defined by the cutoff scores of standardised measures [43, 44], or using correlational investigations involving dimensional traits measured in autistic individuals only. We also focused on examining the associations between cytokines and autism traits in cytokines that differed in concentration between the autistic and the non-autistic groups.

### Examination of level of evidence

The level of evidence was examined using conventional criteria [49], judged based on the meta-analytic estimation of effects, number of cases, and heterogeneity level of findings, among others. Under these criteria, convincing evidence (class I) status was assigned to evidence obtained from over 1000 cases, with estimate of effect reaching a *p*-value < 10^−6^, lower finding heterogeneity than *I*^2^ < 50%, where 95% prediction interval excluded the null, among others, highly suggestive evidence (class II) status was assigned to evidence obtained from over 1000 cases, with an estimate of effect reaching a *p*-value < 10^−6^, where other criteria for class I criteria were not met; weak evidence (class IV) was assigned to evidence obtained with cases lower than 1000, which reached the threshold of *p*-value < 0.05 and did not met the criteria for class I–III, and lastly non-significant evidence was when the estimated effect had a *p*-value > 0.05 [49].

## Results

### Study selection

**Fig. 1:**
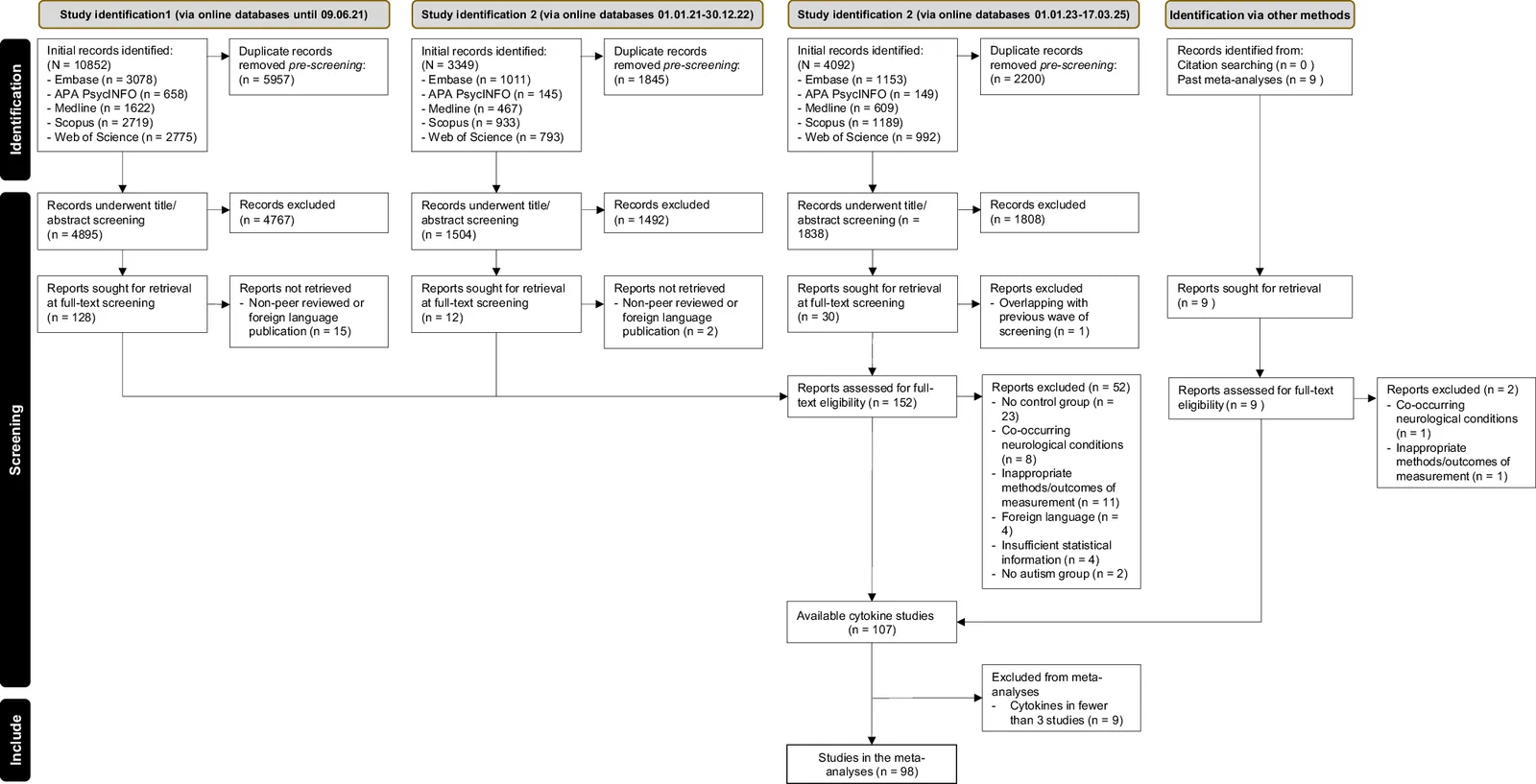
PRISMA Flow Diagram of Study Selection. Study identification via online databases was conducted in two stages, supplemented by a manual search of references through past meta-analyses. Screening stages were completed independently by two reviewers (TV, SL). Excluded studies at full-text screening stages are listed in Supplementary Table S7.

We retrieved 10,852 studies from the first, 3349 from the second, and 4092 from the third wave of systematic literature search (Fig. 1). Nine further studies were identified from past meta-analyses. After the removal of duplicates, the completion of title and abstract screening, and the exclusion of reports that could not be retrieved, 161 studies were assessed for full-text eligibility criteria (152 from the current systematic search, and nine from past meta-analyses), of which 54 were excluded mainly due to the absence of a control group, the co-occurrence of neurological conditions, or inappropriate methods/outcomes of measurement (see Table S7 for the full list of excluded studies and exclusion reasons). This left 107 unique cytokine studies available for meta-analyses, from which nine were further excluded as they only reported cytokines that were investigated overall in fewer than three datasets. Therefore, the meta-analysis included 98 studies, with at least three datasets per cytokine to meta-analyse (Table S8).

### Characteristics of studies in the meta-analysis

Overall, our meta-analyses involved 4236 autistic people and 3333 non-autistic controls (Table S8). Across the studies, 92 studies included autistic children only, five included autistic adults only, and one small study (n = 12) included a mix of child and adult participants. The age of participants ranged from 2 to 65 years, with the majority being children (97%, computed from studies in children or adults exclusively). Sex was reported in 87 studies (82.6% autistic and 74.5% non-autistic males). Half of the studies reported cytokine levels in serum, and the remaining half in plasma. Over half of the studies (n = 52; 53%) reported findings in non-medicated participants, 9 (7%) reported findings in participants who took medication, while 39 (40%) did not report medication. Autistic participants in most studies (n = 85 [86.7%]) met the criteria for autism diagnosis according to the Diagnostic Statistical Manual of Mental Disorders, 4^th^ edition, its text revision, or the 5^th^ edition (DSM-IV, DSM-IV-TR, and DSM-5). The meta-analysis for each cytokine consisted of 3 to 44 studies. Altogether, the meta-analyses investigated 54 cytokines.

Sixty-one studies investigated associations between cytokine concentrations and autism traits with validated measures (45 correlation; 13 trait subgroup comparisons, and 3 both approaches). Studies that examined this using subgroup comparisons typically compared those having “severe” with those having “mild-to-moderate” traits as defined by the cutoffs of measures such as the ADOS or the CARS.

### Risk of bias assessment

**Fig. 2:**
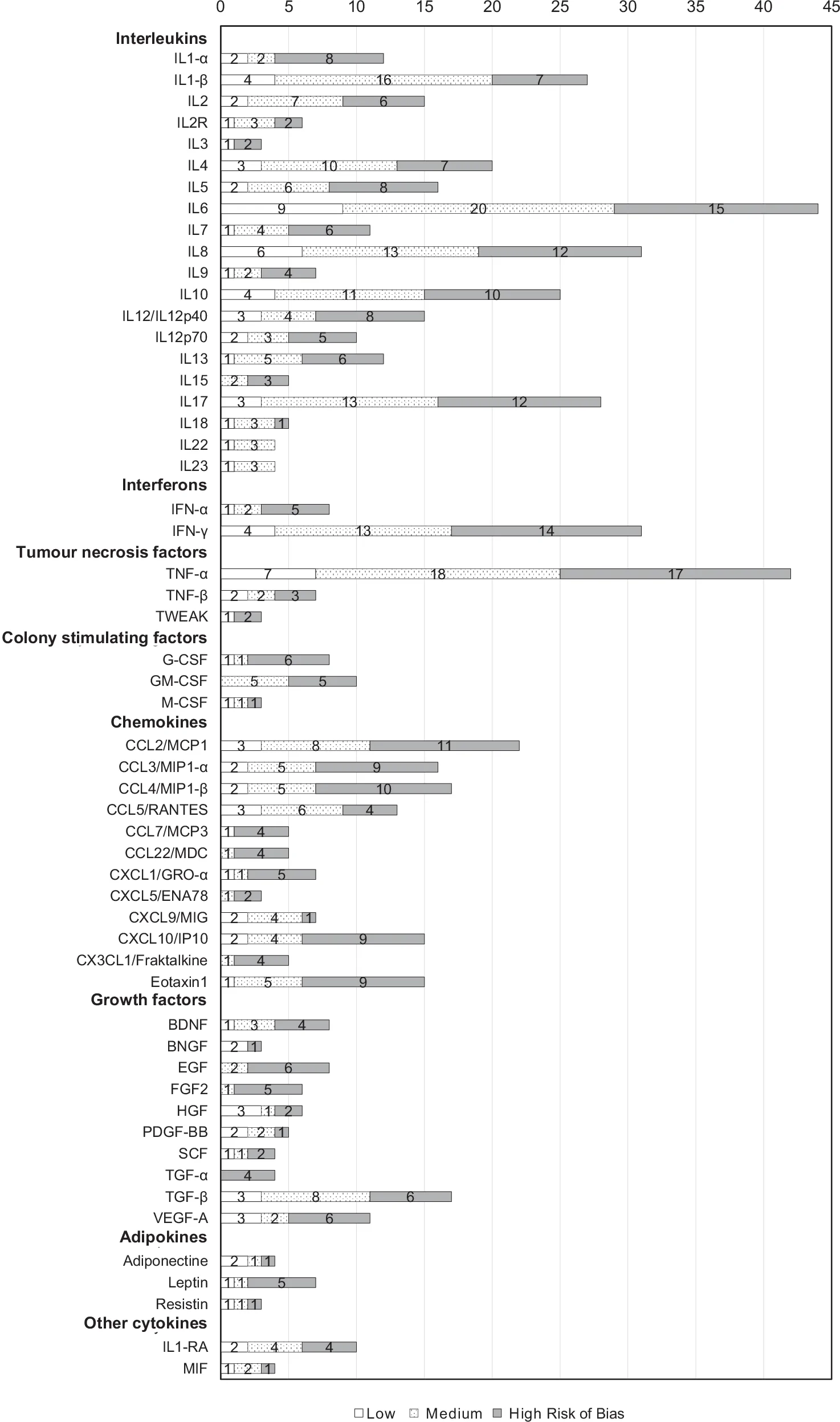
Risk of Bias Across Studies per Cytokine. The number of studies per cytokines with low, medium, and high risk of bias. IL interleukin, IFN interferon, TNF tumour necrosis factor, TWEAK (TNF)-like weak inducer of apoptosis, G-/GM-/M-CSF granulocyte-/granulocyte macrophage-/macrophage-colony stimulating factor, CCL chemokine ligand, CXCL C-X-C motif ligand, MCP monocyte chemoattractant protein, MIP macrophage inflammatory protein, RANTES regulated upon activation, normal T cell expressed and presumably secreted, MDC macrophage-derived chemokine, GRO growth-regulated oncogene, ENA78 epithelial neutrophil-activating protein 78, MIG monokine induced by gamma interferon, IP10 interferon γ-induced protein 10, BDNF brain-derived neurotrophic factor, BN/E/F/H/PD/T/VEGF beta-nerve/epidermal/fibroglast/hepatocyte/platelet-derived/transforming/vascular endothelial growth factor, SCF stem cell factor, RA receptor agonist, MIF macrophage migration inhibitory factor.

Out of 98 studies, 33 (33.7%) had a high, 41 (41.8%) a moderate, and 24 (24.5%) a low risk of bias, respectively (Table S6). Studies with high risk of bias were present in at least half in 30 of the 55 individually completed cytokine meta-analyses, including those examining IL1-alpha, IL3, IL5, IL7, IL9, IL12/IL12p40, IL12p70, IL13, IL15, IFN-alpha, TWEAK, G-CSF, GM-CSF, CCL2/MCP1, CCL3/MIP1-alpha, CCL4/MIP1-beta, CCL7/MCP3, CCL22/MDC, CXCL1/GRO-alpha, CXCL5/ENA78, CXCL10/IP10, CX3CL1/Fractalkine, Eotaxin1, BDNF, EGF, FGF-2, SCF, TGF-alpha, VEGF-A, and Leptin (Fig. 2). The top three factors contributing to poorer risk-of-bias ratings were the inadequate reporting of co-occurring conditions and medication intake, which may bias the cytokine concentration findings, and clarity of participant eligibility criteria (Table S6).

### Meta-analytic results

**Fig. 3:**
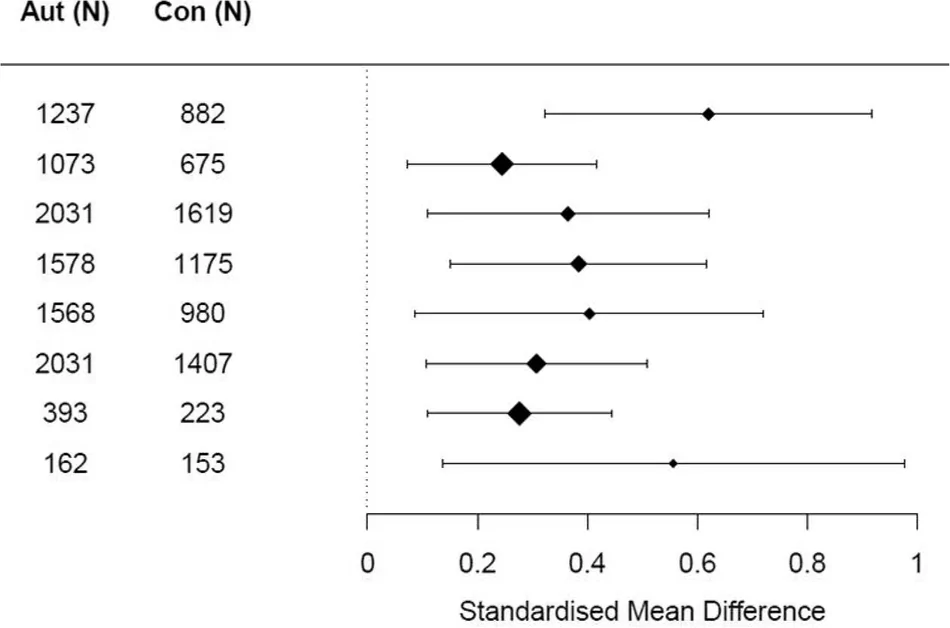
Summary Forest Plots of Significant Group Differences. Summary forest plot depicting cytokines that exhibit significant concentration differences, after adjusting for false-discovery rates, between the autistic (Aut) and non-autistic (Con) groups. Concentration differences were estimated using all available data, accompanied by the sample sizes (N) and 95% confidence intervals. IL interleukin, IFN interferon, TNF Tumour Necrosis Factor, CXCL C-X-C motif ligand, GRO growth-regulated oncogene, and MIF macrophage migration inhibitory factor.

Table 1 lists the main meta-analytic findings, of which significant summary statistics are depicted in the summary forest plot in Fig. 3 (See Figures S1, S2 for detailed forest plots consisting of the individual studies).

**Table 1:** Meta-analytic Findings Using All Available Data.

Cytokines	Datasets	Autism (n)	Controls (n)	Hedge’s *g*	95%CI	*p*	Cochran’s Q	*p*	*I*^2^ (%)
**Interleukins**									
IL1-alpha	12	638	342	0.061	[−0.19, 0.31]	0.628	37.2	0.0001***^, ‡^	67.6
**IL1-beta**	**28**	**1237**	**882**	**0.620**	**[0.32, 0.92]**	<0.0001***^, ‡^	**243.7**	<0.0001***^, ‡^	**89.6**
IL2	15	789	544	0.137	[−0.05, 0.32]	0.151	34.1	0.002**^, ‡^	60.9
IL3	3	169	113	−0.286	[−0.59, 0.02]	0.066	3.17	0.204	32.5
**IL4**	**20**	**1073**	**675**	**0.245**	**[0.07, 0.42]**	0.005*^, ‡^	**48.2**	0.0001***^, ‡^	**62.6**
IL5	16	930	588	0.164	[−0.004, 0.33]	0.055	34.5	0.003**^, ‡^	57.9
**IL6**	**44**	**2031**	**1619**	**0.365**	**[0.11, 0.62]**	0.005*^, ‡^	**320.3**	<0.0001***^, ‡^	**92.4**
IL7	12	609	371	0.039	[−0.20, 0.28]	0.745	27.8	0.002**^, ‡^	64.9
**IL8**	**31**	**1578**	**1175**	**0.384**	**[0.15, 0.62]**	0.001*^, ‡^	**194.8**	<0.0001***^, ‡^	**87.6**
IL9	6	305	182	−0.031	[−0.22, 0.16]	0.745	4.657	0.459	0
IL10	25	1279	763	−0.252	[−0.82, 0.31]	0.381	387.1	<0.0001***^, ‡^	97
IL12/IL12p40	15	741	498	0.478	[−0.01, 0.96]	0.054	138.2	<0.0001***^, ‡^	93.6
IL12p70	10	541	366	0.274	[−0.24, 0.79]	0.298	114.6	<0.0001***^, ‡^	92.5
IL13	12	745	437	0.047	[−0.23, 0.32]	0.737	43.5	<0.0001***^, ‡^	78.6
IL15	5	286	179	−0.046	[−0.28, 0.18]	0.693	5.34	0.253	25.4
IL17	28	1490	928	1.021	[−0.001, 2.04]	0.050	371.8	<0.0001***^, ‡^	99.2
IL18	5	238	169	0.340	[0.05, 0.63]	0.023	7.72	0.103	98.8
IL22	4	201	153	1.057	[−1.18, 3.30]	0.355	79.7	<0.0001***^, ‡^	98.8
IL23	4	240	180	−0.220	[−0.71, 0.27]	0.379	19.9	<0.001***^, ‡^	82.9
**Interferons**									
IFN-alpha	8	482	281	0.049	[−0.12, 0.21]	0.563	9.65	0.209	16.1
**IFN-gamma**	**31**	**1568**	**980**	**0.404**	**[0.09, 0.72]**	0.013*^, ‡^	**183.9**	<0.0001***^, ‡^	**92.6**
**Tumour necrosis factors**									
**TNF-alpha**	**42**	**2031**	**1407**	**0.307**	**[0.11, 0.51]**	0.003**^, ‡^	**253.1**	<0.0001***^, ‡^	**86.8**
TNF-beta	7	399	267	0.150	[−0.26, 0.56]	0.467	37	<0.0001***^, ‡^	84.3
TWEAK	4	165	108	0.494	[−0.10, 1.09]	0.106	10.6	0.005**^, ‡^	82.1
**Colony stimulating factors**									
G-CSF	8	374	216	0.045	[−0.34, 0.43]	0.820	32.12	<0.0001***^, ‡^	78.7
GM-CSF	10	581	364	0.129	[−0.08, 0.34]	0.224	20	0.018*^, ‡^	54.8
M-CSF	3	113	81	0.074	[−0.22, 0.37]	0.619	2	0.369	0
**Chemokines**									
CCL2/MCP1	22	1036	733	1.441	[−0.29, 3.17]	0.103	339.8	<0.0001***^, ‡^	99.6
CCL3/MIP1-alpha	16	861	612	−1.001	[−3.22, 1.22]	0.376	288.6	<0.0001***^, ‡^	99.7
CCL4/MIP1-beta	17	905	633	−0.014	[−0.74, 0.71]	0.971	307.9	<0.0001***^, ‡^	97.7
CCL5/RANTES	13	584	443	0.278	[−0.008, 0.56]	0.057	53.8	<0.0001***^, ‡^	78.7
CCL7/MCP3	5	314	156	−0.068	[−0.26, 0.13]	0.494	3.75	0.441	0
CCL11/Eotaxin-1	15	737	524	0.238	[0.001, 0.47]	0.049	66.3	<0.0001***^, ‡^	74.4
CCL22/MDC	5	258	155	0.227	[−0.18, 0.63]	0.270	15.4	0.004**^, ‡^	73.9
**CXCL1/GRO-alpha**	**7**	**393**	**223**	**0.277**	**[0.11, 0.44]**	0.001*^, ‡^	**6.45**	**0.375**	**0.01**
CXCL5/ENA78	3	143	105	0.352	[0.01, 0.69]	0.041	2.96	0.227	35.8
CXCL9/MIG	7	278	224	−0.261	[−0.73, 0.21]	0.276	30.1	<0.0001***^, ‡^	84.4
CXCL10/IP10	15	756	483	−0.471	[−1.01, 0.06]	0.084	185.2	<0.0001***^, ‡^	94.26
CX3CL1/Fractalkine	5	320	218	0.051	[−0.24, 0.34]	0.730	9.8	0.044	59.8
**Growth Factors**									
BDNF	8	339	211	0.166	[−0.18, 0.51]	0.349	21.2	0.004**^, ‡^	69.5
BNGF	3	109	72	0.245	[−0.06, 0.55]	0.114	1.44	0.488	0
EGF	8	544	282	−0.124	[−0.68, 0.44]	0.666	83.7	<0.0001***^, ‡^	92.5
FGF2	6	381	202	0.164	[−0.19, 0.52]	0.369	22	0.0005**^, ‡^	73.9
HGF	6	280	203	−0.137	[−0.75, 0.48]	0.662	35.4	<0.0001***^, ‡^	89.8
PDGF-BB	5	196	152	0.130	[−0.24, 0.50]	0.489	9.73	0.045	59.2
SCF	4	225	153	−0.234	[−0.52, 0.05]	0.110	5.37	0.146	43.4
TGF-alpha	4	282	144	0.147	[−0.05, 0.35]	0.154	0.46	0.928	0
TGF-beta	17	675	564	−0.120	[−0.62, 0.38]	0.640	159.2	< .0001***^, ‡^	94.1
VEGF-A	11	498	383	0.087	[−0.15, 0.33]	0.478	29.6	0.001**^, ‡^	64.2
**Adipokines**									
Adiponectin	4	132	103	−0.703	[−1.76, 0.35]	0.190	33	<0.0001***^, ‡^	93
Leptin	7	332	186	1.01	[−0.17, 2.19]	0.093	68.5	<0.0001***^, ‡^	97.2
Resistin	3	96	67	−1.59	[−5.89, 2.70]	0.466	95.1	<0.0001***^, ‡^	99.1
**Other cytokines**									
IL1-RA	10	506	347	0.152	[−0.11, 0.41]	0.246	24	0.004**^, ‡^	64.8
**MIF**	**4**	**162**	**153**	**0.560**	**[0.14, 0.98]**	0.009*^, ‡^	**7.35**	**0.062**	**56.7**

The summary group differences in cytokine peripheral blood concentration between autistic people and non-autistic controls in the meta-analyses using all available data, reported alongside 95% confidence interval (CI), and finding heterogeneity indices (Cochran *Q* and *I* [2]). Bold-printed rows indicate statistically significant group differences, after adjusting for false discovery rate (FDR).

*IL* interleukin, *IFN* interferon, *TNF* tumour necrosis factor, *G-/GM-CSF* granulocyte-/granulocyte-macrophage colony stimulating factor, *CCL* chemokine ligand, *CXCL* C-X-C motif ligand, *MCP* monocyte chemoattractant protein, *MIP* macrophage inflammatory protein, *NA* not applicable, *RANTES* regulated upon activation, normal T cell expressed and presumably secreted, *MDC* macrophage-derived chemokine, *GRO* growth-regulated oncogene, *MIG* monokine induced by gamma interferon, *IP10* interferon gamma-induced protein 10, *BDNF* brain-derived neurotrophic factor, *E/F/H/PD/T/VEGF* epidermal/fibroglast/ hepatocyte/platelet-derived/transforming/vascular endothelial growth factor, *RA* receptor agonist, *MIF* macrophage migration inhibitory factor.

^**‡**^Original *p*-values are reported in the Table, but the asterisks indicated significant thresholds after FDR-correction, * *p*_FDR_ <.05, ** *p*_FDR_ <.01 and *** *p*_FDR_ < 0.001. IL12p70 was analysed separately from other IL12/IL12p40 since it is the bioactive form of IL12.

#### Interleukins (IL)

Among the interleukins (Table 1), IL1-beta (Hedges’ *g* = 0.620, 95% CI [0.32, 0.92], *p* < 0.0001), IL4 (*g* = 0.245, 95% CI [0.07, 0.42], *p* = 0.005), IL6 (*g* = 0.365, 95% CI [0.011, 0.62], *p* = 0.005) and IL8 (*g* = 0.384, 95% CI [0.15, 0.62], *p* = 0.001) were significantly higher in autistic people than non-autistic controls. IL18 appeared to have higher concentrations in the autistic than the non-autistic group, although it did not survive the FDR correction (*g* = 0.340, 95% CI [0.05, 0.63], *p* = 0.023).

#### Interferons (IFN)

Concentration of IFN-gamma was significantly higher in autistic people than in non-autistic controls (Hedges’ *g* = 0.404, 95% CI [0.09, 0.72], *p* = 0.013). IFN-alpha concentration did not differ between groups.

#### Tumour necrosis factors (TNF)

Among the TNFs, TNF-alpha was significantly higher in the autistic than in the non-autistic groups (*g* = 0.31, 95% CI [0.11, 0.51], *p* = 0.003).

#### Colony stimulating factors (CSF)

Concentrations of G-CSF, GM-CSF, and M-CSF did not differ between groups (*p*s = 0.224–820).

#### Chemokines

Only CXCL1/GRO-alpha was found significantly different between groups and was higher in the autistic than the non-autistic group (*g* = 0.364, 95% CI [0.058, 0.670], *p* = 0.02). The chemokines and CCL11/Eotaxin-1 (*g* = 0.238, 95% CI [0.001, 0.47], *p* = 0.049) and CXCL5/ENA78 (*g* = 0.352, 95% CI [0.01, 0.69], *p* = 0.041) appeared to be higher in concentrations in the autistic than the non-autistic group, but this did not survive the FDR correction.

#### Growth factors

Concentrations of growth factors, including BDNF, BNGF, EGF, FGF-2, HGF, PDGF-BB, SCF, TGF-alpha, TGF-beta, and VEGF-A, did not differ between groups (*p*s = 0.110–666; Table 1).

#### Adipokines

Concentrations of adiponectin, leptin, and resistin did not differ between the autistic and non-autistic groups (*p*s = 0.093-0.466).

#### Other cytokines

Among other cytokines, MIF was found at a higher level in the autistic than non-autistic group (*g* = 0.560, 95% CI [0.14, 0.98], *p* = 0.009).

### Sensitivity meta-analyses excluding studies with high risk of bias

The increased peripheral blood findings in autism relative to controls in interleukins IL1-beta, IL4, and IL8 remained after excluding studies with a high risk of bias. The difference of IL6 between the two groups in the sensitivity analysis became non-significant with FDR correction. With the removal of high-risk-of-bias studies, IL7 (*g* = 0.307; 95%CI [0.08, 0.53], *p* = 0.008) emerged to have significantly higher concentration in the autistic relative to the non-autistic group (Table S9, Figure S2).

After removing studies with a high risk of bias, IFN-gamma also remained higher in concentration in the autistic relative to the non-autistic groups. The difference between the two groups in TNF-alpha, CXCL1/GRO-alpha, and MIF concentrations became non-significant. Specifically in TNF-alpha, the exclusion of high-risk-of-bias studies (a high proportion of 40.5%) led to a 50% lower concentration difference between autistic and non-autistic groups. In this sensitivity meta-analysis, IL1-RA, but not MIF, was higher in concentrations in the autistic relative to the non-autistic group (*g* = 0.324, 95% CI [0.04, 0.61], *p* = 0.024) (Table S9, Figure S2).

### Moderator effects and the influence of non-significant unreported effects

Effect size heterogeneity was highly significant (*p* <0.001) across samples in most cytokines that differed between autistic and non-autistic groups—except in CXCL1/GRO-alpha and MIF. This indicated substantial variations in results across studies that could be related to the participant or study design characteristics. Examining the potential moderators of cytokine group differences separately found that higher proportion of autistic males was associated with lower IL6 (*g* = −0.037, 95% CI [−0.07, −0.004], *p* = 0.027), lower TNF-alpha (*g* = −0.030, 95% CI [−0.05, −0.009], *p* = 0.005), and higher IFN-gamma concentrations (*g* = 0.054, 95% CI [0.01, 0.09], *p* = 0.011), while blood plasma was associated with higher IL8 concentration relative to serum (*g* = 0.49, 95% CI [0.03, 0.94], *p* = 0.035). Age, control participant type, medication status, and DSM edition were not associated with cytokine concentration difference between groups. The combined moderators did not have a significant effect on the group differences of the cytokines in the main meta-analysis (all *p*s > 0.0.06), although accounting for these moderators reduced the heterogeneity level.

Eight of 98 studies were identified with NSUE [42]. Among cytokines with concentration differences in the autistic compared to the non-autistic groups, the NSUE affected group difference estimates of IL1-beta, IL6, IL8, IFN-gamma, and TNF-alpha. Including these studies in the estimation of group differences resulted in lower estimates of cytokine concentration difference between the two groups, but there was a slight reduction of the heterogeneity levels of the estimation (up to 2.1% reduction), and all findings remained significant (Table S10).

### Associations between cytokine levels and autism traits

Among 61 studies examining the association between cytokines and autism traits, 31 reported significant associations in at least one type of cytokine. All but one study [50] were conducted in children, and the remaining studies reported no significant associations (Table S11).

Focusing on studies that examined cytokines that differed between autistic and non-autistic groups and among those that investigated the relationships between interleukin levels and autism traits, seventeen examined IL1-beta, five examined IL4, eighteen examined IL6, and thirteen examined IL8. Most studies reported no correlations between interleukin levels and autism traits, with mixed findings among studies that did. A higher level of plasma IL1-beta has been reported in a subgroup of autistic children with ‘mild-to-moderate’ than ‘severe’ trait defined using CARS total score [51], but in two other studies, plasma IL1-beta concentrations were positively correlated with stereotypy [21] on the Aberrant Behavioral Checklist, and sensory sensitivity [52], indexed by the Short Sensory Profile [53]. Such mixed findings were also reported among studies reporting significant associations between IL4 [21, 28, 51] and IL8 [12, 28, 54], concentrations and autism traits based on a variety of trait measures. The interleukin IL6, in plasma or serum, stood out as the only cytokine positively correlated/associated with autism traits consistently in five studies reporting a significant association [51, 55–58], using the CARS total score as an index of autism traits. Two further studies reported higher levels of plasma IL6 with higher sensory sensitivity and stereotypy [21, 52].

Eleven studies examined IFN-gamma, among which two reported higher IFN-gamma plasma in subgroups of autistic children with ‘severe’ relative to ‘mild/moderate’ traits based on the CARS total score [51, 54]. However, one of these also reported a non-significant Spearman’s correlation between IFN-gamma and CARS total score [54], suggesting the influence of outliers in the subgroup comparison.

Eighteen studies examined TNF-alpha, with the majority showing no correlation between TNF-alpha and cytokine concentration, and those that did also showed mixed findings. Five studies reported negative associations between TNF-alpha and autism severity. Two showed a negative correlation between serum TNF-alpha level and CARS total scores [59, 60], and another showed a higher plasma TNF-alpha level among those with ‘mild/moderate’ relative to ‘severe’ autism traits [51]. Findings in the remaining two were less robust—one study presented a significant negative correlation between TNF-alpha and autism traits indexed using the SRS but not the CARS total score [61], while another reported an absence of an initially reported negative correlation with the ADOS total calibrated ‘severity’ score (CSS) when outlier data were removed [62]. Three studies reported positive associations between TNF-alpha and autism traits measured using CARS [57, 63] or Autism Behavioral Checklist total scores [64].

Among the chemokines, plasma CXCL1/GRO-alpha was examined by one study that found no relationship with levels of autism [65].

Finally, three studies examined the relationship between MIF concentration and autism traits, of which two reported positive associations, one between plasma MIF level and levels of social difficulties, imaginative skills, and the ADOS raw total scores [66], and another between serum MIF levels with autism traits as indexed by the CARS total scores [55] (See Table S11 for complete findings including other cytokines).

### Publication bias

Egger tests suggested the presence of publication bias in a few cytokines, including IL1-alpha (*p* = .0005*)*, IL17 (*p* = .0002*)*, CCL2/MCP1 (*p* = .001), and IFN-gamma (*p* = .0001), but not in other cytokines.

### Level of evidence of cytokine elevation in autism

The level of evidence for cytokines elevation in autism for IL4, IL6, IL8, IFN-gamma, TNF-alpha, CXCL1/GRO-alpha and MIF were “weak” (class IV; all *p*-values < 0.05), and among these evidence of elevated CXCL1/GRO-alpha and MIF in autism were the weakest as they were gathered from just over 160 and under 400 cases, respectively, while evidence for other cytokines was obtained from over 1000 cases each. IL1-beta was the only cytokine for which the evidence for its elevation in autistic relative to non-autistic control is at the “suggestive” level (class III; case numbers>1000 and *p*-value < 0.001) based on conventional criteria [49].

## Discussion

This systematic review and meta-analysis provide an update and new findings on the estimates of cytokine concentration in the peripheral blood of autistic people compared to non-autistic controls since the last published meta-analyses [22–25]. The work led to several notable findings: first, our main meta-analysis showed significantly higher concentrations of specific cytokines, including IL1-beta, IL4, IL6, IL8, IFN-gamma, TNF-alpha, CXCL1/GRO-alpha, and MIF, in the autistic than the non-autistic group. Second, over a third of studies identified for the meta-analysis were classified as having a high risk of bias. Third, notwithstanding the different cytokine levels between autistic and non-autistic controls found in the main meta-analysis, narrative synthesis showed no strong evidence for an association between cytokine and autism traits in autistic individuals.

Blood plasma or serum levels of predominantly IL1-beta, IL4, IL6, IL8, IFN-gamma, TNF-alpha, CXCL1/GRO-alpha, and MIF were found higher in the autistic than the non-autistic group in our main meta-analyses, with effects ranging from small to medium sizes (Hedge’s *g*s = 0.245–0.620). Of these cytokines, the elevation of IL1-beta, IL6 [22, 24, 25], and IL8 [22, 25] and IFN-gamma [22, 24] in autistic relative to non-autistic individuals has been documented in at least two prior independent meta-analyses, thus demonstrating some cross-study consistency. Except for IL4, these elevated cytokines are pro-inflammatory. Interestingly, IL6, IL8, TNF-alpha, and IFN-gamma have been found elevated in post-mortem cortical brain tissue in autistic compared to non-autistic controls [67], potentially indicating a pervasive inflammation effect implicating these cytokines in autism.

The evidence of IL1-beta elevation in autism was of the highest level among cytokines in our study based on the conventional criteria [49]. IL-1beta is a major proinflammatory cytokine released from activated microglia and has receptors throughout the nervous system. In a recent animal study, elevated IL1-beta levels during inflammation exhibited a specific effect of inducing self-withdrawal, mediated by activation of IL1R1 neurons in the dorsal raphe nucleus, relative to over twenty other major cytokine families and subfamilies [68]. Extending the preclinical finding, a recent study has shown alteration of the IL1-beta system, indexed by higher frequencies of single-nucleotide polymorphism of IL1-beta promoter (IL1-beta-511) and its allele in autistic compared to non-autistic children, associated with increased level of IL1-beta, which correlated positively with autism traits [69].

Elevated TNF-alpha [24, 25], IL4 [25], and MIF [25] among autistic individuals have also been found in previous meta-analyses, although evidence of elevated IL4 and TNF-alpha was obtained previously from one meta-analysis only after removal of statistical outliers [25]. Our finding of elevated MIF in autism represents an independent replication of the first meta-analysis to include adequate studies on this cytokine [25]. Finally, our observation of elevated peripheral blood CXCL1/GRO-alpha in autistic individuals relative to non-autistic controls extends previous meta-analytic findings, partly reflecting the increasing number of studies of this chemokine in autism in recent years [61, 70, 71]. The level of evidence for MIF and CXCL1/GRO-α being implicated in autism was among the lowest in this meta-analysis, obtained from under 500 cases, which warrants replication. The direct mechanism implicating these cytokines in autism is unclear, but in a maternal immune activation-induced animal model of autism, it has been previously shown that increased serum CXCL1/GRO-α concentration, alongside several other chemokines through gut microbiota transplantation, was associated with reduced anxiety-like and repetitive behaviours [72].

Surprisingly, over one-third of studies included in our meta-analysis were assessed as having a high risk of bias, and at least half of the studies in 30 of the 55 individual cytokine meta-analyses were rated similarly. The sensitivity meta-analyses, which excluded these high-risk-of-bias studies, led to the initial group differences in IL6, TNF-alpha, CXCL1/GRO-alpha, and MIF losing their statistical significance. These could suggest that the original group differences in certain cytokines may have been driven by high-risk-of-bias studies. For example, in TNF-alpha, excluding such studies—which made up over 40% of those in the main meta-analysis—reduced the concentration difference between autistic and non-autistic groups by about 50%, bringing the effect size below 0.2. It should be noted that removing studies from cytokine meta-analyses generally reduces statistical power. This can obscure potential elevations in autism-related cytokines; for instance, IL6 lost statistical significance in the sensitivity meta-analysis after FDR correction, while MIF became altogether non-significant. In other cases, such as CXCL1/GRO-alpha, excluding high-risk-of-bias studies left too few studies to conduct a meta-analysis for.

The sensitivity analysis was also associated with the emerging finding of elevated IL7 and IL1-RA levels in autism. The elevated peripheral blood IL7 in autism was found in a prior meta-analysis [25], but the elevated blood IL1-RA in autism was a novel meta-analytic finding. Polymorphisms in the IL1-RA genotype variants, in addition to the alteration of IL1-beta system, were correlated positively with autism traits in autistic children [69]. Serum levels of IL1-RA and IL1-beta have been found to be positively correlated [65, 69]. Given IL1-RA has antagonistic influence on the effects of IL1-beta, and other cytokines such as IL1-alpha, our findings of higher but more limited IL1-RA level could reflect a weaker negative regulatory feedback by IL1-RA on the pro-inflammatory IL1-beta in autism [65, 69]. Such interpretation, however, is limited by the fact that our meta-analytic estimation of the elevation of IL1-RA and IL1-beta levels in autism was based on a different pool of studies.

Overall, our meta-analytic findings comprised broader and stronger evidence for the elevations of the predominantly pro-inflammatory cytokines in autism, including IL1-beta, IL8, and the key Th1 cytokine IFN-gamma (i.e., the cytokine produced by T helper 1 cells), which was replicated in the sensitivity meta-analyses. This supports the assertion of a pro-inflammatory dominance in this population. These cytokine findings contrast with the more limited elevation of the anti-inflammatory cytokine IL4 in autism, potentially reflecting an imbalance between pro- and anti-inflammatory signalling, as has been observed across various immune cells in autism [73–75]. Weaker findings in the meta-analysis, which either emerged or lost significance in sensitivity analyses, similarly demonstrated the predominance of pro-inflammatory cytokines, i.e., IL6, IL7, TNF-gamma, CXCL1/GRO-alpha, and MIF, which were broadly elevated, alongside an anti-inflammatory cytokine IL1-RA. Together with existing findings in the wider literature, our meta-analytic findings support the idea of a potentially more nuanced interplay among activating and inhibiting cytokines that modulate immune responses in autism.

Despite the observed serum/plasma level differences in IL1-beta, IL4, IL6, IL8, IFN-gamma, TNF-alpha, CXCL1/GRO-alpha, and MIF between autistic and non-autistic groups, most studies examining the relationship between cytokine levels and autism traits in autistic individuals have reported no significant association, while the few that have identified one often show inconsistent or mixed results across studies. Our comprehensive synthesis of evidence involving multiple trait measures and informants, including parent/carer (e.g., the CARS, ADI-R) or clinical observation (e.g., the ADOS), variation of scoring systems within measure (e.g., domain and total scores for the SRS and the ADI-R among others, calibrated severity or raw scores for the ADOS) offered no coherent findings of an association between cytokine levels and autism traits within the group of autistic individuals. This might suggest that cytokine level differences reflect group-level biological variation rather than accounting for dimensional variation of autism traits in the autistic population.

Our finding of the lack of association between cytokine levels and autism traits among autistic individuals, especially in IL1-beta, IL6, IL8, IFN-gamma, and TNF-alpha, which have been supported by at least ten studies per cytokine, would reduce their likelihood for having a causal role in autistic traits throughout development, Instead, their involvement might be limited in specific time point during development, leading to a persistent group differences between those who are autistic and non-autistic, for instance during the early years. Indeed, recent studies from two large cohorts of children show that IL1-beta [76], IL6, and IL8 [77] are elevated at the neonatal stage in children who later develop autism, supporting the notion that atypical cytokine profiles may be present from birth and contribute to early life immune alterations or dysregulation in autism. Atypical elevation of cytokines could also reflect state-dependent factors, such as inflammation, stress, or co-occurring medical problems. Autistic children and adolescents have an increased likelihood of experiencing daily stressors, and mental health difficulties such as depression and anxiety [78, 79], of which development and progression are known to be associated with increased levels of peripheral blood pro-inflammatory cytokines such as IL6, IL1-beta, and TNF-alpha [80, 81]. Inflammatory diseases such as gastrointestinal inflammation, autoimmune, and atopic diseases occur at higher rates in autism than in non-autistic individuals [6, 82, 83].

The robustness of these interpretations is, however, constrained not only by the substantial heterogeneity evident across studies but also by the potential inflation of effect-size estimates in cytokine group comparisons. In our meta-analyses, some cytokine group differences were moderated by sex (i.e., lower level of IL6 and TNF-alpha with higher proportion of male participants) and sample type (i.e., higher IL8 with in blood plasma than serum), while accounting for the combined effects of moderators, while not significantly associated with cytokine levels, reduce the level of heterogeneity in the findings. The findings of our assessment of risk of bias among studies underscore the need for better quality studies in the field. Such studies may reduce heterogeneity of findings among the cytokines, given their removal was associated with lower heterogeneity levels across our meta-analyses. Finally, our findings suggest that poor reporting of statistically non-significant findings can lead to inflated meta-analytic summaries if these findings are not properly accounted for.

The strengths of our meta-analysis include a comprehensive literature search strategy across multiple databases and a thorough examination of explanatory factors contributing to heterogeneity. This includes assessing the risk of bias across studies, considering non-significant unreported effects [42], and evaluating a range of potential moderators. However, this strength is constrained by the quality of evidence in the field, where over half of the published studies were found to have a high risk of bias based on our systematic evaluation. Indeed, according to conventional criteria [49], the level of evidence for cytokine elevation in autism, with the exception of IL1-beta, was mostly “weak”. Attempts to address this limitation by conducting sensitivity meta-analyses that excluded studies with a high risk of bias, despite having expanded the range of cytokines implicated in autism in the meta-analysis, may have reduced the power of the meta-analyses and potentially led to missed subtler group differences. Finally, the investigation into the specificity of cytokine profile alterations was also limited by a lack of detailed information on the participants’ sex, medication intake, or co-occurring psychiatric conditions that were not always consistently reported across studies.

This meta-analysis represents the most recent and comprehensive summary analysis of cytokine profiles in autistic people compared to non-autistic controls. Our stronger group difference findings support the notion of a predominant pro-inflammatory cytokine in autism, while some of the weaker group-difference findings suggest a more nuanced involvement of both pro- and anti-inflammatory cytokines in the modulatory immune responses in autism. The narrative synthesis found no strong evidence for an association between cytokines and autism traits in autistic individuals. Altogether our findings suggest that cytokine level differences reflect group-level biological variation rather than variation of autism traits in the autistic population. Such findings were presented against the backdrop of the substantial proportion of studies with high risk of bias in this field, underlining the need for systematic assessment of the risk of bias of studies included in future meta-analyses and broadly pointing towards the need for more high-quality studies in this field, before a stronger conclusion of the role of cytokines in autism could be drawn.

## Supplementary information

Supplementary information to Volk, Lukito et al. (2026). Atypical Cytokine Profiles in Autism Spectrum Condition: A Comprehensive Systematic Review and Meta-analysis Including 54 Cytokines.
