# Supplementary material for: Atypical cytokine profiles in people on the autism spectrum: a comprehensive systematic review and meta-analysis including 54 cytokines
Source: Mol Psychiatry. 2026 May 28;31(10):5775–86. doi: 10.1038/s41380-026-03659-4 (PMC13569442; doi:10.1038/s41380-026-03659-4)
Supplement: Supplementary file 1 [file 41380_2026_3659_MOESM1_ESM.docx]

Supplementary information to Volk, Lukito et al. (2026). Atypical Cytokine Profiles in Autism Spectrum Condition: A Comprehensive Systematic Review and Meta-analysis Including 54 Cytokines.

**Table of Content**

| Methods | Literature Search Strategy | 1 |
| --- | --- | --- |
| Table S1 | Ovid Embase, Medline, and APA PsycINFO Search Strategy | 1 |
| Table S2 | Scopus Search Strategy | 2 |
| Table S3 | Web of Science Search Strategy | 3 |
| Table S4 | PRISMA Abstract and Review Checklist | 4 |
| Table S5 | Adapted Newcastle-Ottawa Quality Assessment Scale for Cross-Sectional Studies | 7 |
| Table S6 | Risk of Bias Assessment according to the Adapted Newcastle-Ottawa Scale | 8 |
| Table S7 | List of Excluded Studies and Exclusion Reasons | 11 |
| Table S8 | Table of Characteristics of Included Studies | 16 |
| Figure S1 | Forest Plots of Significant Meta-analytic Findings Using All Available Data | 25 |
| Table S9 | Meta-analytic Findings after Removal of Studies with High Risk of Bias | 29 |
| Figure S2 | Forest Plots of Significant Meta-analytic Findings After Removal of Studies with High Risk of Bias | 31 |
| Table S10 | Meta-analytic Findings including Studies Providing Non-Significant Unreported Effects (NSUE). | 33 |
| Table S11 | Publications Examining the Association between Cytokine Levels and Autistic Traits | 34 |

**Literature Search Strategy**

The systematic literature search strategy combined key terms related to autism (i.e., autis* *or* ASD *or* “pervasive development* disorder*” *or* Asperger*) *and* cytokines (e.g., cytokin* *or* interleukin* *or* chemokin*) and conducted in the databases EMBASE, MEDLINE and APA PsycINFO, on the Ovid^®^ platform, and Web of Science^®^ and SCOPUS^®^. The first wave of search was carried out from the databases’ inceptions to 9th June 2021, a second search using identical terms was conducted from published studies from 1^st^ January 2021 to 30^th^ December 2022, and a third wave of search was conducted from 1^st^ January 2023 to 17^th^ March 2025. These were supplemented by manual reference search using past meta-analyses^1-3^ (see complete search term on Tables S1-3 as applied on 9th June 2021). The protocol for this systematic review and meta-analysis was pre-registered in PROSPERO (CRD42022304200). We observed the Preferred Reporting Items for Systematic Reviews and Meta-Analysis (PRISMA2020) guidelines^4^.

**Table S1. Ovid Embase, Medline, and APA PsycINFO Search Strategy**

| No | Query | Articles |
| --- | --- | --- |
| 1 | autis*.mp. [mp=ti, ab, hw, tn, ot, dm, mf, dv, kw, fx, dq, nm, kf, ox, px, rx, ui, sy, tc, id, tm, mh] | 200205 |
| 2 | ASD.mp. [mp=ti, ab, hw, tn, ot, dm, mf, dv, kw, fx, dq, nm, kf, ox, px, rx, ui, sy, tc, id, tm, mh] | 85731 |
| 3 | "pervasive development* disorder*".mp. [mp=ti, ab, hw, tn, ot, dm, mf, dv, kw, fx, dq, nm, kf, ox, px, rx, ui, sy, tc, id, tm, mh] | 9295 |
| 4 | Asperger*.mp. [mp=ti, ab, hw, tn, ot, dm, mf, dv, kw, fx, dq, nm, kf, ox, px, rx, ui, sy, tc, id, tm, mh] | 13056 |
| 5 | 1 or 2 or 3 or 4 | 224542 |
| 6 | cytokin*.mp. [mp=ti, ab, hw, tn, ot, dm, mf, dv, kw, fx, dq, nm, kf, ox, px, rx, ui, sy, tc, id, tm, mh] | 1159912 |
| 7 | interleukin*.mp. [mp=ti, ab, hw, tn, ot, dm, mf, dv, kw, fx, dq, nm, kf, ox, px, rx, ui, sy, tc, id, tm, mh] | 1131730 |
| 8 | chemokin*.mp. [mp=ti, ab, hw, tn, ot, dm, mf, dv, kw, fx, dq, nm, kf, ox, px, rx, ui, sy, tc, id, tm, mh] | 265930 |
| 9 | interferon*.mp. [mp=ti, ab, hw, tn, ot, dm, mf, dv, kw, fx, dq, nm, kf, ox, px, rx, ui, sy, tc, id, tm, mh] | 624566 |
| 10 | "tumo$r necro* factor*".mp. [mp=ti, ab, hw, tn, ot, dm, mf, dv, kw, fx, dq, nm, kf, ox, px, rx, ui, sy, tc, id, tm, mh] | 638608 |
| 11 | inflammat*.mp. [mp=ti, ab, hw, tn, ot, dm, mf, dv, kw, fx, dq, nm, kf, ox, px, rx, ui, sy, tc, id, tm, mh] | 2803791 |
| 12 | "colony-stimulat* factor*".mp. [mp=ti, ab, hw, tn, ot, dm, mf, dv, kw, fx, dq, nm, kf, ox, px, rx, ui, sy, tc, id, tm, mh] | 170717 |
| 13 | "transform* growth factor*".mp. [mp=ti, ab, hw, tn, ot, dm, mf, dv, kw, fx, dq, nm, kf, ox, px, rx, ui, sy, tc, id, tm, mh] | 271815 |
| 14 | ("IL-1alpha" or "IL-1-alpha" or "IL1alpha" or "IL-1beta" or "IL-1-beta" or "IL1beta" or "IL-6" or "IL6” or IL-8" or "IL8” or IL-10" or "IL10" or "IL-12" or "IL12" or "IFNalpha" or "IFN-alpha" or "IFNbeta" or "IFN-beta" or "TNFalpha" or "TNF-alpha" or "G-CSF" or "GCSF” or M-CSF" or "MCSF" or "GM-CSF" or "GMCSF” or “TGFbeta" or "TGF-beta").mp. [mp=ti, ab, hw, tn, ot, dm, mf, dv, kw, fx, dq, nm, kf, ox, px, rx, ui, sy, tc, id, tm, mh] | 932054 |
| 15 | (CCL11 or Eotaxin or “MCP-1” or MCP1 or CCL2).mp. [mp=ti, ab, hw, tn, ot, dm, mf, dv, kw, fx, dq, nm, kf, ox, px, rx, ui, sy, tc, id, tm, mh] | 84604 |
| 16 | or/6-15 | 4535553 |
| 17 | 5 and 16 | 6658 |
| 18 | (exp animal/ or exp invertebrate/ or animal experiment/ or animal model/ or exp plant/ or exp fungus/) not exp human/ | 11467222 |
| 19 | 17 not 18 | 5578 |
| 20 | editorial/ or exp editorial/ or letter/ or exp letter/ [mp=ti, ab, hw, tn, ot, dm, mf, dv, kw, fx, dq, nm, kf, ox, px, rx, ui, sy, tc, id, tm, mh] | 3495106 |
| 21 | 19 not 20 | 5358 |
| 22 | remove duplicates from 21 | 3687 |

**Table S2. Scopus Search Strategy**

| No | Query | Articles |
| --- | --- | --- |
| 1 | TITLE-ABS-KEY ( autis*  OR  asd  OR  "pervasive development* disorder*"  OR  asperger* ) | 107,095 |
| 2 | TITLE-ABS-KEY ( cytokin* OR interleukin* OR chemokin* OR interferon* OR "tumo*r necro* factor*" OR inflammat* OR "colony-stimulat* factor*" OR "transform* growth factor*" OR "IL-1alpha" OR "IL-1-alpha" OR "IL1alpha" OR "IL-1beta" OR "IL-1-beta" OR "IL1beta" OR "IL-6" OR "IL6" OR "IL-8" OR "IL8" OR "IL-10" OR "IL10" OR "IL-12" OR "IL12" OR "IFNalpha" OR "IFN-alpha" OR "IFNbeta" OR "IFN-beta" OR "TNFalpha" OR "TNF-alpha" OR "G-CSF" OR "GCSF" OR "M-CSF" OR "MCSF" OR "GM-CSF" OR "GMCSF" OR "TGFbeta" OR "TGF-beta" OR "CCL11" OR eotaxin OR "MCP-1" OR "MCP1" OR "CCL2" ) | 2,348,768 |
| 3 | ( TITLE-ABS-KEY ( cytokin*  OR  interleukin*  OR  chemokin*  OR  interferon*  OR  "tumo*r necro* factor*"  OR  inflammat*  OR  "colony-stimulat* factor*"  OR  "transform* growth factor*"  OR  "IL-1alpha"  OR  "IL-1-alpha"  OR  "IL1alpha"  OR  "IL-1beta"  OR  "IL-1-beta"  OR  "IL1beta"  OR  "IL-6"  OR  "IL6"  OR  "IL-8"  OR  "IL8"  OR  "IL-10"  OR  "IL10"  OR  "IL-12"  OR  "IL12"  OR  "IFNalpha"  OR  "IFN-alpha"  OR  "IFNbeta"  OR  "IFN-beta"  OR  "TNFalpha"  OR  "TNF-alpha"  OR  "G-CSF"  OR  "GCSF"  OR  "M-CSF"  OR  "MCSF"  OR  "GM-CSF"  OR  "GMCSF"  OR  "TGFbeta"  OR  "TGF-beta"  OR  "CCL11"  OR  eotaxin  OR  "MCP-1"  OR  "MCP1"  OR  "CCL2" ) )  AND  ( TITLE-ABS-KEY ( autis*  OR  asd  OR  "pervasive development* disorder*"  OR  asperger* ) ) | 3,325 |
| 4 | INDEXTERMS ( animals  OR  animal )  AND NOT  INDEXTERMS ( humans  OR  human ) | 4,944,431 |
| 5 | INDEXTERMS ( editorials  OR  editorial  OR  letters  OR  letter ) | 1,715,760 |
| 6 | ( ( TITLE-ABS-KEY ( cytokin*  OR  interleukin*  OR  chemokin*  OR  interferon*  OR  "tumo*r necro* factor*"  OR  inflammat*  OR  "colony-stimulat* factor*"  OR  "transform* growth factor*"  OR  "IL-1alpha"  OR  "IL-1-alpha"  OR  "IL1alpha"  OR  "IL-1beta"  OR  "IL-1-beta"  OR  "IL1beta"  OR  "IL-6"  OR  "IL6"  OR  "IL-8"  OR  "IL8"  OR  "IL-10"  OR  "IL10"  OR  "IL-12"  OR  "IL12"  OR  "IFNalpha"  OR  "IFN-alpha"  OR  "IFNbeta"  OR  "IFN-beta"  OR  "TNFalpha"  OR  "TNF-alpha"  OR  "G-CSF"  OR  "GCSF"  OR  "M-CSF"  OR  "MCSF"  OR  "GM-CSF"  OR  "GMCSF"  OR  "TGFbeta"  OR  "TGF-beta"  OR  "CCL11"  OR  eotaxin  OR  "MCP-1"  OR  "MCP1"  OR  "CCL2" ) )  AND  ( TITLE-ABS-KEY ( autis*  OR  asd  OR  "pervasive development* disorder*"  OR  asperger* ) ) )  AND NOT  ( INDEXTERMS ( animals  OR  animal )  AND NOT  INDEXTERMS ( humans  OR  human ) ) | 2,886 |
| 7 | ( ( ( TITLE-ABS-KEY ( cytokin*  OR  interleukin*  OR  chemokin*  OR  interferon*  OR  "tumo*r necro* factor*"  OR  inflammat*  OR  "colony-stimulat* factor*"  OR  "transform* growth factor*"  OR  "IL-1alpha"  OR  "IL-1-alpha"  OR  "IL1alpha"  OR  "IL-1beta"  OR  "IL-1-beta"  OR  "IL1beta"  OR  "IL-6"  OR  "IL6"  OR  "IL-8"  OR  "IL8"  OR  "IL-10"  OR  "IL10"  OR  "IL-12"  OR  "IL12"  OR  "IFNalpha"  OR  "IFN-alpha"  OR  "IFNbeta"  OR  "IFN-beta"  OR  "TNFalpha"  OR  "TNF-alpha"  OR  "G-CSF"  OR  "GCSF"  OR  "M-CSF"  OR  "MCSF"  OR  "GM-CSF"  OR  "GMCSF"  OR  "TGFbeta"  OR  "TGF-beta"  OR  "CCL11"  OR  eotaxin  OR  "MCP-1"  OR  "MCP1"  OR  "CCL2" ) )  AND  ( TITLE-ABS-KEY ( autis*  OR  asd  OR  "pervasive development* disorder*"  OR  asperger* ) ) )  AND NOT  ( INDEXTERMS ( animals  OR  animal )  AND NOT  INDEXTERMS ( humans  OR  human ) ) )  AND NOT  ( INDEXTERMS ( editorials  OR  editorial  OR  letters  OR  letter ) ) | 2,719 |

**Table S3. Web of Science search on 09.06.2021**

| No | Query | Articles |
| --- | --- | --- |
| 1 | ALL FIELDS: (autis* OR ASD OR "pervasive development* disorder*" OR Asperger*)  *Indexes=SCI-EXPANDED, SSCI, A&HCI, ESCI Timespan=All years* | 99871 |
| 2 | ALL FIELDS: (cytokin* OR interleukin* OR chemokin* OR interferon* OR "tumo$r necro* factor*" OR inflammat* OR "colony-stimulat* factor*" OR "transform* growth factor*" OR "IL-1alpha" OR "IL-1-alpha" OR "IL1alpha" OR "IL-1beta" OR "IL-1-beta" OR "IL1beta" OR "IL-6" OR "IL6" OR "IL-8" OR "IL8" OR "IL-10" OR "IL10" OR "IL-12" OR "IL12" OR "IFNalpha" OR "IFN-alpha" OR "IFNbeta" OR "IFN-beta" OR "TNFalpha" OR "TNF-alpha" OR "G-CSF" OR "GCSF" OR "M-CSF" OR "MCSF" OR "GM-CSF" OR "GMCSF" OR "TGFbeta" OR "TGF-beta" OR "CCL11" OR eotaxin OR "MCP-1" OR "MCP1" OR "CCL2")  *Indexes=SCI-EXPANDED, SSCI, A&HCI, ESCI Timespan=All years* | 1969359 |
| 3 | ALL FIELDS: (autis* OR ASD OR "pervasive development* disorder*" OR Asperger*) *Indexes=SCI-EXPANDED, SSCI, A&HCI, ESCI Timespan=All year* | 2775 |

**Table S4. PRISMA Abstract and Review Checklist**

| **Section and Topic** | **Item #** | **Abstract Checklist items** | **Reported (Yes/No)** |
| --- | --- | --- | --- |
| **TITLE** | | |  |
| Title | 1 | Identify the report as a systematic review. | Yes |
| **BACKGROUND** | | |  |
| Objectives | 2 | Provide an explicit statement of the main objective(s) or question(s) the review addresses. | Yes |
| **METHODS** | | |  |
| Eligibility criteria | 3 | Specify the inclusion and exclusion criteria for the review. | No (in main text) |
| Information sources | 4 | Specify the information sources (e.g. databases, registers) used to identify studies and the date when each was last searched. | Yes |
| Risk of bias | 5 | Specify the methods used to assess risk of bias in the included studies. | Yes |
| Synthesis of results | 6 | Specify the methods used to present and synthesise results. | Yes |
| **RESULTS** | | |  |
| Included studies | 7 | Give the total number of included studies and participants and summarise relevant characteristics of studies. | Yes |
| Synthesis of results | 8 | Present results for main outcomes, preferably indicating the number of included studies and participants for each. If meta-analysis was done, report the summary estimate and confidence/credible interval. If comparing groups, indicate the direction of the effect (i.e. which group is favoured). | Yes |
| **DISCUSSION** | | |  |
| Limitations of evidence | 9 | Provide a brief summary of the limitations of the evidence included in the review (e.g. study risk of bias, inconsistency and imprecision). | Yes |
| Interpretation | 10 | Provide a general interpretation of the results and important implications. | Yes |
| **OTHER** | | |  |
| Funding | 11 | Specify the primary source of funding for the review. | No (in main text) |
| Registration | 12 | Provide the register name and registration number. | No (in main text) |
| **Section and Topic** | **Item #** | **Review Checklist items** | **Location where item is reported** |
| **TITLE** | | |  |
| Title | 1 | Identify the report as a systematic review. | See title (page 1) |
| **ABSTRACT** | | |  |
| Abstract | 2 | See the PRISMA 2020 for Abstracts checklist. | See Abstract (page 2) |
| **INTRODUCTION** | | |  |
| Rationale | 3 | Describe the rationale for the review in the context of existing knowledge. | See introduction, third paragraph. |
| Objectives | 4 | Provide an explicit statement of the objective(s) or question(s) the review addresses. | See introduction, last paragraph. |
| **METHODS** | | |  |
| Eligibility criteria | 5 | Specify the inclusion and exclusion criteria for the review and how studies were grouped for the syntheses. | See 2.2. |
| Information sources | 6 | Specify all databases, registers, websites, organisations, reference lists and other sources searched or consulted to identify studies. Specify the date when each source was last searched or consulted. | See 2.1. |
| Search strategy | 7 | Present the full search strategies for all databases, registers and websites, including any filters and limits used. | See Tables S1, S2, S3. |
| Selection process | 8 | Specify the methods used to decide whether a study met the inclusion criteria of the review, including how many reviewers screened each record and each report retrieved, whether they worked independently, and if applicable, details of automation tools used in the process. | See 2.3. |
| Data collection process | 9 | Specify the methods used to collect data from reports, including how many reviewers collected data from each report, whether they worked independently, any processes for obtaining or confirming data from study investigators, and if applicable, details of automation tools used in the process. | See 2.4. |
| Data items | 10a | List and define all outcomes for which data were sought. Specify whether all results that were compatible with each outcome domain in each study were sought (e.g. for all measures, time points, analyses), and if not, the methods used to decide which results to collect. | See 2.4. |
|  | 10b | List and define all other variables for which data were sought (e.g. participant and intervention characteristics, funding sources). Describe any assumptions made about any missing or unclear information. | See 2.4. and 2.6. |
| Study risk of bias assessment | 11 | Specify the methods used to assess risk of bias in the included studies, including details of the tool(s) used, how many reviewers assessed each study and whether they worked independently, and if applicable, details of automation tools used in the process. | See 2.5, Table S5. |
| Effect measures | 12 | Specify for each outcome the effect measure(s) (e.g. risk ratio, mean difference) used in the synthesis or presentation of results. | See 2.6. |
| Synthesis methods | 13a | Describe the processes used to decide which studies were eligible for each synthesis (e.g. tabulating the study intervention characteristics and comparing against the planned groups for each synthesis (item #5)). | See Table S7. |
|  | 13b | Describe any methods required to prepare the data for presentation or synthesis, such as handling of missing summary statistics, or data conversions. | See 2.6. |
|  | 13c | Describe any methods used to tabulate or visually display results of individual studies and syntheses. | See 2.6. |
|  | 13d | Describe any methods used to synthesize results and provide a rationale for the choice(s). If meta-analysis was performed, describe the model(s), method(s) to identify the presence and extent of statistical heterogeneity, and software package(s) used. | See 2.6. |
|  | 13e | Describe any methods used to explore possible causes of heterogeneity among study results (e.g. subgroup analysis, meta-regression). | See 2.6. |
|  | 13f | Describe any sensitivity analyses conducted to assess robustness of the synthesized results. | See 2.6. |
| Reporting bias assessment | 14 | Describe any methods used to assess risk of bias due to missing results in a synthesis (arising from reporting biases). | See 2.6. |
| Certainty assessment | 15 | Describe any methods used to assess certainty (or confidence) in the body of evidence for an outcome. | See 2.6. |
| **RESULTS** | | |  |
| Study selection | 16a | Describe the results of the search and selection process, from the number of records identified in the search to the number of studies included in the review, ideally using a flow diagram. | See 3.1 and Figure 1. |
|  | 16b | Cite studies that might appear to meet the inclusion criteria, but which were excluded, and explain why they were excluded. | See Table S7 |
| Study characteristics | 17 | Cite each included study and present its characteristics. | See Table S8. |
| Risk of bias in studies | 18 | Present assessments of risk of bias for each included study. | See Table S6. |
| Results of individual studies | 19 | For all outcomes, present, for each study: (a) summary statistics for each group (where appropriate) and (b) an effect estimate and its precision (e.g. confidence/credible interval), ideally using structured tables or plots. | See Table 1. |
| Results of syntheses | 20a | For each synthesis, briefly summarise the characteristics and risk of bias among contributing studies. | See Figure 3. |
|  | 20b | Present results of all statistical syntheses conducted. If meta-analysis was done, present for each the summary estimate and its precision (e.g. confidence/credible interval) and measures of statistical heterogeneity. If comparing groups, describe the direction of the effect. | See Figure S1. |
|  | 20c | Present results of all investigations of possible causes of heterogeneity among study results. | See 3.6. and Table S10 |
|  | 20d | Present results of all sensitivity analyses conducted to assess the robustness of the synthesized results. | See 3.5. and Table S9 |
| Reporting biases | 21 | Present assessments of risk of bias due to missing results (arising from reporting biases) for each synthesis assessed. | See 3.8. |
| Certainty of evidence | 22 | Present assessments of certainty (or confidence) in the body of evidence for each outcome assessed. | See Figure S1 |
| **DISCUSSION** | | |  |
| Discussion | 23a | Provide a general interpretation of the results in the context of other evidence. | See Discussion. |
|  | 23b | Discuss any limitations of the evidence included in the review. | See Discussion. |
|  | 23c | Discuss any limitations of the review processes used. | No. |
|  | 23d | Discuss implications of the results for practice, policy, and future research. | See Discussion, last paragraph |
| **OTHER INFORMATION** | | |  |
| Registration and protocol | 24a | Provide registration information for the review, including register name and registration number, or state that the review was not registered. | See 2.1. |
|  | 24b | Indicate where the review protocol can be accessed, or state that a protocol was not prepared. | See 2.1 |
|  | 24c | Describe and explain any amendments to information provided at registration or in the protocol. | NA |
| Support | 25 | Describe sources of financial or non-financial support for the review, and the role of the funders or sponsors in the review. | See 5. |
| Competing interests | 26 | Declare any competing interests of review authors. | See 6. |
| Availability of data, code and other materials | 27 | Data extracted from included studies are available in the supplementary. | See Supplementary data. |

**Table S5. Adapted Newcastle-Ottawa Quality Assessment Scale for Cross-Sectional Studies**

This Newcastle-Ottawa Scale was adapted from Ribeiro et al. 2020^5^. Risk of bias was judged as low, medium or high.

*Sample selection (maximum 3 points)*

1) Sample size: 1 point was given if sample sizes of both groups were comparable in size and ASD cases and controls were not related

2) 1 point was given if it was clearly stated that ASD diagnosis was given according to DSM/ICD guidelines or confirmed by ADOS and ADI

3) 1 point was given if there were clearly stated exclusion criteria for participants in both groups (clearly stated the exclusion of participants with neurological comorbidities).

*Comparability of cases and control participants (maximum 2 points)*

Subjects in different outcome groups are comparable, based on study design or analysis. Confounding factors are controlled.

4) point was given if the two group (ASD and controls) were matched for age and gender/no significant difference in age/sex between both groups

5) 1 point was given if for both groups there was information given about comorbid psychiatric diseases influencing cytokine concentrations.

*Assessments of cytokine level (maximum 3 points)*

6) 1 point was given if the method of measurement of cytokine concentrations was clearly described (e.g. which cytokines were measured, which kit was used)

7) 1 point was given If it was clearly stated that ASD cases and controls were both not taking any medication by the time of blood collection*.*

8) 1 point was given if the outcome was reported appropriate and complete, and the difference in cytokine concentrations was presented for each cytokine analyzed, including mean values/ median values and standard deviations/IQRs.

*Studies that scored a total of 8 or 7 points was considered to have a low risk of bias; 5 or 6 points were considered to have a medium risk of bias; 4 points or less were considered to have a high risk of bias.*

**Table S6. Risk of Bias Assessment according to the Adapted Newcastle-Ottawa Scale**

| **Authors (Year)** | **1** | **2** | **3** | **4** | **5** | **6** | **7** | **8** | **Total score** | **Risk of bias** |
| --- | --- | --- | --- | --- | --- | --- | --- | --- | --- | --- |
| Abdala et al. (2019)^6^ | 0 | 0 | 0 | 0 | 0 | 1 | 0 | 1 | 2 | High |
| Abd-Allah et al. (2020)^7^ | 1 | 1 | 1 | 1 | 1 | 1 | 0 | 1 | 7 | Low |
| Abdel-Salam et al. (2017)^8^ | 1 | 1 | 0 | 1 | 0 | 1 | 1 | 1 | 6 | Medium |
| Al-ayadhi & Mostafa (2012)^9^ | 1 | 1 | 1 | 1 | 0 | 1 | 0 | 1 | 6 | Medium |
| Al-ayadhi & Mostafa (2013)^10^ | 0 | 1 | 1 | 1 | 0 | 1 | 0 | 1 | 5 | Medium |
| Al-Bishri et al. (2023)^11^ | 1 | 1 | 1 | 1 | 0 | 1 | 0 | 1 | 6 | Medium |
| Almashhady et al. (2025)^12^ | 1 | 0 | 0 | 1 | 0 | 1 | 0 | 1 | 4 | High |
| Alshammary et al. (2023)^13^ | 0 | 0 | 0 | 0 | 0 | 1 | 0 | 1 | 2 | High |
| Alzghoul et al. (2019)^14^ | 1 | 1 | 0 | 1 | 0 | 1 | 0 | 0 | 4 | High |
| Anastasescu et al. (2024)^15^ | 0 | 0 | 0 | 0 | 0 | 1 | 1 | 0 | 2 | High |
| Anindya et al. (2025)^16^ | 1 | 1 | 1 | 0 | 0 | 1 | 0 | 0 | 4 | High |
| Artık et al. (2023)^17^ | 1 | 1 | 1 | 1 | 1 | 1 | 1 | 1 | 8 | Low |
| Ashaat et al. (2017)^18^ | 1 | 1 | 0 | 1 | 0 | 1 | 0 | 1 | 5 | Medium |
| Ashwood et al. (2008)^19^ | 0 | 1 | 0 | 0 | 0 | 1 | 0 | 1 | 3 | High |
| Ashwood et al. (2008)^20^ | 0 | 1 | 0 | 0 | 0 | 1 | 0 | 1 | 3 | High |
| Ashwood et al. (2011)^21^ | 1 | 1 | 0 | 1 | 0 | 1 | 1 | 1 | 6 | Medium |
| Ashwood et al. (2011)^22^ | 0 | 1 | 0 | 0 | 0 | 1 | 1 | 1 | 4 | High |
| Barbosa et al. (2015)^23^ | 0 | 1 | 0 | 1 | 0 | 1 | 0 | 1 | 4 | High |
| Basheer et al. (2018)^24^ | 0 | 1 | 0 | 1 | 1 | 1 | 1 | 0 | 5 | Medium |
| Belica et al. (2023)^25^ | 0 | 1 | 0 | 0 | 0 | 1 | 0 | 1 | 3 | High |
| Bilgiç et al. (2024)^26^ | 0 | 0 | 1 | 0 | 0 | 1 | 1 | 1 | 4 | High |
| Blardi et al. (2010) ^27^ | 1 | 1 | 1 | 1 | 1 | 1 | 0 | 1 | 7 | Low |
| Cao et al. (2021) ^28^ | 1 | 1 | 1 | 0 | 1 | 1 | 1 | 1 | 7 | Low |
| Carissimi et al. (2019)^29^ | 0 | 1 | 0 | 0 | 0 | 1 | 0 | 1 | 3 | High |
| Carpita et al. (2023)^30^ | 1 | 1 | 1 | 0 | 0 | 1 | 0 | 1 | 5 | Medium |
| Croonenberghs et al. (2002)^31^ | 1 | 1 | 1 | 1 | 0 | 1 | 1 | 1 | 7 | Low |
| Denney et al. (1996)^32^ | 1 | 1 | 0 | 1 | 0 | 1 | 0 | 1 | 5 | Medium |
| El Gohary et al. (2015)^33^ | 1 | 1 | 1 | 1 | 1 | 0 | 1 | 1 | 7 | Low |
| El Wakkad et al. (2006)^34^ | 1 | 1 | 1 | 1 | 0 | 1 | 0 | 1 | 6 | Medium |
| El-Ansary et al. (2011)^35^ | 0 | 1 | 1 | 1 | 1 | 0 | 0 | 1 | 5 | Medium |
| El-Ansary et al. (2012)^36^ | 1 | 1 | 0 | 1 | 1 | 1 | 0 | 1 | 6 | Medium |
| El-Ansary et al. (2014)^37^ | 1 | 1 | 1 | 1 | 1 | 1 | 0 | 1 | 7 | Low |
| El-Ansary et al. (2016)^38^ | 1 | 1 | 1 | 1 | 1 | 1 | 0 | 1 | 7 | Low |
| Emanuele et al. (2010)^39^ | 1 | 1 | 0 | 1 | 0 | 1 | 1 | 1 | 6 | Medium |
| Enstrom et al. (2008)^40^ | 0 | 1 | 0 | 1 | 0 | 1 | 0 | 1 | 4 | High |
| Enstrom et al. (2008)^41^ | 0 | 1 | 0 | 0 | 0 | 1 | 0 | 1 | 3 | High |
| Ferencova et al. (2023)^42^ | 1 | 1 | 1 | 1 | 1 | 1 | 1 | 0 | 7 | Low |
| Filippova et al. (2023)^43^ | 0 | 1 | 0 | 0 | 0 | 1 | 0 | 1 | 3 | High |
| Fujita-Shimizo et al. (2010)^44^ | 1 | 0 | 1 | 1 | 1 | 1 | 1 | 1 | 7 | Low |
| Ghaffari et al. (2016)^45^ | 1 | 1 | 0 | 1 | 1 | 1 | 1 | 1 | 7 | Low |
| Gomez-Fernandez et al. (2018)^46^ | 1 | 1 | 1 | 1 | 0 | 1 | 1 | 1 | 7 | Low |
| Grigorenko et al. (2008)^47^ | 0 | 1 | 0 | 0 | 0 | 1 | 0 | 1 | 3 | High |
| Guloksuz et al. (2017)^48^ | 1 | 1 | 1 | 0 | 0 | 1 | 1 | 1 | 6 | Medium |
| Hamed et al. (2019)^49^ | 1 | 0 | 1 | 1 | 1 | 1 | 0 | 0 | 5 | Medium |
| Han et al. (2017)^50^ | 0 | 1 | 0 | 0 | 1 | 1 | 1 | 1 | 5 | Medium |
| Han et al. (2021)^51^ | 0 | 1 | 1 | 1 | 0 | 1 | 1 | 1 | 6 | Medium |
| Han et al. (2022)^52^ | 1 | 1 | 0 | 1 | 0 | 1 | 1 | 1 | 6 | Medium |
| Hashim et al. (2013)^53^ | 1 | 1 | 1 | 1 | 0 | 1 | 0 | 1 | 6 | Medium |
| Hu et al. (2018)^54^ | 0 | 1 | 1 | 0 | 1 | 1 | 1 | 1 | 6 | Medium |
| Ibrahim et al. (2015)^55^ | 1 | 1 | 1 | 1 | 0 | 1 | 1 | 1 | 7 | Low |
| Kajizuka et al. (2010)^56^ | 1 | 1 | 1 | 1 | 1 | 1 | 1 | 1 | 8 | Low |
| Karadag et al. (2024)^57^ | 1 | 1 | 1 | 1 | 0 | 1 | 0 | 1 | 6 | Low |
| Kareem et al. (2018)^58^ | 1 | 0 | 0 | 1 | 0 | 1 | 0 | 1 | 4 | High |
| Kartalci et al. (2022)^59^ | 1 | 1 | 1 | 0 | 0 | 1 | 0 | 1 | 5 | Medium |
| Kordulewska et al. (2019)^60^ | 0 | 1 | 1 | 0 | 1 | 1 | 1 | 0 | 5 | Medium |
| Li et al. (2024)^61^ | 1 | 1 | 1 | 1 | 0 | 1 | 1 | 1 | 7 | Low |
| Lochman et al. (2018)^62^ | 0 | 1 | 0 | 0 | 0 | 1 | 0 | 0 | 2 | High |
| Lv et al. (2025)^63^ | 0 | 1 | 1 | 0 | 0 | 0 | 1 | 1 | 4 | High |
| Manzardo et al. (2012)^64^ | 0 | 1 | 0 | 1 | 0 | 1 | 0 | 1 | 4 | High |
| Meguid et al. (2018)^65^ | 0 | 1 | 1 | 1 | 0 | 1 | 1 | 1 | 6 | Medium |
| Mostafa & Al-Ayadhi (2015)^66^ | 0 | 1 | 1 | 1 | 0 | 1 | 0 | 1 | 5 | Medium |
| Napolioni et al. (2013)^67^ | 0 | 1 | 0 | 1 | 0 | 1 | 1 | 1 | 5 | Medium |
| Nie et al. (2023)^68^ | 0 | 1 | 1 | 0 | 0 | 1 | 0 | 1 | 4 | High |
| Ning et al. (2019)^69^ | 1 | 1 | 0 | 1 | 0 | 1 | 1 | 1 | 6 | Medium |
| Noori et al. (2024)^70^ | 0 | 1 | 1 | 1 | 0 | 1 | 0 | 1 | 5 | Medium |
| Nour-Eldine et al. (2024)^71^ | 0 | 1 | 1 | 1 | 0 | 1 | 0 | 1 | 5 | Medium |
| Okada et al. (2007)^72^ | 1 | 1 | 1 | 1 | 1 | 1 | 1 | 1 | 8 | Low |
| Pardo et al. (2017)^73^ | 0 | 1 | 0 | 0 | 0 | 1 | 0 | 1 | 3 | High |
| Pecorelli et al. (2016)^74^ | 0 | 1 | 0 | 0 | 0 | 1 | 0 | 1 | 3 | High |
| Peng et al. (2021)^75^ | 1 | 1 | 1 | 1 | 1 | 1 | 0 | 1 | 7 | Low |
| Ramsey et al. (2013)^76^ | 0 | 1 | 0 | 1 | 0 | 1 | 0 | 0 | 3 | High |
| Rodrigues et al. (2014)^77^ | 0 | 1 | 0 | 1 | 0 | 1 | 0 | 1 | 4 | High |
| Russo et al. (2013)^78^ | 0 | 1 | 0 | 0 | 0 | 1 | 0 | 1 | 3 | High |
| Sallam et al. (2024)^79^ | 1 | 1 | 1 | 1 | 0 | 1 | 0 | 1 | 6 | Medium |
| Saresella et al. (2016)^80^ | 1 | 1 | 0 | 0 | 0 | 1 | 1 | 1 | 5 | Medium |
| Shaker et al. (2016)^81^ | 0 | 1 | 1 | 1 | 0 | 1 | 1 | 1 | 6 | Medium |
| Shen et al. (2016)^82^ | 1 | 1 | 1 | 0 | 1 | 1 | 0 | 1 | 6 | Medium |
| Shen et al. (2021)^83^ | 1 | 1 | 1 | 0 | 1 | 1 | 0 | 1 | 6 | Medium |
| Shmarina et al. (2020)^84^ | 0 | 1 | 0 | 1 | 0 | 1 | 0 | 1 | 4 | High |
| Simsek et al. (2021)^85^ | 1 | 1 | 1 | 1 | 1 | 1 | 1 | 1 | 8 | Low |
| Singh et al. (1991)^86^ | 0 | 1 | 0 | 0 | 0 | 1 | 1 | 1 | 4 | High |
| Singh et al. (1996)^87^ | 1 | 1 | 0 | 0 | 0 | 1 | 1 | 0 | 4 | High |
| Singh et al. (2017)^88^ | 1 | 1 | 0 | 0 | 0 | 1 | 1 | 1 | 5 | Medium |
| Steeb et al. (2014)^89^ | 1 | 1 | 0 | 1 | 0 | 1 | 1 | 0 | 5 | Medium |
| Sugihara et al. (2007)^90^ | 1 | 1 | 1 | 1 | 1 | 1 | 1 | 1 | 8 | Low |
| Suzuki et al. (2011)^91^ | 1 | 0 | 1 | 1 | 1 | 1 | 1 | 1 | 7 | Low |
| Sweeten et al. (2004)^92^ | 1 | 1 | 0 | 1 | 0 | 1 | 1 | 1 | 6 | Medium |
| Tobiasova et al. (2011)^93^ | 0 | 1 | 0 | 0 | 0 | 1 | 1 | 1 | 4 | High |
| Tonhajzerova et al. (2015)^94^ | 1 | 1 | 1 | 1 | 0 | 1 | 1 | 0 | 6 | Medium |
| Tostes et al. (2012)^95^ | 1 | 1 | 0 | 1 | 0 | 1 | 1 | 1 | 6 | Medium |
| Tsilioni et al. (2015)^96^ | 0 | 1 | 1 | 0 | 0 | 1 | 1 | 0 | 4 | High |
| Wang et al. (2025)^97^ | 0 | 1 | 1 | 1 | 0 | 1 | 0 | 1 | 5 | Medium |
| Xie et al. (2017)^98^ | 1 | 1 | 0 | 1 | 0 | 1 | 0 | 1 | 5 | Medium |
| Yan et al. (2015)^99^ | 1 | 1 | 1 | 1 | 1 | 1 | 1 | 1 | 8 | Low |
| Yang et al. (2015) ^100^ | 1 | 1 | 1 | 1 | 1 | 1 | 1 | 1 | 8 | Low |
| Yang et al. (2015) ^101^ | 1 | 1 | 1 | 1 | 1 | 1 | 1 | 1 | 8 | Low |
| Zhang et al. (2023)^102^ | 1 | 1 | 1 | 1 | 0 | 1 | 0 | 1 | 6 | Medium |
| Zurita et al. (2020)^103^ | 0 | 0 | 0 | 1 | 0 | 1 | 0 | 1 | 3 | High |

**Table S7. List of Excluded Studies and Exclusion Reasons**

| **Author(s)** | **Title** | **Exclusion reason** |  |
| --- | --- | --- | --- |
| (a) **Studies excluded during full-text screening after study identification via online databases (n = 52)** | | | |
| Al Olaby et al. (2017)^104^ | Identification of molecular biomarkers predictive of response to targeted treatment in Fragile X syndrome and autism spectrum disorder. | No control group |  |
| Al Olaby et al. (2020)^105^ | Molecular biomarkers predictive of sertraline treatment response in young children with autism spectrum disorder. | No control group |  |
| Al-Ayadhi (2005)^106^ | Pro-inflammatory cytokines in autistic children in central Saudi Arabia. | Co-occurring neurological condition (Rett Syndrome) |  |
| Al-Ayadhi et al. (2018)^107^ | Impact of auditory integrative training on transforming growth factor-beta1 and its effect on behavioral and social emotions in children with autism spectrum disorder. | No control group |  |
| Aldossari et al. (2023) ^108^ | Upregulation of inflammatory mediators in peripheral blood cd40+ cells in children with autism spectrum disorder | Inappropriate methods/outcome measures (PBMC) |  |
| Alekseeva et al. (2024) ^109^ | Expression of key molecules of the classical inflammation activation pathway in blood leukocytes of autistic children | Foreign language publication |  |
| Ansari et al. (2021)^110^ | The effect of water-based intervention on sleep habits and two sleep-related cytokines in children with autism. | No control group |  |
| Ashwood (2018)^111^ | Differential T cell levels of tumor necrosis factor receptor-II in children with autism. | Inappropriate methods/ outcome measures (peripheral blood mononuclear cells [PBMC]) |  |
| Balestrieri et al. (2019)^112^ | Children with autism spectrum disorder and their mothers share abnormal expression of selected endogenous retroviruses families and cytokines. | Inappropriate methods/ outcome measures (PBMC) |  |
| Bashir & Al-Ayadhi (2014)^113^ | Effect of camel milk on thymus and activation-regulated chemokine in autistic children: double-blind study. | No control group |  |
| Bryn et al. (2015)^114^ | Brain derived neurotrophic factor (BDNF) and autism spectrum disorders (ASD) in childhood. | Co-occurring neurological condition (Rett Syndrome) |  |
| Bryn et al. (2017)^115^ | Cytokine profile in autism spectrum disorders in children. | Co-occurring neurological condition (Rett Syndrome) |  |
| Businaro et al. (2016)^116^ | Interleukin-18 modulation in autism spectrum disorders. | Co-occurring neurological condition (epilepsy) |  |
| Cherevko et al. (2024)^117^ | The influence of modulation of intestinal microbiota on clinical and immunological parameters and oxytocin levels in children with autism spectrum disorders | Foreign language publication |  |
| Chez et al. (2007)^118^ | Elevation of tumor necrosis factor-alpha in cerebrospinal fluid of autistic children. | No control group |  |
| Choi et al. (2014)^119^ | Change in plasma cytokine levels during risperidone treatment in children with autism. | No control group |  |
| Chudakova et al. (2024)^120^ | Immune alterations in children with autism spectrum disorders | Foreign language publication |  |
| Dardani et al. (2022)^121^ | Immunological pathways underlying autism: Findings from Mendelian randomization and genetic colocalisation analyses. | Inappropriate methods/ outcome measures (i.e., genetically proxied levels of plasma cytokines) |  |
| Eftekharian et al. (2020)^122^ | Expression analysis of suppressor of cytokine signaling (SOCS) genes in blood of autistic patients. | Inappropriate methods/ outcome measures (No cytokines, only gene expression) |  |
| Ferguson et al. (2016)^123^ | Associations between cytokines, endocrine stress response, and gastrointestinal symptoms in autism spectrum disorder. | No control group |  |
| Filippova et al. (2023)^124^ | Leukocyte cytokine expression is associated with severity of autism in children | Foreign language publication |  |
| Hassan et al. (2018)^125^ | The use of multi-parametric biomarker profiles may increase the accuracy of ASD prediction. | Inappropriate methods/ outcome measures (No cytokine) |  |
| Inoue et al. (2019)^126^ | Dietary supplementation with partially hydrolyzed guar gum helps improve constipation and gut dysbiosis symptoms and behavioral irritability in children with autism spectrum disorder. | No control group |  |
| Jàcome et al. (2016)^127^ | Peripheral inflammatory markers contributing to comorbidities in autism. | Co-occurring neurological condition (epilepsy) |  |
| Javadfar et al. (2020)^128^ | Effects of vitamin D supplementation on core symptoms, serum serotonin, and interleukin-6 in children with autism spectrum disorders: A randomized clinical trial. | No control group |  |
| Kerley et al. (2017)^129^ | Lack of effect of Vitamin D3 supplementation in autism: a 20-week, placebo-controlled RCT. | No control group |  |
| Kilinç et al. (2025)^130^ | What are the levels and interactions of neuroligin-1, neuroligin-3, and inflammatory cytokines (IL-6, IL-8) in children diagnosed with autism spectrum disorder? | Inappropriate methods/ outcome measures |  |
| Kim et al. (2023)^131^ | Neonatal immune signatures differ by sex regardless of neurodevelopmental disorder status: macrophage migration inhibitory factor (MIF) alone reveals a sex by diagnosis interaction effect | No autism group |  |
| Kong et al. (2021)^132^ | Probiotic and oxytocin combination therapy in patients with autism spectrum disorder: a randomized, double-blinded, placebo-controlled pilot trial. | No control group |  |
| Long et al. (2024)^133^ | Interactions between circulating inflammatory factors and autism spectrum disorder: a bidirectional Mendelian randomization study in European population | Inappropriate methods/outcome measures |  |
| Malek et al. (2020)^134^ | Prednisolone as adjunctive treatment to Risperidone in children with regressive type of autism spectrum disorder: a randomized, placebo-controlled trial. | No control group |  |
| Mankad et al. (2015)^135^ | A randomized, placebo controlled trial of omega-3 fatty acids in the treatment of young children with autism. | No control group |  |
| Masi et al. (2017)^136^ | Cytokine levels and associations with symptom severity in male and female children with autism spectrum disorder. | No control group |  |
| Mazahery et al. (2020)^137^ | Inflammation (IL-1 beta) modifies the effect of Vitamin D and Omega-3 long chain polyunsaturated fatty acids on core symptoms of autism spectrum disorder-an exploratory pilot study. | No control group |  |
| Melamed et al. (2018)^138^ | A pilot study of high-dose intravenous immunoglobulin 5% for autism: Impact on autism spectrum and markers of neuroinflammation. | No control group |  |
| Nadeem et al. (2022)^139^ | Imbalance in pro-inflammatory and anti-inflammatory cytokines milieu in B cells of children with autism. | Inappropriate methods/ outcome measures (PBMC, RNA expression, not in peripheral blood) |  |
| Pardo et al. (2013)^140^ | A pilot open-label trial of minocycline in patients with autism and regressive features. | No control group |  |
| Prosperi (2019)^141^ | Inflammatory biomarkers are correlated with some forms of regressive autism spectrum disorder. | No control group |  |
| Raghavan et al. (2018)^142^ | Fetal and infancy growth pattern, cord and early childhood plasma leptin, and development of autism spectrum disorder in the Boston birth cohort. | Inappropriate methods/ outcome measures (blood analysis prior to autism diagnosis) |  |
| Ricci et al. (2013)^143^ | Altered cytokine and BDNF levels in autism spectrum disorder. | Co-occurring neurological condition (epilepsy) |  |
| Riordan et al. (2019)^144^ | Allogeneic human umbilical cord mesenchymal stem cells for the treatment of autism spectrum disorder in children: safety profile and effect on cytokine levels. | No control group |  |
| Russo et al. (2014)^145^ | Decreased mitogen inducible gene 6 (MIG-6) associated with symptom severity in children with autism. | Inappropriate methods/ outcome measures (No cytokine) |  |
| Russo et al. (2009)^146^ | Decreased serum hepatocyte growth factor (HGF) in autistic children with severe gastrointestinal disease. | Co-occurring other condition (Gastrointestinal disease) |  |
| Sanctuary et al. (2019)^147^ | Pilot study of probiotic/colostrum supplementation on gut function in children with autism and gastrointestinal symptoms. | No control group |  |
| Sasayama et al. (2017)^148^ | Negative correlation between serum cytokine levels and cognitive abilities in children with autism spectrum disorder. | No control group |  |
| Schwarz et al. (2011)^149^ | Sex-specific serum biomarker patterns in adults with Asperger's syndrome. | Insufficient statistical information |  |
| Schwarz et al. (2012)^150^ | Identification of a biological signature for schizophrenia in serum. | Insufficient statistical information |  |
| Shim et al. (2024)^151^ | Alterations in plasma cytokine levels in Korean children with autism spectrum disorder. | Insufficient statistical information |  |
| Than et al. (2023)^152^ | Inflammatory mediators drive neuroinflammation in autism spectrum disorder and cerebral palsy. | Inappropriate methods/outcome measures (CSF cytokine) |  |
| Werner et al. (2022)^153^ | Immune marker levels in severe mental disorders: associations with polygenic risk scores of related mental phenotypes and psoriasis. | No autism group |  |
| Yousefi et al. (2021)^154^ | Is serum TGF-beta 1 and TGF-beta 2 levels correlated to children with autism intensity? | Co-occurring neurological condition (epilepsy) |  |
| Zimmerman et al. (2021)^155^ | Randomized controlled trial of sulforaphane and metabolite discovery in children with Autism Spectrum Disorder. | No control group |  |
| (b) **Studies excluded during full-text screening after study identification via other methods i.e., past meta-analyses (n = 2)** | | | |
| Tsilioni et al. (2014)^156^ | Elevated serum neurotensin and CRH levels in children with autistic spectrum disorders and tail-chasing Bull Terriers with a phenotype similar to autism. | Inappropriate methods/ outcome measures (No cytokine) |  |
| Zimmerman et al. (2005)^157^ | Cerebrospinal fluid and serum markers of inflammation in autism. | Co-occurring neurological  Condition (epilepsy) |  |
| (c) **Studies excluded from the meta-analysis the cytokine they investigated are available in too few datasets (< 3) to meta-analyse (n = 9)** | | | |
| Alhakbany et al. (2022)^158^ | CTRP3 as a novel biomarker in the plasma of Saudi children with autism | Not investigated in at least 3 independent studies |  |
| Al-Ayadhi & Mostafa (2011)^159^ | Increased serum osteopontin levels in autistic children: Relation to the disease | Not investigated in at least 3 independent studies |  |
| Emanuele et al. (2010)^160^ | Increased serum levels of high mobility group box 1 protein in patients with autistic disorder | Not investigated in at least 3 independent studies |  |
| Makris et al. (2021)^161^ | Increased serum concentrations of high mobility group box 1 (HMGB1) protein in children with autism spectrum disorder. | Not investigated in at least 3 independent studies |  |
| Nadeem et al. (2020)^162^ | Dysregulation in IL-6 receptors is associated with upregulated IL-17A related signaling in CD4+T cells of children with autism | Not investigated in at least 3 independent studies |  |
| Raeisy et al. (2022)^163^ | C1q/TNF-related protein-1: Potential biomarker for early diagnosis of autism spectrum disorder. | Not investigated in at least 3 independent studies |  |
| Rose & Ashwood (2019)^164^ | Rapid communication: plasma interleukin-35 in children with autism. | Not investigated in at least 3 independent studies |  |
| Russo et al. (2014)^165^ | Increased epidermal growth factor receptor (EGFR) associated with hepatocyte growth factor (HGF) and symptom severity in children with autism spectrum disorders (ASDs) | Not investigated in at least 3 independent studies |  |
| Tsilioni et al. (2020)^166^ | IL-38 inhibits microglial inflammatory mediators and is decreased in amygdala of children with autism spectrum disorder | Not investigated in at least 3 independent studies |  |

**Table S8. Table of Characteristics of Included Studies**

| **Authors (Year)** | **Cytokines** | **ASD (n)** | **CON (n)** | **ASD age, y (m/md**^c^**)** | **ASD male (%)** | **CON age, y (m/md**^c^**)** | **CON male (%)** | **Fraction** | **Meds.** | **Co-occurring cond.** | **“Severe” traits (%)**^e^ | **IQ** | **DSM** |
| --- | --- | --- | --- | --- | --- | --- | --- | --- | --- | --- | --- | --- | --- |
| Abdala et al. (2019)^6^ | IL17 | 24 | 10 | NA | NA | NA | NA | serum | NA | yes | NA | NA | DSM-IV |
| Abd-Allah et al. (2020)^7^ | IL1-beta, IL8, TNF-alpha | 35 | 35 | 4.7 | 68.57 | 5.4 | 54.29 | serum | NA | no | 25.71 | NA | DSM-5 |
| Abdel-Salam et al. (2017)^8^ | MCP1 | 20 | 20 | 5.67 | 75 | 5.67^d^ | 75 | serum | no | NA | NA | NA | DSM-IV |
| Al-ayadhi & Mostafa (2012)^9^ | IL17A | 45 | 40 | 8.44 | 80 | 8.33 | 80 | serum | NA | NA | 62.22 | NA | DSM-IV |
| Al-ayadhi & Mostafa (2013)^10^ | TARC, CCL22/MDC | 56 | 32 | 7.54 | 82.14 | 7.19 | 81.25 | serum | NA | NA | 64 | NA | DSM-IV |
| Al-Bishri et al. (2023)^11^ | IL17A, IL35 | 25 | 25 | NA | NA | NA | NA | serum | NA | no | NA | IQ <70 (100%) | DSM-5 |
| Almashhady et al. (2025)^12^ | TNF-alpha | 50 | 50 | NA | 82 | NA | 82 | serum | NA | no | 16 | NA | NA |
| Alshammary et al. (2023)^13^ | TNF-alpha, IL6 | 50 | 22 | NA | NA | NA | NA | serum | NA | NA | NA | NA | NA |
| Alzghoul et al. (2019)^14^ | IL6, IL8, IL9, IL10, TNF-alpha | 80 | 86 | 6.48 | 83.75 | 6.1 | 47.67 | plasma | NA | NA | NA | NA | DSM-5 |
|  |  | 80 | 51^a^ | 6.48 | 83.75 | 6.43 | 62.75 | plasma | NA | NA | NA | NA | DSM-5 |
| Anastasescu et al. (2024)^15^ | IL6, CXCL8, TNF-alpha | 45 | 30 | NA | 77.77 | NA | 43.33 | serum | no | NA | NA | NA | DSM-5 |
| Anindya et al. (2025)^16^ | IL6, IL1-beta | 98 | 96 | 4.3 | 77.6 | 3.4 | 57.3 | plasma | NA | no | NA | NA | DSM-5 |
| Artık et al. (2023)^17^ | TWEAK | 40 | 40 | 3.83 | 85 | 3.77 | 77.5 | serum | no | NA | NA | NA | DSM-5 |
| Ashaat et al. (2017)^18^ | IL12, IL17 | 60 | 60 | 8.7 | 93.3 | 7.9 | 76.7 | plasma | NA | NA | 23.33 | NA | DSM-IV |
| Ashwood et al. (2008)^19^ | Leptin | 70 | 50 | 4.25^c^ | 87 | 4.33 | 76 | plasma | yes | NA | NA | NA | DSM-IV |
|  |  | 70 | 26^a^ | 4.25^c^ | 87 | 5.1^c^ | 77 | plasma | yes | NA | NA | NA | DSM-IV |
|  |  | 70 | 23^b^ | 4.25^c^ | 87 | 3.38^c^ | 87 | plasma | yes | NA | NA | NA | DSM-IV |
| Ashwood et al. (2008)^20^ | TGF-beta-1 | 75 | 36 | 3.4^c^ | 91 | 3.6^c^ | 67 | plasma | NA | NA | NA | NA | DSM-IV |
|  |  | 75 | 32^a^ | 3.4^c^ | 91 | 3.9^c^ | 88 | plasma | NA | NA | NA | NA | DSM-IV |
| Ashwood et al. (2011)^21^ | GM-CSF, IFN-gamma, IL1-beta, IL2, IL4, IL5, IL6, IL8, IL10, IL12(p40), IL13, and TNF-alpha | 97 | 87 | 3.4^c^ | 86.6 | 3.4^c^ | 81.61 | plasma | no | NA | NA | NA | DSM-IV |
|  |  | 97 | 39^b^ | 3.4^c^ | 86.6 | 3.5^c^ | 71.79 | plasma | no | NA | NA | NA | DSM-IV |
| Ashwood et al. (2011)^22^ | MCP1, MIP1-alpha, MIP1-beta, RANTES, Eotaxin, IP10 | 80 | 58 | 3.6^c^ | 83.75 | 3.6^c^ | 67.25 | plasma | no | NA | NA | NA | DSM-IV |
|  |  | 80 | 37^b^ | 3.6^c^ | 83.75 | 3.5^c^ | 72.97 | plasma | no | NA | NA | NA | DSM-IV |
| Barbosa et al. (2015)^23^ | IL33, sST2, IL1-beta | 30 | 18 | 9.71 | 83.33 | 8.36 | 83.88 | plasma | yes | NA | NA | NA | DSM-IV-TR |
| Basheer et al. (2018)^24^ | IL1-beta, IL6, IL17A, IFN-gamma, IL7 | 50 | 30 | 6.17 | 96 | 6.75 | 90 | serum | no | yes | 44 | NA | DSM-5 |
| Belica et al. (2023)^25^ | IL1-alpha, IL1-beta, IL1-RA, IL2, IL3, IL4, IL5, IL6, IL7, IL8, IL9, IL10, IL12p40, IL12p70, IL13, IL15, IL17, IFN-gamma, IFN-alpha-2, TNF-alpha, TNF-beta, G-CSF, GM-CSF, TGF-alpha, CCL2, CCL3, CCL4, CCL5, CCL7, Eotaxin, CXCL1, CXCL10, CX3CL1. | 40 | 21 | 7.1 | 85 | 7.4 | 52.4 | plasma | NA | NA | 60 | NA | DSM-5 |
| Bilgiç et al. (2024)^26^ | BAFF, APRIL, TWEAK | 65 | 40 | 3 | 80 | 2.8 | 71 | serum | no | NA | NA | NA | DSM-5 |
| Blardi et al. (2010) ^27^ | Leptin, Adiponectin | 35 | 35 | 14.1 | 60 | 14.1 | 60 | plasma | yes | No | NA | NA | DSM-IV |
| Cao et al. (2021) ^28^ | IL1-beta, IL2, IL4, IL5, IL6, IL8, IL10, IL17A, IL17F, TNF-alpha, TNF-beta, IFN-gamma, IL22, IL12p70 | 45 | 41 | 6.8 | 80 | 5.16 | 82.93 | plasma | no | no | 40 | NA | DSM-5 |
| Carissimi et al. (2019)^29^ | TNF-alpha, IL10, TGF-beta, IL1-beta, IL8, IFN-gamma | 30 | 14 | 3^c^ | 96.67 | 10^c^ | 50 | serum | NA | NA | 33.33 | NA | DSM-5 |
| Carpita et al. (2023)^30^ | IL6 | 24 | 24 | 18-65 | NA | NA | NA | plasma | no | NA | NA | NA | DSM-5 |
| Croonenberghs et al. (2002)^31^ | IL6, IL2R, IL1RA | 13 | 13 | 14.5 | 100 | 15.1 | 100 | serum | no | NA | NA | IQ<60 (7.7%), IQ>60 (92.3%) | DSM-IV |
| Denney et al. (1996)^32^ | IL2R | 10 | 10 | 11.2 | 100 | 11.3 | 100 | plasma | yes | NA | NA | NA | DSM-III |
| El Gohary et al. (2015)^33^ | TGF-beta | 30 | 30 | NA | 86.67 | NA | 73.33 | plasma | no | no | 20 | NA | DSM-IV-TR |
| El Wakkad et al. (2006)^34^ | IL1-beta, IL6 | 21 | 21 | 6.97 | 76.2 | 7.81 | 61.9 | plasma | NA | NA | NA | NA | DSM-IV |
| El-Ansary et al. (2011)^35^ | IL6, TNF-alpha | 25 | 16 | NA | 100 | NA | 100 | plasma | NA | no | NA | NA | NA |
| El-Ansary et al. (2012)^36^ | TGF-beta, IFN-gamma | 20 | 19 | NA | 100 | NA | 100 | plasma | NA | no | NA | IQ < 80 (100%) | NA |
| El-Ansary et al. (2014)^37^ | TNF-alpha, IL6, IFN-gamma | 15 | 19 | NA | 100 | NA | 100 | plasma | NA | no | NA | NA | NA |
| El-Ansary et al. (2016)^38^ | IL10, IL12 | 35 | 38 | NA | NA | NA | NA | plasma | NA | no | NA | NA | NA |
| Emanuele et al. (2010)^39^ | IL1-beta, IL6, IL10 | 22 | 28 | 28.1 | 81.82 | 28.7 | 75 | serum | no | NA | NA | Raven's Progressive Matrices =13.5 | DSM-IV |
| Enstrom et al. (2008)^40^ | IL17, IL23 | 40 | 20 | 3.5 | 90 | 3.8 | 80 | plasma | NA | NA | NA | NA | DSM-IV |
| Enstrom et al. (2008)^41^ | BDNF | 37 | 27 | 3.58 | 91 | 3.13 | 67 | plasma | NA | NA | NA | NA | DSM-IV |
|  |  | 37 | 9^a^ | 3.58 | 91 | 3.81 | 56 | plasma | NA | NA | NA | NA | DSM-IV |
|  |  | 37 | 6^b^ | 3.58 | 91 | 2.71 | 67 | plasma | NA | NA | NA | NA | DSM-IV |
| Ferencova et al. (2023)^42^ | IFN-gamma, IL1A, IL1-beta, IL2, IL4, IL6, IL8, IL10, TNF-alpha | 20 | 20 | 12.4 | 75 | 31.2 | 75 | plasma | no | no | NA | IQ>70 (100%) | DSM-5 |
| Filippova et al. (2023)^43^ | IL6, IFN-gamma, TNF-alpha, IL1-beta, IL4, IL10 | 55 | 11 | NA | 78.2 | NA | 81.8 | plasma | NA | NA | 32.7 | NA | DSM-IV |
| Fujita-Shimizo et al. (2010)^44^ | Adiponectin | 31 | 31 | 11.6 | 100 | 12.1 | 100 | serum | no | no | NA | NA | DSM-IV-TR |
| Ghaffari et al. (2016)^45^ | TNF-alpha, Visfatin, Resistin | 30 | 30 | 7.72 | 73.3 | 8.4 | 73.3 | serum | no | NA | NA | NA | DSM-IV-TR |
| Gomez-Fernandez et al. (2018)^46^ | IL1-beta, IL6, IL8, MCP1, MCP2, TNF-alpha, HGF, RANTES, BDNF, NGF | 54 | 54 | 3.64 | 83 | 4.07 | 84 | plasma | no | NA | NA | NA | DSM-5 |
| Grigorenko et al. (2008)^47^ | MIF | 10 | 10^a^ | NA | NA | NA | NA | plasma | NA | NA | NA | NA | NA |
| Guloksuz et al. (2017)^48^ | IL1-beta, IL4, IL6, IL10, IL17A, TNF-alpha, IFN-gamma | 40 | 35 | 7.13 | 75 | 6.75 | 63 | plasma | no | NA | 57.5 | NA | DSM-IV |
| Hamed et al. (2019)^49^ | TGF-beta-2 | 34 | 29 | NA | 100 | NA | 100 | plasma | NA | no | NA | NA | DSM-5 |
| Han et al. (2017)^50^ | TGF-beta-1, MIF, CCL2, CCL5, CXCL8, CXCL9, CXCL12 | 22 | 13 | 9.5 | 90.91 | 10.92 | 69.23 | serum | no | yes | NA | Mean(SD) IQ = 99.4 (8.29) | DSM-5 |
| Han et al. (2021)^51^ | IL1-beta, IL2R, IL6, IL8 | 50 | 110 | 7.96 | 80 | 8.61 | 83.6 | serum | no | NA | NA | NA | DSM-IV |
| Han et al. (2022)^52^ | CCL2, CCL5, BDNF | 18 | 15 | 10.28 | 61.5 | 11.36 | 38.5 | serum | no | NA | NA | Mean(SD) IQ = 102.6 (7.82) | DSM-5 |
| Hashim et al. (2013)^53^ | TGF-beta-1, IL23, IL19 | 50 | 50 | 8.7 | 54 | 9.1 | 60 | plasma | NA | NA | 38 | NA | DSM-IV-TR |
|  |  | 50 | 50^a^ | 8.7 | 54 | 8.6 | 56 | plasma | NA | NA | 38 | NA | DSM-IV-TR |
| Hu et al. (2018)^54^ | Eotaxin, Fraktaline, IFN-gamma, IL1RA, IL8, MCP1, MIP1-alpha, MIP1-beta, TGF-beta-1, TNF-alpha, VEGF-A | 41 | 87 | 3^c^ | 82.76 | 4.58^c^ | 68.29 | plasma | no | no | NA | NA | DSM-5 |
| Ibrahim et al. (2015)^55^ | IL12 | 20 | 20 | 5.73 | 80 | 6.75 | 60 | serum | no | NA | NA | NA | DSM-IV-TR |
| Kajizuka et al. (2010)^56^ | PDGF-AA, PDGF-AB, PDGF-BB, VEGF | 31 | 31 | 12.3 | 100 | 12.4 | 100 | serum | no | no | NA | Mean(SD) IQ = 89.1 (22.6) | DSM-IV-TR |
| Karadag et al. (2024)^57^ | TGF-beta | 41 | 41 | 7.2 | 85.4 | 7.8 | 73.2 | serum | no | no | NA | NA | DSM-5 |
| Kareem et al. (2018)^58^ | IL6 | 30 | 30 | NA | 83.33 | NA | 83.33 | serum | NA | NA | NA | NA | NA |
| Kartalci et al. (2022)^59^ | IL6, TNF-alpha, TGF-beta | 35 | 35 | 8^c^ | 80 | 9^c^ | 68.6 | serum | yes | NA | NA | NA | DSM-5 |
| Kordulewska et al. (2019)^60^ | IL1-beta, IL4, IL6, IL8, IL10, IL13, TNF-alpha, IFN-gamma | 42 | 20 | 6 | 88.1 | 7.35 | 65 | serum | no | no | NA | IQ<70 (21.4%) | DSM-5 |
| Li et al. (2024)^61^ | IL17A | 50 | 50 | 4.25 | 54 | 4.19 | 62 | serum | no | NA | 46 | NA | DSM-5 |
| Lochman et al. (2018)^62^ | BDNF, MCP1, TARC, IFN-gamma, TGF-beta, IL6, IL10, TNF-alpha | 26 | 11^a^ | 4.69 | 80.77 | 7.38 | 18.18 | serum | NA | NA | 46.15 | NA | DSM-IV |
| Lv et al. (2025)^63^ | IL8, VEGFA, CD8A, MCP3, GDNF, CDCP1, CD244, IL7, OPG, LAP-TGF-beta-1, uPA, IL6, IL17C, MCP1, IL17A, CXCL11, AXIN1, TRAIL, IL20RA, CXCL9, CST5, IL2RB, IL1-alpha, OSM, IL2, CXCL1, TSLP, CCL4, CD6, SCF, IL18, SLAMF1, TGF-alpha, MCP4, CCL11, TNFSF14, FGF23, IL10RA, FGF5, MMP1, LIFR, FGF21, CCL19, IL15RA, IL10RB, IL22-RA1, I-18R1, PDL1, BNGF, CXCL5, TRANCE, HGF, IL12-beta, IL24, IL13, ARTN, MMP-10, IL10, TNF, CCL23, CD5, CCL3, Flt3L, CXCL6, CXCL10, 4E-BP1, IL20, SIRT2, CCL28, DNER, EN-RAGE, CD40, IL33, IFN-gamma, FGF-19, IL4, LIF, NRTN, MCP-2, CASP-8, CCL25, CX3CL1, TNFRSF9, NT-3, TWEAK, CCL20, ST1A1, STAMBP, IL5, ADA, TNF-beta, CSF-1 | 60 | 28 | 5 | 78.3 | 6.5 | 64.3 | plasma | no | NA | NA | NA | DSM-5 |
| Manzardo et al. (2012)^64^ | IL1-alpha, IL1-beta, IL1RA, IL2, IL2RA, IL3, IL4, IL5, IL6, IL7, IL9, IL10, IL12p40, IL12p70, IL13, IL15, IL-17, CD40L, Flt3 ligand, G-CSF, GM-CSF, EGF, Eotaxin, FGF-2, Fraktaline, GRO, IL8, IP10, MCP1, MCP3, MSC, MIP1-alpha, MIP1-beta, TGF-alpha, VEGF, TNF-alpha, TNF-beta, IFN-alpha-2, IFN-gamma | 104 | 48 | 7.77 | 74.75 | 7.94 (males); 7.25 (females) | 74.75 | plasma | NA | NA | NA | NA | DSM-IV-TR |
| Meguid et al. (2018)^65^ | TNF-alpha | 90 | 30 | 5.11 (atypical); 6.1 (childhood) | 73.33 | 6.6 | 51 | plasma | no | NA | NA | NA | DSM-IV |
| Mostafa & Al-Ayadhi (2015)^66^ | CXCL5 | 62 | 62 | 6.9 | 77.4 | 7.1 | 75.8 | serum | NA | no | NA | IQ>70 (45.16%); 50-69 (32.26%); 35-49 (22.58%) | DSM-IV |
|  |  |  |  |  |  |  |  |  |  |  |  |  |  |
| Napolioni et al. (2013)^67^ | CXCL13, Eotaxin, Eotaxin-2, G-CSF, GM-CSF, I-309, IFN-gamma, IL1-alpha, IL1-beta, IL1RA, IL2, IL4, IL5, IL6, IL6sR, IL7, IL8, IL10, IL11, IL12p40, IL12p70, IL13, IL15, IL16, IL17, MCP1, M-CSF, MIG, MIP1-alpha, MIP1-beta, MIP1-d, PDGF-BB, RANTES, TIMP1, TIMP2, TNF-alpha, TNF-beta, TNF-sRI, TNF-sRII | 25 | 25^a^ | 8.11 | 100 | 7.44 | 100 | plasma | no | NA | NA | Median(IQR) IQ=71 (59-83) | DSM-IV |
| Nie et al. (2023)^68^ | IFN-gamma,TNF-alpha, IL2, IL4, IL5, IL6, IL10, TGF-beta, IL17A | 82 | 50 | 6.12 | 81.7 | 5.24 | 88 | plasma | no | no | 61 | NA | DSM-IV |
| Ning et al. (2019)^69^ | MIF, IL6 | 102 | 102 | 4.5 | 78.43 | 4.5 | 78.43 | serum | no | NA | 66.67 | NA | DSM-5 |
| Noori et al. (2024)^70^ | TNF-alpha, TGF-beta, IL1-beta, IL10, 1L8, IL6 | 22 | 12 | 5.41 | 81.8 | 5.42 | 83.3 | serum | no | no | NA | NA | DSM-5 |
| Nour-Eldine et al. (2024)^71^ | BDNF, FGF2, HGF, IL1-alpha, IL2, IL6, IL9, IL13, IL18, IL23, IP10, MIP1-alpha, PDGF-BB, EGF, GM-CSF, IFN alpha, IL1-beta, IL4, IL7, IL10, IL15, IL21, IL27, LIF, MIP1-beta, PIGF-1, Eotaxin, GRO-alpha, IFN-gamma, IL1RA, IL5, IL8, IL12p70, IL17A, IL22, IL31, MCP1, BNGF, RANTES, SCF, TNF-beta, SDF1-alpha, VEGF-A, TNF-alpha, and VEGF-D | 100 | 60 | 3.37 | 79 | 3.56 | 80 | plasma | no | NA | 51 | NA | DSM-5 |
| Okada et al. (2007)^72^ | TGF-beta-1 | 19 | 21 | 22.7 | 100 | 23.4 | 100 | serum | no | no | NA | Mean(SD) IQ=97.7 (20.7) | DSM-IV |
| Pardo et al. (2017)^73^ | IL1-alpha, IL1-RA, IL1-beta, IL2, IL2RA, IL3, IL4, IL5, IL6, IL7, IL9, IL10, IL12p40, IL12p70, IL13, IL15, IL17, IFN-alpha-2, IFN-gamma, TNF-alpha, TNF-beta, TGF-alpha, EGF, G-CSF, GM-CSF, VEGF, FGF2, FLT3L, sCD40L,CCL2/MCP1, CCL3/MIP1-alpha, CCL4/MIP1-beta, CCL7/MCP3, CCL11/Eotaxin, CCL22/MDC, CXCL1/GRO-alpha, CXCL8/IL8, CXCL10/IP10, CX3CL1/Fractalkine | 75 | 34 | 3- | NA | 3 (age group 1); 5 (age group 2); 7 (age group 3) | NA | serum | NA | NA | NA | NA | DSM-IV-TR |
| Pecorelli et al. (2016)^74^ | IL8, IL13, IL1-beta, IL1RA, IL2, IL4, IL5, IL6, IL7, IL8, IL9, IL10, IL12p70, IL13, IL15, IL17, basic FGF, Eotaxin, G-CSF, GM-CSF, IFN-gamma, IP10, MCP1, MIP1-alpha, MIP1-beta, PDGF-BB, RANTES, TNF-alpha, VEGF | 12 | 8 | 17.7 | NA | 15.4 | NA | serum | NA | NA | NA | NA | DSM-5 |
| Peng et al. (2021)^75^ | MCP1, MIP1-alpha, MIP1-beta, RANTES, IL8, IP10, MIG | 62 | 60 | 3.82 | 83.87 | 3.9 | 71.67 | serum | NA | no | NA | NA | DSM-5 |
| Ramsey et al. (2013)^76^ | IL3, IL12p40, TNF-beta, IL13, MDC, SCF | 37 | 37^a^ | 10.8 | 89.2 | 10.5 | 89.2 | serum | NA | NA | NA | NA | DSM-IV-TR |
| Rodrigues et al. (2014)^77^ | Adiponectin, Leptin, Resistin | 30 | 19 | NA | NA | NA | NA | plasma | yes | NA | NA | NA | DSM-IV-TR |
| Russo et al. (2013)^78^ | EGF | 49 | 40 | 10.6 | 84.21 | 9.5 | 90 | plasma | NA | NA | NA | NA | DSM-IV |
| Sallam et al. (2024)^79^ | IL17A, IL22 | 24 | 24 | 5.25 | 75 | 6 | 65 | serum | NA | no | 20.8 | Mean(SD) IQ=61.58 (9.30) | DSM-5 |
| Saresella et al. (2016)^80^ | IL1-beta | 25 | 30 | 7^c^ | 84 | 7^c^ | 66.67 | serum | no | NA | NA | NA | DSM-5 |
|  |  | 25 | 23^a^ | 7^c^ | 84 | 9^c^ | 52.17 | serum | no | NA | NA | NA | DSM-5 |
| Shaker et al. (2016)^81^ | IL6 | 30 | 22 | 4^c^ | 90 | 4^c^ | 90.9 | serum | no | NA | NA | NA | ICD-10 |
| Shen et al. (2016)^82^ | RANTES, Eotaxin, MIP1-alpha, MIP1-beta, MCP1, IP10, MIG | 42 | 34 | 4.29 | 90.48 | 4.37 | 54.28 | plasma | NA | no | NA | NA | DSM-IV-TR |
| Shen et al. (2021)^83^ | Eotaxin, GM-CSF, IFN-alpha, IFN-gamma, IL10, IL12p40/p70, IL13, IL15, IL17, IL1-beta, ILRA, IL2, IL2R, IL4, IL5, IL6, IL7, IL8, IP10, MCP1, MIG, MIP1-alpha, MIP1-beta, RANTES, TNF-alpha | 45 | 38 | 4.51 | 91.1 | 4.06 | 55.3 | plasma | NA | no | NA | NA | DSM-IV-TR |
| Shmarina et al. (2020)^84^ | IL1-beta, TNF-alpha, IL8, IL17A, IL10, IFN-gamma | 133 | 27 | NA | NA | NA | NA | plasma | NA | NA | 53.38 | NA | DSM-5 |
| Simsek et al. (2021)^85^ | VEGF, IGF1, HIF-alpha | 40 | 40 | 6.98 | 92.5 | 7.79 | 92.5 | serum | no | no | NA | NA | DSM-5 |
| Singh et al. (1991)^86^ | sIL2, sIL2R, sIL1 | 23 | 33 | NA | NA | NA | NA | serum | no | NA | NA | NA | DSM-III |
|  |  | 23 | 13^b^ | NA | NA | NA | NA | serum | no | NA | NA | NA | DSM-III |
| Singh et al. (1996)^87^ | IL12, IFN-gamma, IFN-alpha, IL6, TNF-alpha | 20 | 20 | 10.7 | 80 | 10.4 | 65 | plasma | no | NA | NA | NA | DSM-III |
| Singh et al. (2017)^88^ | IL8, MCP4, MIG, SCF | 36 | 35 | NA | 100 | NA | 100 | serum | no | NA | NA | NA | DSM-IV |
| Steeb et al. (2014)^89^ | IL5, CD40, IL7, TNF-alpha, BDNF, EGF, Eotaxin-3 | 30 | 29 | 32.1 | 46.67 | 32.7 | 44.83 | serum | no | NA | NA | NA | DSM-IV |
| Sugihara et al. (2007)^90^ | HGF | 17 | 18 | 23.1 | 100 | 23 | 100 | serum | no | no | NA | Mean(SD) IQ=98.9 (18.9) | DSM-IV |
| Suzuki et al. (2011)^91^ | IL1-beta, IL1RA, IL2, IL4, IL5, IL6, IL7, IL8, IL9, IL10, IL12p70, IL13, IL15, IL17, Eotaxin, FGF, G-CSF, IFN-gamma, IP10, MCP1, MIP1-alpha, MIP1-beta, PDGF-BB, RANTES, TNF-alpha, VEGF, CTACK, GRO-alpha, HGF, IFN-alpha-2, IL1-alpha, IL2RA, IL3, IL12p40, IL16, IL18, LIF, MCP-3, M-CSF, MIF, MIG, BNGF, SCF, SCGF-beta, SDF1-alpha-M TNF-beta, TRAIL | 28 | 28 | 12.1 | 100 | 12.3 | 100 | plasma | no | no | NA | Mean(SD) IQ=99.6 (18.6) | DSM-IV-TR |
| Sweeten et al. (2004)^92^ | IFN-gamma, TNF-alpha, IL1-beta | 29 | 27 | 6.1 | 86.21 | 6.5 | 85.19 | plasma | no | NA | NA | NA | DSM-IV |
| Tobiasova et al. (2011)^93^ | IL5, IL6, IL10, IL13, IL17, IFN-gamma, IL2, IL4, CD40L, TNF-alpha, IL12p70, IL1-alpha, IL-1RA, IL1-beta, TPO, ENA78, MCP1, MIP1-alpha, MIP1-beta, RANTES, Eotaxin, IP10, ITAC, IL8, EGF, G-CSF, GM-CSF, HGF, FGF, VEGF, Leptin | 98 | 19 | 9.6 | 85 | 13.7 | 63 | serum | no | NA | NA | IQ < 70 (22%) | DSM-IV |
| Tonhajzerova et al. (2015)^94^ | TNF-alpha, IL1-beta, IL8 | 15 | 20 | 9.3 | 86.67 | 9.6 | 86.67 | plasma | no | NA | NA | NA | DSM-5 |
| Tostes et al. (2012)^95^ | IL1-beta, IFN-gamma, TNF-alpha, IL10, IL6, IL4, IL2 | 24 | 24 | 7.4 | 75 | 7.2 | 75 | plasma | no | NA | NA | NA | DSM-IV |
| Tsilioni et al. (2015)^96^ | IL6, IL9, IL31, IL33, TNF-alpha | 40 | 15 | NA | 85 | NA | NA | serum | no | NA | NA | NA | DSM-IV-TR |
| Wang et al. (2025)^97^ | Adiponectin, Leptin, Resistin, Adipsin, Lipocalin-2 | 36 | 18 | 6.4 | 67 | 6.8 | 67 | plasma | no | NA | NA | NA | DSM-5 |
| Xie et al. (2017)^98^ | IL17F, IFN-gamma, IL10, IL17A, IL22, IL1-beta, IL2, IL21, IL4, IL6, TNF-alpha | 32 | 28 | 5.51 | 90.63 | 6.11 | 78.57 | serum | NA | NA | NA | NA | DSM-IV-TR |
| Yan et al. (2015)^99^ | IL6 | 75 | 75 | 3.69 | 78.7 | 3.69 | 78.7 | serum | no | no | NA | NA | DSM-IV |
| Yang et al. (2015) ^100^ | IL6 | 33 | 31 | 12.21 | 81.8 | 12.52 | 77.4 | plasma | no | no | NA | NA | DSM-IV-TR |
| Yang et al. (2015) ^101^ | IL6, TNF-alpha | 35 | 32 | 10.63 | 80 | 11.19 | 78 | plasma | no | no | NA | NA | DSM-IV-TR |
| Zhang et al. (2023)^102^ | IFN-gamma, IL8, IL10, IL17A, TNF-alpha | 105 | 105 | 4 | 84 | 4 | 84 | plasma | NA | NA | NA | NA | DSM-5 |
| Zurita et al. (2020)^103^ | IL6, IFN-gamma, TGF-beta-1, MCP1 | 23 | 34 | 8.9 | 96 | 8.5 | 91.4 | serum | NA | NA | NA | NA | NA |

Abbreviation**.** ASD = autism spectrum disorder, CON = control – unless specified control participants are typically developing individuals unrelated to the autistic participants, n = sample size, % = percentage, m = mean, md = median, other cond. = other conditions, Meds = medication, NA = not available. ^a^ control = typically developing siblings, b control = individuals with delayed development, ^c^ median was used instead of mean as a measure of central tendency, ^d^age-matched controls, ^e^percentage of individuals in the sample classified as having “severe” autism traits according to standardised measures. BDNF = brain-derived neurotrophic factor,  CCL = chemokine ligand, CXCL = C-X-C motif ligand, MCP = monocyte chemoattractant protein, MIP = macrophage inflammatory protein, RANTES = regulated upon activation, normal T cell expressed and presumably secreted , MDC = macrophage-derived chemokine, GRO = growth-regulated oncogene, E/FGF= epidermal/fibroglast growth factor, G-/GM-CSF = granulocyte-/granulocyte-macrophage colony stimulating factor, HGF = hepatocyte growth factor, IFN = interferon, IP10 = interferon gamma-induced protein 10, MIF = macrophage migration inhibitory factor, MIG = monokine induced by gamma interferon, PD/VE/TGF= platelet-derived/vascular endothelial/transforming growth factor, TNF = tumour necrosis factor, MIF = macrophage migration inhibitory factor.

**Figure S1. Forest Plots of Significant Meta-analytic Findings Using All Available Data**

Table S9. Meta-analytic Findings after Removal of Studies with High Risk of Bias.

| Cytokines | Datasets | Autism (n) | Controls (n) | Hedge’s *g* | 95%CI | *p* | Cochran's Q | *p* | *I*^2^ (%) |  |
| --- | --- | --- | --- | --- | --- | --- | --- | --- | --- | --- |
| **Interleukins** | | | | | | | | | | |
| IL1-alpha | 4 | 173 | 133 | 0.149 | [-0.49, 0.78] | .645 | 22.7 | < .0001*** | 84.6 |  |
| **IL1-beta** | **20** | **824** | **731** | **0.633** | **[0.37, 0.90]** | < .0001** | **113.5** | **< .0001***** | **83.4** |  |
| IL2 | 9 | 485 | 362 | 0.219 | [0.02, 0.42] | .034 | 15.2 | .055 | 48.8 |  |
| **IL4** | **13** | **645** | **475** | **0.265** | **[0.07, 0.46]** | .006* | **23.7** | **.014*** | **53.3** |  |
| IL5 | 8 | 467 | 347 | 0.206 | [-0.08, 0.49] | .152 | 23.9 | .001** | 73.0 |  |
| IL6 | 29 | 1227 | 1087 | 0.358 | [0.05, 0.66] | .022 | 187.8 | < .0001*** | 91.8 |  |
| **IL7** | **5** | 228 | 180 | **0.307** | **[0.08, 0.53]** | .008* | **5.22** | **.265** | **19.2** |  |
| **IL8** | **19** | **912** | **809** | **0.274** | **[0.06, 0.48]** | .010* | **66.6** | **< .0001***** | **76.0** |  |
| IL10 | 15 | 716 | 530 | -0.152 | [-0.66, 0.36] | .558 | 132.4 | < .0001*** | 94.4 |  |
| IL12/IL12p40 | 7 | 347 | 275 | 1.013 | [0.16, 1.87] | .020 | 80.6 | < .0001*** | 95.6 |  |
| IL12p70 | 5 | 258 | 214 | 0.701 | [-0.12, 1.53] | .096 | 68.8 | < .0001*** | 94.1 |  |
| IL13 | 7 | 487 | 278 | 0.268 | [-0.02, 0.55] | .067 | 16.9 | .010* | 68.7 |  |
| IL15 | 3 | 170 | 123 | -0.056 | [-0.41, 0.30] | .757 | 4.09 | .129 | 51.1 |  |
| IL17 | 16 | 753 | 611 | 1.491 | [-0.39, 3.38] | .121 | 260.1 | .0001*** | 99.6 |  |
| IL18 | 4 | 178 | 141 | 0.354 | [-0.03, 0.74] | .072 | 7.63 | .054 | 60.9 |  |
| IL22 | 4 | 201 | 153 | 1.057 | [-1.18, 3.30] | .355 | 79.7 | < .0001*** | 98.8 |  |
| IL23 | 3 | 200 | 160 | -0.127 | [-0.74, 0.48] | .683 | 17.1 | .0002*** | 87.6 |  |
| **Interferons** | | | | | | | | | | |
| IFN-alpha | 3 | 173 | 126 | -0.072 | [-0.30, 0.16] | .540 | 0.793 | .673 | 0 |  |
| **IFN-gamma** | **17** | **768** | **615** | **0.769** | **[0.17, 1.36]** | .011* | **134.2** | **< .0001***** | **96.2** |  |
| **Tumour necrosis factors** | | | | | | | | | | |
| TNF-alpha | 25 | 1114 | 876 | 0.159 | [-0.14, 0.46] | .294 | 169.3 | < .0001*** | 89.9 |  |
| TNF-beta | 4 | 198 | 154 | 0.353 | [-0.18, 0.88] | .191 | 15.3 | .002** | 81.7 |  |
| **Colony stimulating factors** | | | | | | | | | | |
| GM-CSF | 5 | 364 | 249 | 0.102 | [-0.12, 0.33] | .374 | 6.80 | .147 | 43.3 |  |
| **Chemokines** | | | | | | | | | | |
| CCL2/MCP1 | 11 | 453 | 432 | 2.7374 | [-0.84, 6.31] | .133 | 331.1 | < .0001*** | 99.8 |  |
| CCL3/MIP1-alpha | 7 | 339 | 332 | -2.408 | [-7.72, 2.90] | .374 | 267.8 | < .0001*** | 99.8 |  |
| CCL4/MIP1-beta | 7 | 343 | 332 | -0.197 | [-2.05, 1.65] | .834 | 276.9 | < .0001*** | 99.1 |  |
| CCL5/RANTES | 9 | 391 | 325 | 0.287 | [-0.08, 0.65] | .123 | 40.3 | < .0001*** | 81.7 |  |
| CXCL10/IP10 | 6 | 298 | 230 | -1.200 | [-2.18, -0.22] | .017 | 104 | < .0001*** | 95.8 |  |
| Eotaxin1 | 6 | 279 | 271 | 0.115 | [-0.31, 0.54] | .595 | 36.8 | < .0001*** | 82.4 |  |
| **Growth Factors** | | | | | | | | | | |
| BDNF | 4 | 202 | 158 | -0.079 | [-0.61, 0.46] | .774 | 12.5 | .006** | 82.1 |  |
| HGF | 4 | 199 | 160 | -0.275 | [-1.18, 0.63] | .553 | 31.4 | < .0001*** | 93.6 |  |
| PDGF-BB | 4 | 184 | 144 | 0.222 | [-0.13, 0.57] | .214 | 6.75 | .080 | 55.3 |  |
| TGF-beta | 11 | 364 | 387 | -0.095 | [-0.81, 0.62] | .793 | 97.1 | < .0001*** | 95.3 |  |
| VEGF-A | 5 | 240 | 246 | 0.074 | [-0.30, 0.44] | .695 | 16 | .003** | 73.8 |  |
| **Adipokines** | | | | | | | | | | |
| Adiponectin | 3 | 102 | 84 | -0.208 | [-0.73, 0.32] | .438 | 6.28 | .043* | 67.8 |  |
| **Other cytokines** | | | | | | | | | | |
| **IL1-RA** | **6** | **252** | **251** | **0.324** | **[0.04, 0.61]** | .024* | **10.39** | **.065** | **53.7** |  |
| MIF | 3 | 152 | 143 | 0.490 | [-0.01, 0.99] | .055 | 7.11 | .029 | 68.6 |  |
| Summary group differences of cytokine peripheral blood concentration between autistic people and non-autistic controls in the meta-analyses after removing studies with a high risk of bias, reported alongside 95% confidence interval (CI), and finding heterogeneity indices (Cochran *Q* and *I*^2^). Bold-printed rows indicate statistically significant group differences. ^a^Cytokines of which concentration difference in the autistic and the non-autistic groups were significant in the meta-analyses involving all studies and remained significant after removing studies with a high risk of bias. ^b^Cytokines of which concentration difference in the autistic and the non-autistic groups became statistically significant in the meta-analysis after removing studies with a high risk of bias. Abbreviations. IL = interleukin, IFN = interferon, TNF = tumour necrosis factor, G-/GM-CSF = granulocyte-/granulocyte-macrophage colony stimulating factor, CCL = chemokine ligand, CXCL = C-X-C motif ligand, MCP = monocyte chemoattractant protein, MIP = macrophage inflammatory protein, NA= not applicable, RANTES = regulated upon activation, normal T cell expressed and presumably secreted, MDC = macrophage-derived chemokine, GRO = growth-regulated oncogene, MIG = monokine induced by gamma interferon, IP10 = interferon γ-induced protein 10, BDNF = brain-derived neurotrophic factor  E/F/H/PD/T/VEGF= epidermal/fibroglast/ hepatocyte/platelet-derived/transforming/vascular endothelial growth factor, RA = receptor agonist, MIF = macrophage migration inhibitory factor. Original *p*-values are reported in the Table, but the asterisks indicated significant thresholds after FDR-correction, * *p*_FDR_ <.05, ** *p*_FDR_ <.01 and *** *p*_FDR_<.001 | | | | | | | | | |  |

**Figure S2. Forest Plots of Significant Meta-analytic Findings After Removal of Studies with High Risk of Bias**

| Cytokines | Datasets  Total (with NSUE) | Autism (n) | Controls (n) | Hedge’s *g* | 95%CI | *p* (* indicated *p*_FDR_) | Cochran's Q | *p* | *I*^2^ (%) |
| --- | --- | --- | --- | --- | --- | --- | --- | --- | --- |
| **IL1-beta** | **31 (3)** | **1400** | **1028** | **0.555** | **[0.28, 0.83]** | **.0001**** | **238.6** | **< .0001***** | **89.3** |
| **IL4** | **20 (1)** | **1073** | **675** | **0.232** | **[0.06, 0.40]** | **.006*** | **47.4** | **.0003***** | **60.5** |
| **IL6** | **45 (1)** | **2047** | **1635** | **0.358** | **[0.11, 0.60]** | **.004*** | **309.9** | **< .0001***** | **91.7** |
| **IL8** | **32 (1)** | **1620** | **1195** | **0.370** | **[0.14, 0.59]** | **.001**** | **191.6** | **< .0001***** | **87.0** |
| IL9 | 9 (3) | 505 | 332 | -0.020 | [-0.18, 0.14] | .811 | 5.51 | .702 | 0.17 |
| IL10 | 28 (3) | 1481 | 920 | -0.220 | [-0.72, 0.28] | .386 | 382.2 | < .0001*** | 96.8 |
| IFN-alpha | 9 (1) | 500 | 299 | 0.040 | [-0.12, 0.20] | .619 | 9.80 | .279 | 8.4 |
| **IFN-gamma** | **33 (2)** | **1660** | **1030** | **0.359** | **[0.08, 0.64]** | **.011*** | **174.8** | **< .0001***** | **90.8** |
| **TNF-alpha** | **45 (3)** | **2106** | **1465** | **0.265** | **[0.07, 0.46]** | **.006*** | **246.6** | **<.0001***** | **85.2** |
| CCL2/MCP1 | 22(1) | 1036 | 733 | 1.322 | [-0.26, 2.91] | .102 | 328.8 | <.0001*** | 99.6 |
| CXCL10/IP10 | 15(1) | 756 | 483 | -0.435 | [-0.93, 0.06] | .087 | 180.8 | <.0001*** | 93.8 |
| The summary group differences in cytokine peripheral blood concentration between autistic people and non-autistic controls in the meta-analyses adjusting for non-significant unreported effects (NSUE). Total number of datasets included in each meta-analysis included some studies providing NSUE (reported within brackets). Standardized mean difference (Hedge’s *g*) are reported alongside 95% confidence interval (CI), and finding heterogeneity indices (Cochran *Q* and *I*^2^). Bold-printed rows indicate statistically significant group differences, after adjusting the *p*-values for false discovery rates (FDR) using the Benjamini-Hochberg method. Original *p*-values reported, asterisks indicated significant thresholds after FDR-correction, * *p*_FDR_ <.05, ** *p*_FDR_ <.01 and *** *p*_FDR_<.001. Abbreviations. IL = interleukin, IFN = interferon, TNF = tumour necrosis factor, CCL = chemokine ligand, CXCL = C-X-C motif ligand, MCP = monocyte chemoattractant protein, IP10 = interferon γ-induced protein 10. | | | | | | | | | |

**Table S10.** Meta-analytic Findings including Studies Providing Non-Significant Unreported Effects (NSUE).

**Table S11. Publications Examining the Association between Cytokine Levels and Autistic Traits**

| **Cytokine** | **Authors (Year)** | **Serum/**  **plasma** | **Association findings** | **Trait measures** |
| --- | --- | --- | --- | --- |
| IL1-alpha | Napolioni et al. (2013)^67^ | plasma | There was no correlation with autism traits (Spearman’s rho=0.476; p=0.054) | SRS total score |
| IL1-beta | Abd-Allah et al. (2020)^7^ | serum | The groups with ‘mild’ (65.6 ± 37.5), ‘moderate’ (51.3 ± 10.8) and ‘severe’ traits (46.8 ± 8.2) did not differ (p-value is not reported). | CARS total score |
| IL1-beta | Anindya et al. (2025)^16^ | plasma | There was a positive correlation with autism traits (Spearman’s rho=0.30, p<0.01), indexed by the BAMBI, and a negative correlation with tactile sensitivity (rho=-0.31, p<0.01), indexed by the SSP. | BAMBI, SSP |
| IL1-beta | Ashwood et al. (2011)^21^ | plasma | There were no correlations with autism traits, although the author described a 'trend' association with social interactions (t = 2.15, p = 0.09). | ADI-R domain score |
| IL1-beta | Ashwood et al. (2011)^21^ | plasma | There was a positive association with stereotypy (t = 3.07, *p* = 0.01), indexed by the ABC domain score, and a non-significant relationship (described as ‘trend’ by the author) with social interactions difficulties (t = 2.15, *p* = 0.09), indexed by the ADI-R domain score. | ABC^(b)^ domain score, ADI-R domain score, and ADOS module 1 domain score |
| IL1-beta | Barbosa et al. (2015)^23^ | plasma | There was no correlation with autism traits (no statistics were reported). | SRS total score |
| IL1-beta | Basheer et al. (2018)^24^ | serum | There was no correlation with autism traits (no statistics were reported). | CARS total score |
| IL1-beta | Cao et al. (2021) ^28^ | plasma | There was no correlation with autism traits (no statistics were reported). | CARS total score |
| IL1-beta | Carissimi et al. (2019)^29^ | serum | There were no correlations with autism traits (no statistics were reported). | ADOS-2 raw score |
| IL1-beta | Emanuele et al. (2010)^39^ | serum | There was no correlation with autism traits (no statistics were reported). All autistic adults are in the category of ‘severe’ (as defined by CARS>40), | ADI-R total score |
| IL1-beta | Filippova et al. (2023)^43^ | plasma | The ‘mild/moderate’ group (median=3.20 IQR[2.54, 4.32]) had higher cytokine concentration than the ‘severe’ (1.86 [1.62, 2.09]) trait group (*р* < 0.05). | CARS total score |
| IL1-beta | Gomez-Fernandez et al. (2018)^46^ | plasma | There were no correlations with autism traits (no statistics were reported). | ADOS raw score, PDDBI |
| IL1-beta | Guloksuz et al. (2017)^48^ | plasma | The groups with ‘mild/moderate’ and ‘severe’ traits did not differ. There was no correlation with autism traits (no statistics were reported). | CARS total score |
| IL1-beta | Han et al. (2021)^51^ | plasma | There was no correlation with autism traits (Spearman’s rho=0.007, *p*=0.961). | CARS total score |
| IL1-beta | Napolioni et al. (2013)^67^ | plasma | The groups with ‘No correlation with autism traits (Spearman’s rho=0.236, *p*=0.362). | SRS total score |
| IL1-beta | Shmarina et al. (2020)^84^ | plasma | The groups with ‘mild/moderate’ (3.2 ± 1.6) and ‘severe’ traits (5.3 ± 1.6) did not differ (*p*=0.525). There were no correlations with the CARS total score (Spearman’s rho= 0.10, p>0.05) or the SCQ total score (rho=-0.04, *p*>0.05). | CARS total score, SCQ total score |
| IL1-beta | Suzuki et al. (2011)^91^ | plasma | There was no correlation with autism traits (No statistics were reported). | ADI-R total score |
| IL1-beta | Xie et al. (2017)^98^ | serum | There was no correlation with autism traits (Spearman’s rho=0.223, *p*=0.087). | ABC^(a)^ total score |
| IL1RA | Napolioni et al. (2013)^67^ | plasma | There was no correlation with autism traits (Spearman’s rho=-0.200, *p*=0.442). | SRS total score |
| IL1RA | Suzuki et al. (2011)^91^ | plasma | There was no correlation with autism traits (no statistics were reported). | ADI-R total score |
| IL2 | Cao et al. (2021) ^28^ | plasma | There was no correlation with autism traits (no statistics were reported). | CARS total score |
| IL2 | Napolioni et al. (2013)^67^ | plasma | There was no correlation with autism traits (Spearman’s rho=0.307, *p*=0.231). | SRS total score |
| IL4 | Ashwood et al. (2011)^21^ | plasma | There was a positive association with non-verbal communication difficulties indexed using the ADI-R (t = 2.61, *p* = 0.03), but there were no associations with other measures. | ABC^(b)^ domain score, ADI-R domain score, and ADOS module 1 domain score |
| IL4 | Cao et al. (2021) ^28^ | plasma | There was no negative correlation with autism traits (Spearman’s rho = -0.34, *p* < 0.05). | CARS total score |
| IL4 | Filippova et al. (2023)^43^ | plasma | The ‘mild/moderate’ group (median=2.72 IQR [2.40-3.07]) had higher cytokine concentration than the ‘severe’ (2.21 [2.00, 2.46]) trait group (*р* < 0.05). | CARS total score |
| IL4 | Guloksuz et al. (2017)^48^ | plasma | The groups with ‘mild/moderate’ and ‘severe’ traits did not differ. There was no correlation with autism traits (no statistics were reported). | CARS total score |
| IL4 | Napolioni et al. (2013)^67^ | plasma | There was no correlation with autism traits (Spearman’s rho=0.028, *p*=0.914). | SRS total score |
| IL5 | Cao et al. (2021) ^28^ | plasma | There was no correlation with autism traits (no statistics were reported). | CARS total score |
| IL5 | Napolioni et al. (2013)^67^ | plasma | There was no correlation with autism traits (Spearman’s rho=0.165, *p*=0.542). | SRS total score |
| IL5 | Suzuki et al. (2011)^91^ | plasma | There were no correlations with autism traits (no statistics were reported). | ADI-R total and domain score |
| IL6 | Alshammary et al. (2023)^13^ | serum | The groups with ‘mild’ (12.672 ± 0.598), ‘moderate’ (12.022 ± 0.544) and ‘severe’ traits (11.232 ± 0.733) did not differ (*p*s>0.27). | Clinical judgement based on several measures |
| IL6 | Anindya et al. (2025)^16^ | plasma | There was a positive correlation with autism traits (Spearman’s rho=0.20, p<0.05), indexed by the BAMBI, and a negative correlation with tactile sensitivity (rho=-0.22, p<0.01), indexed by the SSP | BAMBI, SSP |
| IL6 | Ashwood et al. (2011)^21^ | plasma | There was a positive association with stereotypy (t= 2.21, *p* = 0.05), indexed using the ABC domain score, but there were no associations with other measures. | ABC^(b)^ domain score, ADI-R domain score, and ADOS module 1 domain score |
| IL6 | Basheer et al. (2018)^24^ | serum | There was no correlation with autism traits (no statistics were reported). | CARS total score |
| IL6 | Cao et al. (2021) ^28^ | plasma | There was no correlation with autism traits (no statistics were reported). | CARS total score |
| IL6 | Carpita et al. (2023)^30^ | plasma | There was a positive correlation with mentalising difficulties (Spearman’s rho=0.346, p<0.05), indexed using the RAADS-14, although there were no correlations with other domain scores of social anxiety (rho=.124, p>0.05) and sensory reactivity (rho=0.179, p>0.05), and the RAADS-14 total score (rho=0.267, p>0.05). | RAADS-14 domain score |
| IL6 | Emanuele et al. (2010)^39^ | serum | There was no correlation with autism traits (no statistics were reported). All autistic adults are in the category of ‘severe’ (as defined by CARS>40) | ADI-R total score |
| IL6 | Filippova et al. (2023)^43^ | plasma | The ‘mild/moderate’ group (Median=1.89 IQR [0.67-3.06]) had lower cytokine concentration than the ‘severe’ (3.83 [2.31, 5.78]) trait group (*р* < 0.05). | CARS total score |
| IL6 | Gomez-Fernandez et al. (2018)^46^ | plasma | There were no correlations with autism traits (no statistics were reported). | ADOS raw score, PDDBI |
| IL6 | Guloksuz et al. (2017)^48^ | plasma | The groups with ‘mild/moderate’ and ‘severe’ traits did not differ. There was no correlation with autism traits (no statistics were reported). | CARS total score |
| IL6 | Han et al. (2021)^51^ | plasma | There was no correlation with autism traits (Spearman’s rho=0.275, *p*=0.058). | CARS total score |
| IL6 | Lochman et al. (2018)^62^ | serum | There were no correlations with autism traits (CARS: r=-0.13, ADOS: r=0.07; *p*s>0.05). | CARS total score, ADOS total raw score |
| IL6 | Napolioni et al. (2013)^67^ | plasma | There was no correlation with autism traits (Spearman’s rho=0.115, *p*=0.659). | SRS total score |
| IL6 | Ning et al. (2019)^69^ | serum | The ‘mild/moderate’ group (6.5±1.6) had a lower cytokine level than the ‘severe’ (7.5±1.5) autism trait group (*p=*0.002). | CARS total score |
| IL6 | Shaker et al. (2016)^81^ | serum | There were no correlations with stereotyped behaviours  (r=-0.1, *p*=0.6), communication (r=-0.19, *p*=0.3), social interaction (r=-0.07, *p*=0.71), and development (r=-0.15, *p*=0.43), indexed by GARS domain scores, and overall autism traits (r=-0.18, *p*=0.4), indexed by GARS total score. | GARS total and domain score |
| IL6 | Yan et al. (2015)^99^ | serum | There was a positive correlation with autism traits (Pearson’s *r=*0.274, *p* = 0.017). | CARS total score |
| IL6 | Yang et al. (2015) ^101^ | plasma | There was a positive correlation with autism traits (Spearman’s rho = 0.638, *p* < 0.001). | CARS total score |
| IL6 | Yang et al. (2015) ^100^ | plasma | There was a positive correlation with autism traits (Spearman’s rho = 0.63, *p* < 0.001). | CARS total score |
| IL7 | Basheer et al. (2018)^24^ | serum | There was no correlation with autism traits (no statistics were reported). | CARS total score |
| IL7 | Napolioni et al. (2013)^67^ | plasma | There was no correlation with autism traits (Spearman’s rho=0.109, *p*=0.676). | SRS total score |
| IL7 | Shen et al. (2021)^83^ | plasma | There were no correlations with autism traits (no statistics were reported). | SRS total or domain score |
| IL8 | Abd-Allah et al. (2020)^7^ | serum | The groups with ‘mild’ (62.7 ± 29.2), ‘moderate’ (59.9 ± 26.5) and ‘severe’ traits (54.8 ± 52.2) did not differ (p-value is not reported). | CARS total score |
| IL8 | Ashwood et al. (2011)^21^ | plasma | There were positive associations with lethargy (t = 2.58, p = 0.03), stereotypy (t = 2.56, p = 0.02), and hyperactivity (t = 2.89, *p* = 0.02), indexed using the ABC, but there were no associations with other measures. | ABC^(b)^ domain score, ADI-R domain score, and ADOS module 1 domain score |
| IL8 | Cao et al. (2021) ^28^ | plasma | There was no correlation with autism traits (no statistics were reported). | CARS total score |
| IL8 | Carissimi et al. (2019)^29^ | serum | There was no correlation with autism traits (no statistics were reported). | ADOS-2 raw score |
| IL8 | Gomez-Fernandez et al. (2018)^46^ | plasma | There were no correlations with autism traits (no statistics were reported). | ADOS raw score, PDDBI |
| IL8 | Han et al. (2021)^51^ | plasma | There was no correlation with autism traits (Spearman’s rho=0.039, p=0.793). | CARS total score |
| IL8 | Lv et al. (2025)^63^ | plasma | There were no correlations with autism traits (CARS: Spearman’s rho=0.049, p>0.05; ABC: rho=-0.140, p>0.05l SRS: rho=-0.144, p>0.05;) | CARS total score, ABC^(a)^ total score, SRS total score |
| IL8 | Napolioni et al. (2013)^67^ | plasma | There was no correlation with autism traits (Spearman’s rho=-0.338, *p*=0.185). | SRS total score |
| IL8 | Peng et al. (2021)^75^ | Serum | There was a negative correlation with autism traits (Pearson’s r=-0.842, *p*<0.001). | ADOS-2 module 1 raw scores |
| IL8 | Shen et al. (2021)^83^ | plasma | There were positive correlations with social awareness (β = 3.726, p = 0.021, R2 = 0.203), social cognition (β = 4.726, p = 0.022, R2 = 0.174), and social motivation (β = 5.104, p = 0.038, R2 = 0.143) | SRS subdomain scores |
| IL8 | Shmarina et al. (2020)^84^ | plasma | The ‘mild/moderate’ group (2401.6 ± 387.2) had lower cytokine concentration than the ‘severe’ (3966.0 ± 369.5) trait group (*р* =0.007) indexed by the CARS total score. There were no correlations with the CARS total score (Spearman’s rho= -0.21, p>0.05) or the SCQ total score (rho=-0.27, *p*>0.05). | CARS total score, SCQ total score |
| IL8 | Singh et al. (2017)^88^ | plasma | There were no associations with communication (z = 0.16, p = 0.871), social interactions (z = −0.15, p = 0.877), communication + social interactions (z = −0.06, p = 0.953), or stereotyped behaviour and restrictive interests (z= 0.75, p = 0.455). | ADOS raw score |
| IL8 | Zhang et al. (2023)^102^ | plasma | The groups with ‘mild/moderate’ (215.4 ± 563.3) and ‘severe’ traits (133.7 ± 456.2) and neurotypical development (1257.8 ± 3371.1) did not differ (*p*=0.171). | CARS total score |
| IL10 | Ashwood et al. (2011)^21^ | plasma | There was no correlation with autism severity. There was an association described by the author as a 'trend' with social interactions (t = 1.82, p = 0.07). | ABC^(b)^ domain score, ADI-R domain score, and ADOS module 1 domain score |
| IL10 | Cao et al. (2021) ^28^ | plasma | There was no correlation with autism traits (No statistics were reported). | CARS total score |
| IL10 | Carissimi et al. (2019)^29^ | serum | There was no correlation with autism traits (No statistics were reported). | ADOS-2 raw score |
| IL10 | El-Ansary et al. (2016)^38^ | plasma | There were no correlations with autism traits (no statistics were reported). | CARS total score, SRS total score |
| IL10 | Emanuele et al. (2010)^39^ | serum | There was no correlation with autism traits (no statistics were reported). All autistic adults are in the category of ‘severe’ (as defined by CARS>40) | ADI-R total score |
| IL10 | Filippova et al. (2023)^43^ | plasma | The ‘mild/moderate’ group (Median=7.75 IQR [6.38,10.42]) had higher cytokine concentration than the ‘severe’ (5.91 [3.20, 8.50]) trait group (*р* <0.05). | CARS total score |
| IL10 | Guloksuz et al. (2017)^48^ | plasma | The groups with ‘mild/moderate’ and ‘severe’ traits did not differ. There was no correlation with autism traits (no statistics were reported). | CARS total score |
| IL10 | Lochman et al. (2018)^62^ | serum | There was a negative correlation with autism traits indexed by the CARS total score (CARS: r=-0.39, p<0.05, ADOS: r=0.17, p>0.05) | CARS total score, ADOS total raw score |
| IL10 | Napolioni et al. (2013)^67^ | plasma | There was no correlation with autism traits (Spearman’s rho=0.191, *p*=0.494). | SRS total score |
| IL10 | Shmarina et al. (2020)^84^ | plasma | The ‘mild/moderate’ group (4.0 ± 0.6) had lower cytokine concentration than the ‘severe’ (9.8 ± 3.9) trait group (*р* =0.015) indexed by the CARS total score. There were no correlations with the CARS total score (Spearman’s rho= 0.13, p>0.05) or the SCQ total score (rho=-0.04, *p*>0.05). | CARS total score, SCQ total score |
| IL10 | Zhang et al. (2023)^102^ | plasma | The groups with ‘mild/moderate’ (20.62 ± 21.66) and ‘severe’ traits (23.42 ± 22.05) and neurotypical development (14.10 ± 13.78) did not differ (*p*=0.861). | CARS total score |
| IL11 | Napolioni et al. (2013)^67^ | plasma | There was no correlation with autism traits (Spearman’s rho=0.139, *p*=0.596). | SRS total score |
| IL12 | Ashaat et al. (2017)^18^ | plasma | The groups with ‘mild/moderate’ (27.3±8.11) and ‘severe’ traits (28±5.8) did not differ (*p*=0.954). | CARS total score |
| IL12 | El-Ansary et al. (2016)^38^ | plasma | There were no correlations with autism traits (no statistics were reported). | CARS total score, SRS total score |
| IL12 | Ibrahim et al. (2015)^55^ | serum | There was no correlation with autism traits (r = 0.032, p = 0.894). | CARS total score |
| IL12-beta | Lv et al. (2025)^63^ | plasma | There was a negative correlation with autism traits indexed by SRS total score (CARS: Spearman’s rho=-0.047, p>0.05; ABC: rho=-0.031, p>0.05; SRS: rho=-.332, p<0.05) | CARS total score  ABC^(a)^ total score  SRS total score |
| IL12p40 | Ashwood et al. (2011)^21^ | plasma | There were positive associations with lethargy (t = 2.41, *p* = 0.04) and stereotypy (t = 2.55, *p* = 0.05), indexed by the ABC domain scores. | ABC^(b)^ domain score, ADI-R domain score, and ADOS module 1 domain score |
| IL12p40 | Napolioni et al. (2013)^67^ | plasma | There was no correlation with autism traits (Spearman’s rho=-0.031, *p*=0.905). | SRS total score |
| IL12p70 | Cao et al. (2021) ^28^ | plasma | There was no correlation with autism traits (no statistics were reported). | CARS total score |
| IL12p70 | Napolioni et al. (2013)^67^ | plasma | There was no correlation with autism traits (Spearman’s rho=0.287, *p*=0.299). | SRS total score |
| IL12p70 | Suzuki et al. (2011)^91^ | plasma | There was no correlation with autism traits (no statistics were reported). | ADI-R |
| IL13 | Ashwood et al. (2011)^21^ | plasma | There was no association with autism traits (the author described an association with social interaction, indexed by the ADOS module 1 domain score, but this did not survive multiple comparison correction [t = 2.15, p = 0.06]) | ADOS module 1 raw score |
| IL13 | Napolioni et al. (2013)^67^ | plasma | There was no correlation with autism traits (Spearman’s rho=0.130, *p*=0.631). | SRS total score |
| IL13 | Suzuki et al. (2011)^91^ | plasma | There was no correlation with autism traits (no statistics were reported). | ADI-R |
| IL13 | Tobiasova et al. (2011)^93^ | serum | There were no significant differences in autism traits between groups stratified by cytokine level (above and below the median split; *p*s=0.17-0.91). | ADI-R total score, ABC^(b)^ domain score |
| IL15 | Napolioni et al. (2013)^67^ | plasma | There was no correlation with autism traits (Spearman’s rho=0.032, *p*=0.903). | SRS total score |
| IL16 | Napolioni et al. (2013)^67^ | plasma | There was no correlation with autism traits (Spearman’s rho=0.152, *p*=0.560). | SRS total score |
| IL17 | Ashaat et al. (2017)^18^ | plasma | The groups with ‘mild/moderate’ (1626.75±348.52) and ‘severe’ traits (1642.64±340.88) did not differ (*p*=0.969). | CARS total score |
| IL17 | Guloksuz et al. (2017)^48^ | plasma | The groups with ‘mild/moderate’ and ‘severe’ traits did not differ. There was no correlation with autism traits (no statistics were reported). | CARS total score |
| IL17 | Hashim et al. (2013)^53^ | plasma | There was a negative correlation with autism traits (Spearman’s rho=-0.39, p < 0.005) | CARS total score |
| IL17 | Napolioni et al. (2013)^67^ | plasma | There was no correlation with autism traits (Spearman’s rho=0.193, *p*=0.458). | SRS total score |
| IL17 | Suzuki et al. (2011)^91^ | plasma | There was no correlation with autism traits (no statistics were reported). | ADI-R total score |
| IL17A | Al-ayadhi & Mostafa (2012)^9^ | serum | The ‘mild/moderate’ group (1.5 [0.4]) had lower cytokine concentration than the and ‘severe’ (2.05 [2.3]) autism trait group (*p* < 0.001) | CARS total score |
| IL17A | Basheer et al. (2018)^24^ | serum | There was no correlation with autism traits (no statistics were reported). | CARS total score |
| IL17A | Cao et al. (2021) ^28^ | plasma | There was no correlation with autism traits (no statistics were reported). | CARS total score |
| IL17A | Li et al. (2024)^61^ | serum | The ‘mild/moderate’ group (1.32±0.57) had lower cytokine concentration than the ‘severe’ (1.68±0.59) autism trait group (*p* < 0.001). There was also a positive correlation with autism traits (Spearman’s *r* = 0.65, *p* < 0.001). | CARS total score |
| IL17A | Sallam et al. (2024)^79^ | serum | The groups with ‘mild/moderate’ (Median=  262.5 IQR [250, 320]) and ‘severe’ traits (260 [220, 300]) and neurotypical development (360 [320, 380]) did not differ (*p*=0.309). There was no correlation with autism traits  (r=0.097, *p*=0.654). | CARS total score |
| IL17A | Shmarina et al. (2020)^84^ | plasma | The ‘mild/moderate’ group (45.8 ± 20.1) had lower cytokine concentration than the ‘severe’ (69.9 ± 22.5) trait group (*р* =0.022) indexed by the CARS total score. There were no correlations with the CARS total score (Spearman’s rho= 0.13, p>0.05) or the SCQ total score (rho=-0.04, *p*>0.05). | CARS total score, SCQ total score |
| IL17A | Xie et al. (2017)^98^ | serum | There was no correlation with autism traits (r=0.184, p=0.158) | ABC^(a)^ total score |
| IL17A | Zhang et al. (2023)^102^ | plasma | The groups with ‘mild/moderate’ (Median=10.50 IQR [6.87, 19.23]) and ‘severe’ traits (6.07 [4.46, 9.62]) and neurotypical development (6.07 [4.46, 9.62]) did not differ (*p*=0.453). | CARS total score |
| IL17C | Lv et al. (2025)^63^ | plasma | There was a negative correlation with autism traits indexed by SRS total score (CARS: Spearman’s rho=-0.064, p>0.05; ABC: rho=-0.034, p>0.05; SRS: rho=-.280, p<0.05) | CARS total score, ABC^(a)^ total score, SRS total score |
| IL17F | Cao et al. (2021) ^28^ | plasma | There was no correlation with autism traits (no statistics were reported). | CARS total score |
| IL22 | Cao et al. (2021) ^28^ | plasma | There was no correlation with autism traits (no statistics were reported). | CARS total score |
| IL22 | Sallam et al. (2024)^79^ | serum | The groups with ‘mild/moderate’ (median=  750 IQR [660, 850]) and ‘severe’ traits (700 [600, 900]) and neurotypical development (1120 [920, 1150]) did not differ (*p*=0.309). However, there was a positive correlation with autism traits (r=0.432, *p*=0.035). | CARS total score |
| IL23 | Hashim et al. (2013)^53^ | plasma | There was a negative correlation with autism traits (Spearman’s rho=-0.56, p<0.0001) | CARS total score |
| IL2R | Denney et al. (1996)^32^ | plasma | There was a negative correlation with autism traits (r = -.50, *p* < 0.05). | ABC^(a)^ total score |
| IL2R | Han et al. (2021)^51^ | plasma | There was no correlation with autism traits (Spearman’s rho=0.238, p=0.104). | CARS total score |
| IL33 | Barbosa et al. (2015)^23^ | plasma | There was no correlation with autism traits (no statistics were reported). | SRS total score |
| IFN-alpha | Shen et al. (2021)^83^ | plasma | There were no correlations with autism traits (no statistics were reported). | SRS total or domain score |
| IFN-gamma | Basheer et al. (2018)^24^ | serum | There was no correlation with autism traits (no statistics were reported). | CARS total score |
| IFN-gamma | Cao et al. (2021) ^28^ | plasma | There was no correlation with autism traits (no statistics were reported). | CARS total score |
| IFN-gamma | Carissimi et al. (2019)^29^ | serum | There was no correlation with autism traits (no statistics were reported). | ADOS-2 raw score |
| IFN-gamma | Filippova et al. (2023)^43^ | plasma | The ‘mild/moderate’ group (Median=10.48 IQR [9.00, 11.48]) had lower cytokine concentration than the ‘severe’ (14.58 [11.99,15.93]) trait group (*р* <0.05). | CARS total score |
| IFN-gamma | Guloksuz et al. (2017)^48^ | plasma | The groups with ‘mild/moderate’ and ‘severe’ traits did not differ. There was no correlation with autism traits (no statistics were reported). | CARS total score |
| IFN-gamma | Lochman et al. (2018)^62^ | serum | There were no correlations with autism traits (CARS: r=-0.10, ADOS: r=0.01; *p*s>0.05) | CARS total score, ADOS total raw score |
| IFN-gamma | Napolioni et al. (2013)^67^ | plasma | There was no correlation with autism traits (Spearman’s rho=0.119, p=0.649). | SRS total score |
| IFN-gamma | Nour-Eldine et al. (2024)^71^ | plasma | The groups with ‘mild/moderate’ and ‘severe’ traits did not differ (no statistics were reported). | ADOS raw score |
| IFN-gamma | Shmarina et al. (2020)^84^ | plasma | The ‘mild/moderate’ group (51.9 ± 29.8) had lower cytokine concentration than the ‘severe’ (65.6±21.4) trait group (*р* =0.014) indexed by the CARS total score. There were no correlations with the CARS total score (Spearman’s rho= 0.15, p>0.05) or the SCQ total score (rho=-0.07, *p*>0.05). | CARS total score, SCQ total score |
| IFN-gamma | Zhang et al. (2023)^102^ | plasma | The groups with ‘mild/moderate’ (22.58 ± 35.41) and ‘severe’ traits (24.57 ± 42.07) and neurotypical development (10.24 ± 12.94) did not differ (*p*=0.873). | CARS total score |
| TNF-alpha | Abd-Allah et al. (2020)^7^ | serum | The groups with ‘mild’ (336.5 ± 56.5), ‘moderate’ (344.7 ± 51.7) and ‘severe’ traits (352.2 ± 50.8) did not differ (No p-value was reported). | CARS total score |
| TNF-alpha | Almashhady et al. (2025)^12^ | serum | The ‘mild’ (0.474±0.072), ‘moderate’ (0.584±0.105) and ‘severe’ (1.29 [1.15±0.49) trait groups significantly differed (*р*<0.0001). | CARS total score |
| TNF-alpha | Alshammary et al. (2023)^13^ | serum | The groups with ‘mild’ (8.354 ± 0.800), ‘moderate’ (8.270 ± 0.823) and ‘severe’ traits (7.959 ± 0.978) did not differ (*p*s>0.83). | Clinical judgement based on several measures |
| TNF-alpha | Anastasescu et al. (2024)^15^ | serum | There were no correlations with autism traits (no statistics were reported). | SCQ total score, ASRS |
| TNF-alpha | Cao et al. (2021) ^28^ | plasma | There were no correlations with autism traits (no statistics were reported). | CARS total score |
| TNF-alpha | Carissimi et al. (2019)^29^ | serum | There were no correlations with autism traits (no statistics were reported). | ADOS-2 raw score |
| TNF-alpha | Filippova et al. (2023)^43^ | plasma | The ‘mild/moderate’ group (Median=2.69 IQR [2.36, 3.60]) had higher cytokine concentration than the ‘severe’ (1.29 [1.17, 1.69]) trait group (*р* <0.05). | CARS total score |
| TNF-alpha | Ghaffari et al. (2016)^45^ | serum | There were no correlations with autism traits (no statistics were reported). | ADOS raw score, CARS |
| TNF-alpha | Gomez-Fernandez et al. (2018)^46^ | plasma | There were no correlations with autism traits (no statistics were reported). | ADOS raw score, PDDBI |
| TNF-alpha | Guloksuz et al. (2017)^48^ | plasma | The groups with ‘mild/moderate’ and ‘severe’ traits did not differ. There was no correlation with autism traits (no statistics were reported). | CARS total score |
| TNF-alpha | Hu et al. (2018)^54^ | plasma | There was a weak negative correlation with autism traits  (Pearson’s r=-0.220, p=.040, Pearson), although it became  between non-significant after outliers were removed (r=-0.102, *p*=0.350). | ADOS total calibrated severity score (CSS) |
| TNF-alpha | Lochman et al. (2018)^62^ | serum | There was a negative correlation with autism traits indexed by the CARS total score (CARS: r=-0.42, *p*<.05; ADOS: r=0.09, *p*>0.05) | CARS total score, ADOS total raw score |
| TNF-alpha | Lv et al. (2025)^63^ | plasma | There was a negative correlation with autism traits indexed by SRS total score (CARS: Spearman’s rho=0.013, p>0.05; ABC: rho=-0.161, p>0.05; SRS: rho=-.326, p<0.05) | CARS total score, ABC^(a)^ total score, SRS total score |
| TNF-alpha | Meguid et al. (2018)^65^ | serum | The group with atypical autism (6803.03±3434.1) had higher cytokine concentration compared to the ‘mild/moderate’ childhood autism group (3252.58±1396.1; *p*<.001), which in turn had higher cytokine than the ‘severe’ childhood autism trait group (1854.09±635.01; *p*<.005). Negative correlation with autism traits was observed in the childhood autism group (r=-0.490, *p*<.001). | CARS total score |
| TNF-alpha | Napolioni et al. (2013)^67^ | plasma | There was no correlation with autism traits (Spearman’s rho=0.110, p=0.673). | SRS total score |
| TNF-alpha | Xie et al. (2017)^98^ | serum | There were positive correlations with autism traits as indexed using total or domain score (Spearman’s rhos=0.290-0.400; *p*s=0.001-0.025). | ABC^(a)^ total or domain score |
| TNF-alpha | Yang et al. (2015) ^101^ | plasma | There was a positive correlation with autism traits (Spearman’s rho = 0.699, *p* < 0.001). | CARS total score |
| TNF-alpha | Zhang et al. (2023)^102^ | plasma | The groups with ‘mild/moderate’ (112.59 ± 99.79) and ‘severe’ traits (68.67 ± 33.52) and neurotypical development (112.59 ± 99.79) did not differ (*p*=0.873). | CARS total score |
| TNF-beta | Cao et al. (2021) ^28^ | plasma | There was no correlation with autism traits (no statistics were reported). | CARS total score |
| TNF-beta | Lv et al. (2025)^63^ | plasma | There were no correlations with autism traits (CARS: Spearman’s rho=0.005, p>0.05; ABC: rho=-0.172, p>0.05; SRS: rho=-0.199, p>0.05). | CARS total score, ABC^(a)^ total score, SRS total score |
| TNF-beta | Napolioni et al. (2013)^67^ | plasma | There was no correlation with autism traits (Spearman’s rho=0.257, p=0.319). | SRS total score |
| TWEAK | Artık et al. (2023)^17^ | plasma | There was no correlation with autism traits (Spearman’s rho=0.208; p=0.198) | CARS total score |
| TWEAK | Bilgiç et al. (2024)^26^ | serum | There was a negative correlation with social and self-help behaviour (Spearman’s rho=-0.302; p<.005) but not with other domains of the ABC (rhos=0.054 to 0.197; all *p*s>0.05). There was no correlation with CARS total score (rho=-0.014, p>0.05) | ABC^(a)^ domain and total score, and CARS total score |
| G-CSF | Napolioni et al. (2013)^67^ | plasma | There was no correlation with autism traits (Spearman’s rho=-0.361, p=0.155). | SRS total score |
| GM-CSF | Napolioni et al. (2013)^67^ | plasma | There was no correlation with autism traits (Spearman’s rho = 0.306, p=0.233). | SRS total score |
| M-CSF | Napolioni et al. (2013)^67^ | plasma | There was no correlation with autism traits (Spearman’s rho=-0.090, p=0.733). | SRS total score |
| CCL2/MCP1 | Gomez-Fernandez et al. (2018)^46^ | plasma | There were no correlations with autism traits (No statistics were reported). | ADOS raw score, PDDBI |
| CCL2/MCP1 | Han et al. (2022)^52^ | serum | There were no correlations with social interaction, communication, or stereotyped behaviour (Pearson’s *r*=0.144, *r*=0.20, or *r*=0.10, respectively; all *ps* > 0.05). | ADI-R domain score |
| CCL2/MCP1 | Han et al. (2017)^50^ | plasma | There were no correlations with social communication/ interaction (Spearman’s rho=0.13, *p*=0.58), and repetitive/ restricted behaviour (rho=0.17, *p*=0.46) | Composite scores of social interaction / communication and repetitive / restricted behaviour created from the SRS and the ADI-R scores |
| CCL2/MCP1 | Lochman et al. (2018)^62^ | serum | There was a negative correlation with autism traits indexed by the CARS total score (CARS: r=-0.42, p<0.05; ADOS: r=-0.19, p>0.05). | CARS total score, ADOS total raw score |
| CCL2/MCP1 | Napolioni et al. (2013)^67^ | plasma | There was no correlation with autism traits (Spearman’s rho=0.385, p=0.175). | SRS total score |
| CCL2/MCP1 | Peng et al. (2021)^75^ | serum | There was a positive correlation with autism traits (Pearson’s r=0.766, *p*<0.001). | ADOS-2 module 1 raw scores |
| CCL2/MCP1 | Shen et al. (2016)^82^ | plasma | There were no correlations with autism traits indexed by total or domain score (Pearson’s r=-0.037 to 0.239, *p*=.121 to .849). | SRS total or domain score |
| CCL3/MIP1-alpha | Ashwood et al. (2011)^22^ | plasma | There was a positive correlation with communication difficulties (Spearman’s rho = 0.292, *p* = 0.019), indexed by the ADOS module 1 raw score. There were no correlations with irritability, (Spearman’s rho= 0.028, *p*=0.733), lethargy (rho= 0.091, *p*=0.266), stereotypy (rho= 0.105, *p*=0.198), and hyperactivity (rho= 0.093, *p*=0.265), or inappropriate speech (rho= 0.023, *p*=0.783), indexed by the ABC domain scores. | ABC^(b)^ domain score, ADOS module 1 raw total score |
| CCL3/MIP1-alpha | Lv et al. (2025)^63^ | plasma | There were no correlations with autism traits (CARS: Spearman’s rho=-0.051, p>0.05; ABC: rho=-0.114, p>0.05; SRS: rho=-0.156, p>0.05) | CARS total score, ABC^(a)^ total score, SRS total score |
| CCL3/MIP1-alpha | Peng et al. (2021)^75^ | serum | There was no negative correlation with autism traits (Pearson’s r=-0.913, *p*<0.001). | ADOS-2 module 1 raw scores |
| CCL3/MIP1-alpha | Shen et al. (2016)^82^ | plasma | There were no correlations with autism traits indexed by total or domain score (Pearson’s r=-0.021 to 0.141, *p*=.466 to .940). | SRS total or domain score |
| CCL4/MIP1-beta | Napolioni et al. (2013)^67^ | plasma | There was a negative correlation with autism traits (Spearman’s rho=-0.524, *p*=0.031). | SRS total score |
| CCL4/MIP1-beta | Peng et al. (2021)^75^ | serum | There was a negative correlation with autism traits (Pearson’s r=-0.898, *p*<0.001). | ADOS-2 module 1 raw scores |
| CCL4/MIP1-beta | Shen et al. (2016)^82^ | plasma | There were no correlations with autism traits indexed by total or domain score (Pearson’s r=-0.193 to -0.061, *p*=.289 to .739). | SRS total or domain score |
| CCL4/MIP1-beta | Shen et al. (2021)^83^ | plasma | There were no correlations with autism traits (no statistics were reported). | SRS total or domain score |
| CCL5/RANTES | Ashwood et al. (2011)^22^ | plasma | There were positive correlations with lethargy (Spearman’s rho= 0.217, *p*=0.008), stereotypy (rho= 0.241, *p*=0.003), and hyperactivity (rho= 0.243, *p*=0.003), but not with irritability (rho= 0.137, *p*=0.101) or inappropriate speech (rho= 0.124, *p*=0.130), indexed by the ABC domain scores. There were also positive correlations with communication difficulties (rho = 0.256, *p*  = 0.041), indexed by the ADOS module 1 raw score, and autism traits indexed by the ADI-R (rho = 0.215, *p* = 0.05). | ABC^(b)^ domain score, ADI-R total score, ADOS module 1 raw total score |
| CCL5/RANTES | Gomez-Fernandez et al. (2018)^46^ | plasma | There were no correlations with autism traits (no statistics were reported). | ADOS raw score, PDDBI |
| CCL5/RANTES | Han et al. (2022)^52^ | serum | There were positive correlations with social interaction and communication (Pearson’s *r* = 0.35, *p* = 0.047 and *r* = 0.36; *p* = 0.041, respectively), but not with stereotyped behaviour (*r* = 0.28, *p*>0.05) | ADI-R domain score |
| CCL5/RANTES | Han et al. (2017)^50^ | plasma | There were no correlations with social communication/ interaction (Spearman’s rho= −0.13, *p*=0.58), and repetitive/ restricted behaviour (rho=−0.05, *p*=0.82) | Composite scores of social interaction / communication and repetitive / restricted behaviour created from the SRS and the ADI-R scores |
| CCL5/RANTES | Napolioni et al. (2013)^67^ | plasma | There was no correlation with autism traits (Spearman’s rho=-0.429, *p*=0.086). | SRS total score |
| CCL5/RANTES | Peng et al. (2021)^75^ | serum | There was no correlation with autism traits (Pearson's *r*=0.114, *p*= .377). | ADOS-2 Module 1 raw score |
| CCL5/RANTES | Shen et al. (2016)^82^ | plasma | There were no correlations with autism traits indexed by total or domain score (Pearson’s r=-0.202 to 0.058, *p*=.312 to .776). | SRS total or domain score |
| CCL8/MCP2 | Gomez-Fernandez et al. (2018)^46^ | plasma | There were no correlations with autism traits (No statistics were reported). | ADOS raw score, PDDBI |
| CCL11 | Lv et al. (2025)^63^ | plasma | There were no correlations with autism traits (CARS: Spearman’s rho=-0.099, p>0.05; ABC: rho=-0.185, p>0.05; SRS: rho=-0.1, p>0.05). | CARS total score, ABC^(a)^ total score, SRS total score |
| CCL17/TARC | Lochman et al. (2018)^62^ | serum | There was a negative correlation with autism traits indexed by the CARS total score (r=-0.44, *p* < 0.05), a positive correlation with the ADOS total score (r=0.76, *p* < 0.05) | CARS total score, ADOS total raw score |
| CCL17/TARC | Al-ayadhi & Mostafa (2013)^10^ | serum | The ‘mild/moderate’ group (1623 [651]) had higher cytokine concentration than the and ‘severe’ (1469 [1217]) autism trait group (*p* < 0.001). | CARS total score |
| CCL19 | Lv et al. (2025)^63^ | Plasma | There was a negative correlation with autism traits indexed by the SRS total score (CARS: Spearman’s rho=-0.086, p>0.05; ABC: rho=-0.1, p>0.05; SRS: rho=-.270, p<0.05) | CARS total score, ABC^(a)^ total score, SRS total score |
| CCL20 | Lv et al. (2025)^63^ | plasma | There were no correlations with autism traits (CARS: Spearman’s rho=-0.16, p>0.05; ABC: rho=-0.097, p>0.05; SRS: rho=-0.14, p>0.05) | CARS total score, ABC^(a)^ total score, SRS total score |
| CCL22/MDC | Al-ayadhi & Mostafa (2013)^10^ | serum | The ‘mild/moderate’ group (median 767.5 IQR [649]) had higher cytokine concentration than the and ‘severe’ (634 [451]) autism trait group (*p* < 0.001). | CARS total score |
| CCL25 | Lv et al. (2025)^63^ | plasma | There were no correlations with autism traits (CARS: Spearman’s rho=-0.084, p>0.05; ABC: rho=-0.13, p>0.05; SRS: rho=-0.07, p>0.05) | CARS total score, ABC^(a)^ total score, SRS total score |
| CXCL1/GRO-alpha | Suzuki et al. (2011)^91^ | plasma | There were no correlations with autism traits (No statistics were reported). | ADI-R |
| CXCL5/ENA-78 | Mostafa & Al-Ayadhi (2015)^66^ | serum | There was no correlation with autism traits (No statistics were reported). | CARS total score |
| CXCL6 | Lv et al. (2025)^63^ | plasma | There were no correlations with autism traits (CARS: Spearman’s rho=-0.079, p>0.05; ABC: rho=-0.027, p>0.05; SRS: rho=-0.131, p>0.05) | CARS, ABC^(a)^, SRS total scores |
| CXCL8 | Han et al. (2017)^50^ | plasma | There were no correlations with social communication/ interaction (Spearman’s rho= −0.02, *p*=0.92), and repetitive/ restricted behaviour (rho=0.16, *p*=0.47) | Composite scores of social interaction / communication and repetitive / restricted behaviour created from the SRS and the ADI-R scores |
| CXCL9/MIG | Han et al. (2017)^50^ | plasma | There were no correlations with social communication/ interaction (Spearman’s rho=−0.05, *p*=0.83), and repetitive/ restricted behaviour (rho=−0.22, *p*=0.34) | Composite scores of social interaction / communication and repetitive / restricted behaviour created from the SRS and the ADI-R scores |
| CXCL9/MIG | Lv et al. (2025)^63^ | plasma | There were no correlations with autism traits (CARS: Spearman’s rho=0.062, p>0.05; ABC: rho=-0.063, p>0.05; SRS: rho=-0.16, p>0.05) | CARS, ABC^(a)^, SRS total scores |
| CXCL9/MIG | Napolioni et al. (2013)^67^ | plasma | There was no correlation with autism traits (Spearman’s rho=-0.116, *p*=0.656). | SRS total score |
| CXCL9/MIG | Peng et al. (2021)^75^ | serum | There was a negative correlation with autism traits (Pearson’s r=-0.807, *p*<0.001). | ADOS-2 module 1 raw scores |
| CXCL9/MIG | Shen et al. (2016)^82^ | plasma | There were no correlations with autism traits indexed by the SRS total or domain score (Pearson’s r=-0.296 to 0.012, *p*=.126 to .950). | SRS total or domain score |
| CXCL10/IP10 | Peng et al. (2021)^75^ | serum | There was a negative correlation with autism traits (Pearson’s *r*=-0.851, *p*<0.001). | ADOS-2 module 1 raw scores |
| CXCL10/IP10 | Shen et al. (2016)^82^ | plasma | There were no correlations with autism traits indexed by the SRS total or domain score (Pearson’s r=-0.265 to 0.085, *p*=.142 to .920). | SRS total or domain score |
| CXCL10/IP10 | Shen et al. (2021)^83^ | plasma | There were no correlations with autism traits (no statistics were reported). | SRS total or domain score |
| CXCL10 | Han et al. (2017)^50^ | plasma | There were no correlations with social communication/ interaction (Spearman’s rho=0.28, *p*=0.20), and repetitive/ restricted behaviour (rho=0.03, *p*=0.89) | Composite scores of social interaction /communication and repetitive/ restricted behaviour created from the SRS and the ADI-R scores |
| CXCL12/SDF1 | Nour-Eldine et al. (2024)^71^ | plasma | The groups with ‘mild/moderate’ and ‘severe’ traits did not differ (no statistics were reported). | ADOS raw score |
| Eotaxin | Ashwood et al. (2011)^22^ | plasma | There were positive correlations with lethargy (Spearman’s rho= 0.203, *p*=0.013), stereotypy (rho= 0.159, *p*=0.050), and hyperactivity (rho= 0.222, *p*=0.007), but not with irritability (rho= 0.062, *p*=0.459) or inappropriate speech (rho= 0.111, *p*=0.175), indexed by the ABC domain scores. | ABC^(b)^ domain score |
| Eotaxin | Hu et al. (2018)^54^ | serum | There was a negative correlation with ADOS CSS social affect score (Spearman’s rho= -0.241, *p*=0.025). | ADOS total CSS |
| Eotaxin | Napolioni et al. (2013)^67^ | plasma | There was no correlation with autism traits (Spearman’s rho=-0.337, *p*=0.186). | SRS total score |
| Eotaxin | Nour-Eldine et al. (2024)^71^ | plasma | The groups with ‘mild/moderate’ and ‘severe’ traits did not differ (No statistics were reported). | ADOS raw score |
| Eotaxin | Shen et al. (2016)^82^ | plasma | There were no correlations with autism traits indexed by the SRS total or domain score (Pearson’s r=0.099 to 0.344, *p*=.058 to .596). | SRS total or domain score |
| Eotaxin-2 | Napolioni et al. (2013)^67^ | plasma | There was no correlation with autism traits (Spearman’s rho=0.233, *p*=0.368). | SRS total score |
| BDNF | Lochman et al. (2018)^62^ | serum | There was a positive correlation with autism traits indexed by the CARS total score (CARS: r=0.53, *p* < 0.05; ADOS: r=0.40, p>0.05) | CARS total score, ADOS total raw score |
| BNDF | Han et al. (2022)^52^ | serum | There were negative correlations with social interaction, communication and stereotyped behaviour (Pearson’s r=-0.49**, r=-0.47 **, and r=-0.39 *, respectively, *p*s from 0.004 to 0.027) | ADI-R domain score |
| EGF | Tobiasova et al. (2011)^93^ | serum | There were no significant differences in autism traits between groups stratified by cytokine level (above and below the median split; *p*s=0.08-0.59). | ADI-R total score, ABC^(b)^ domain score |
| FGF-2 | Nour-Eldine et al. (2024)^71^ | plasma | The groups with ‘mild/moderate’ and ‘severe’ traits did not differ (No statistics were reported). | ADOS raw score |
| HGF | Gomez-Fernandez et al. (2018)^46^ | plasma | There were no correlations with autism traits (no statistics were reported). | ADOS raw score, PDDBI |
| HGF | Nour-Eldine et al. (2024)^71^ | plasma | The groups with ‘mild/moderate’ and ‘severe’ traits did not differ (No statistics were reported). | ADOS raw score |
| HGF | Sugihara et al. (2007)^90^ | serum | There were no correlations with autism traits indexed by the ADI-R total and domain scores (no statistics were reported). | ADI-R total and domain score |
| PDGF-BB | Kajizuka et al. (2010)^56^ | serum | There was a positive correlation with repetitive/stereotyped behaviour (Pearson's *r*=0.532, *p*=0.001). | ADI-R total and domain score |
| PDGF-BB | Napolioni et al. (2013)^67^ | plasma | There was no correlation with autism traits (Spearman’s rho=-0.113, *p*=0.666). | SRS total score |
| TGF-beta | Ashwood et al. (2008)^20^ | plasma | There was a negative correlation with autism traits indexed by ABC total score (R=-0.393, p=0.038) but not with any measures  assessed by ADI-R or ADOS raw score | ABC^(b)^ total score, ADI-R, ADOS raw score |
| TGF-beta | Carissimi et al. (2019)^29^ | serum | There were no correlations with autism traits (no statistics were reported). | ADOS-2 raw score |
| TGF-beta | El Gohary et al. (2015)^33^ | plasma | There was a negative correlation with autism traits (r=-0.574, p=0.001). | CARS total score |
| TGF-beta | Han et al. (2017)^50^ | plasma | There were no correlations with social communication/ interaction (Spearman’s rho=0.32, *p*=0.15), and repetitive/ restricted behaviour (rho=0.23, *p*=0.31) | Composite scores of social interaction / communication and repetitive / restricted behaviour created from the SRS and the ADI-R scores |
| TGF-beta | Karadag et al. (2024)^57^ | serum | There was no correlation with autism traits (Spearman rho=0.042, p=0.793). | CARS total score |
| TGF-beta | Lochman et al. (2018)^62^ | serum | There were no correlations with autism traits (CARS: r=0.22, ADOS: r=-0.1; *p*s>0.05) | CARS total score, ADOS total raw score |
| TGF-beta | Okada et al. (2007)^72^ | serum | There were no correlations with autism total and domain scores (no statistics were reported). | ADI-R total and domain score |
| TGF-beta1 | Hashim et al. (2013)^53^ | plasma | There was a negative correlation with autism traits (Spearman’s rho=-0.55, *p*<0.0001) | CARS total score |
| VEGF | Simsek et al. (2021)^85^ | serum | There were no correlations with autism traits (Spearman’s rho=-0.229 to 0.011, *p*s=0.154 to 0.947). | CARS total score, ABC^(b)^ domain score |
| iGF | Simsek et al. (2021)^85^ | serum | There were no correlations with autism traits (Spearman’s rho=-0.220 to 0.026, *p*s=0.172 to 0.974). | CARS total score, ABC^(b)^ domain score |
| Adiponectin | Fujita-Shimizu et al. (2010)^44^ | serum | There was a negative correlation with social interaction score (r=−0.542, *p*=.014) | ADI-R total score |
| Resistin | Ghaffari et al. (2016)^45^ | serum | There were no correlations with autism traits (no statistics were reported). | ADOS raw score, CARS total score |
| MIF | Grigorenko et al. (2008)^47^ | plasma | There were positive correlations with social impairment, imaginative skills and total scores (r=0.41, 0.41, and 0.39, respectively; all *p*s<0.05). The was no correlation with stereotypical behaviour score (*r*=0.15, *p*>0.10) | ADOS raw score |
| MIF | Han et al. (2017)^50^ | plasma | There were no correlations with social communication/ interaction (Spearman’s rho=−0.10, *p*=0.65), and repetitive/ restricted behaviour (rho=0.12, *p*=0.60) | Composite scores of social interaction / communication and repetitive / restricted behaviour created from the SRS and the ADI-R scores |
| MIF | Ning et al. (2019)^69^ | serum | The ‘mild/moderate’ group (17.9±4.7) had a lower cytokine level than the ‘severe’ (28.1±8.5) autism trait group (*p*<0.001). There was also a positive correlation with autism traits (Pearson’s *r*=0.496; *p* < 0.001). | CARS total score |
| Abbreviations. Measures: ABC(a)=Autism Behavior Checklist, ABC(b)=Aberrant Behavior Checklist, ADI-R = Autism Diagnostic Interview--Revised, ADOS = Autism Diagnostic Observation Schedule, ASRS = Autism Spectrum Rating Scale, BAMBI = Brief Autism Mealtime Behavior Inventory, CARS = Childhood Autism Rating Scale, CSS=calibrated severity score, GARS = Gilliam Autism Rating Scale, PDDBI = Pervasive Developmental Disorder Behaviour Inventory, RAADS-14 = Ritvo Autism & Asperger Diagnostic Scale, SCQ = Social Communication Questionnaire, SRS = Social Responsiveness Scale, SSP = Short Sensory Profile, Cytokines: IL = interleukin, IFN = interferon, TNF = tumour necrosis factor, TWEAK = TNF-related weak inducer of apoptosis, G-/GM-/M-CSF = granulocyte-/granulocyte-macrophage/macrophage colony-stimulating factor, CCL = chemokine ligand, CXCL = C-X-C motif ligand, MCP = monocyte chemoattractant protein, MIP = macrophage inflammatory protein, RANTES = regulated upon activation, normal T cell expressed and presumably secreted, TARC = thymus- and activation-regulated chemokine, MDC = macrophage-derived chemokine, GRO = growth-regulated oncogene, ENA78 = Epithelial neutrophil-activating protein 78, MIG = monokine induced by gamma interferon, IP10 = interferon γ-induced protein 10, SDF = stromal cell-derived factor, BDNF = brain-derived neurotrophic factor E/F/H/PD/T/VE/iGF= epidermal/fibroglast/ hepatocyte/platelet-derived/transforming/vascular endothelial/insulin-like growth factor, RA = receptor agonist, MIF = macrophage migration inhibitory factor. Rows coloured blue present positive association(s), rows coloured light orange present negative association(s) between cytokine levels and autism traits. | | | | |

**References**

1. Masi A, Quintana DS, Glozier N, Lloyd AR, Hickie IB, Guastella AJ. Cytokine aberrations in autism spectrum disorder: a systematic review and meta-analysis. *Mol Psychiatry* 2015; **20**(4)**:** 440-446. doi: [10.1038/mp.2014.59](https://doi.org/10.1038/mp.2014.59).
2. Saghazadeh A, Ataeinia B, Keynejad K, Abdolalizadeh A, Hirbod-Mobarakeh A, Rezaei N. Anti-inflammatory cytokines in autism spectrum disorders: a systematic review and meta-analysis. *Cytokine* 2019; **123:** 154740. doi: [10.1016/j.cyto.2019.154740](https://doi.org/10.1016/j.cyto.2019.154740).
3. Saghazadeh A, Ataeinia B, Keynejad K, Abdolalizadeh A, Hirbod-Mobarakeh A, Rezaei N. A meta-analysis of pro-inflammatory cytokines in autism spectrum disorders: Effects of age, gender, and latitude. *J Psychiatr Res* 2019; **115:** 90-102. doi: [10.1016/j.jpsychires.2019.05.019](https://doi.org/10.1016/j.jpsychires.2019.05.019).

4.	Page MJ, McKenzie JE, Bossuyt PM, Boutron I, Hoffmann TC, Mulrow CD et al. The PRISMA 2020 statement: an updated guideline for reporting systematic reviews. *BMJ* 2021; **372:** n71. doi: [10.1136/bmj.n71](https://doi.org/10.1136/bmj.n71).

5.	Ribeiro CM, Beserra BTS, Silva NG, Lima CL, Rocha PRS, Coelho MS et al. Exposure to endocrine-disrupting chemicals and anthropometric measures of obesity: a systematic review and meta-analysis. *BMJ Open* 2020; **10**(6)**:** e033509. doi: [10.1136/bmjopen-2019-033509](https://doi.org/10.1136/bmjopen-2019-033509).

6.	Abdala RN, Al-Rekabi AAN, Al-Mosawi RAW. The relationship between pathological history and interleukin 17 in patients with autism. *Biochem Cell Arch* 2019; **19:** 49-52. Online publication accessed on 31 January 2025 from: <https://connectjournals.com/file_full_text/2944801H_49-52.pdf>.

7.	Abd-Allah NA, Ibrahim OM, Elmalt HA, Shehata MA, Hamed RA, Elsaadouni NM et al. Thioredoxin level and inflammatory markers in children with autism spectrum disorders. *Middle East Curr Psychiatr* 2020; **27**: 11. doi: [10.1186/s43045-020-00021-4](https://doi.org/10.1186/s43045-020-00021-4).

8.	Abdel-Salam OME, Youness ER, Abu-Elhamed WA. Changes in monocyte chemoattractive protein, nuclear respiratory factor 2, b-cell leukemia/lymphoma 2 and cholinesterase in serum of autistic children. *Biomed Pharmacol J* 2017; **10**(2)**:** 659-666. doi: [10.13005/bpj/1153](https://dx.doi.org/10.13005/bpj/1153).

9.	Al-Ayadhi LY, Mostafa GA. Elevated serum levels of interleukin-17A in children with autism. *J Neuroinflammation* 2012; **9:** 158. doi: [10.1186/1742-2094-9-158](https://doi.org/10.1186/1742-2094-9-158).

10.	Al-Ayadhi LY, Mostafa GA. Elevated serum levels of macrophage-derived chemokine and thymus and activation-regulated chemokine in autistic children. *J Neuroinflammation* 2013; **10:** 72. doi: [10.1186/1742-2094-10-72](https://doi.org/10.1186/1742-2094-10-72).

11.	Al-Bishri WM. Glucose transporter 1 deficiency, AMP-activated protein kinase activation and immune dysregulation in autism spectrum disorder: Novel biomarker sources for clinical diagnosis. *Saudi J Biol Sci* 2023; **30**(12): 103849. doi: [10.1016/j.sjbs.2023.103849](https://doi.org/10.1016/j.sjbs.2023.103849).

12.	Almashhady FR, Al-Hadrawi SW, Al-Dujaili AH. Diagnostic potential of serum TNFα and COX2 in ASD disorder. *Egypt J Med Microbiol* 2025; **34**(1): 213-217. doi**:** [10.21608/ejmm.2024.317029.1323](https://doi.org/10.21608/ejmm.2024.317029.1323).

13.	Alshammary RAA, Jouda J, Al-Salihy AAS. Some immunological and physiological parameters levels in Iraq children with autism spectrum disorders. *Int J Drug Deliv Technol* 2023; **13**(3): 955-960. Online publication accessed on 31 January 2025 from: [https://impactfactor.org/PDF/IJDDT/13/IJDDT,Vol13,Issue3, Article29.pdf](https://impactfactor.org/PDF/IJDDT/13/IJDDT,Vol13,Issue3,%20Article29.pdf).

14.	Alzghoul L, Aldahabi M, Albdour SA, Abdelhamid SS, Yanis AH, Qwaider YZ. The association between levels of inflammatory markers in autistic children compared to their unaffected siblings and unrelated healthy controls. *Turk J Med Sci* 2019; **49**(4)**:** 1047-1053. doi: [10.3906/sag-1812-167](https://doi.org/10.3906/sag-1812-167).

15.	Anastasescu CM, Gheorman V, Stoicanescu E-C, Popescu F, Gheorman V, Udristoiu I. Immunological biomarkers in autism spectrum disorder: the role of TNF-Alpha and dependent trends in serum IL-6 and CXCL8. *Life*; 2024; **14**(9). doi: [10.3390/life14091201](https://doi.org/10.3390/life14091201).

16.	Anindya I, Sekartini R, Ariyanto IA, Wiguna T, Sari NP, Rahayu YS et al. Cytomegalovirus-reactive IgG correlates with increased IL-6 and IL-1-beta levels, affecting eating behaviours and tactile sensitivity in children with autism. *Biomedicines;* 2025; **13**(2). doi: [10.3390/biomedicines13020338](https://doi.org/10.3390/biomedicines13020338).

17.	Artik A, Oztelcan Gunduz B, Mizrak S, Isik U. Increased serum levels of tumour necrosis factor-like weak inducer of apoptosis in children with autism spectrum disorder. *Int J Dev Disabil* 2023; **69**(4): 611-616. doi: [10.1080/20473869.2022.2143033](https://doi.org/10.1080/20473869.2022.2143033).

18.	Ashaat EA, El Ruby MO, Ibrahim SI, Taman KH, Kholoussi N, Zaki ME et al*.* Altered adaptive cellular immune function in a group of Egyptian children with autism. *J Clin Diagn Res* 2017; **11**(10)**:** SC14-SC17. doi: [10.7860/JCDR/2017/28124.10782](https://doi.org/10.7860/JCDR/2017/28124.10782).

19.	Ashwood P, Kwong C, Hansen R, Hertz-Picciotto I, Croen L, Krakowiak P et al. Brief report: plasma leptin levels are elevated in autism: association with early onset phenotype? *J Autism Dev Disord* 2008; **38**(1)**:** 169-175. doi: [10.1007/s10803-006-0353-1](https://doi.org/10.1007/s10803-006-0353-1).

20.	Ashwood P, Enstrom A, Krakowiak P, Hertz-Picciotto I, Hansen RL, Croen LA et al. Decreased transforming growth factor beta1 in autism: a potential link between immune dysregulation and impairment in clinical behavioral outcomes. *J Neuroimmunol* 2008; **204**(1)**:** 149-153. doi: [10.1016/j.jneuroim.2008.07.006](https://doi.org/10.1016/j.jneuroim.2008.07.006).

21.	Ashwood P, Krakowiak P, Hertz-Picciotto I, Hansen R, Pessah I, Van de Water J. Elevated plasma cytokines in autism spectrum disorders provide evidence of immune dysfunction and are associated with impaired behavioral outcome. *Brain Behav Immun* 2011; **25**(1)**:** 40-45.doi: [10.1016/j.bbi.2010.08.003](https://doi.org/10.1016/j.bbi.2010.08.003).

22.	Ashwood P, Krakowiak P, Hertz-Picciotto I, Hansen R, Pessah IN, Van de Water J. Associations of impaired behaviors with elevated plasma chemokines in autism spectrum disorders. *J Neuroimmunol* 2011; **232**(1-2)**:** 196-199. doi: [10.1016/j.jneuroim.2010.10.025](https://doi.org/10.1016/j.jneuroim.2010.10.025).

23.	Barbosa IG, Rodrigues DH, Rocha NP, Sousa LF, Vieira EL, Simoes ESAC et al. Plasma levels of alarmin IL-33 are unchanged in autism spectrum disorder: a preliminary study. *J Neuroimmunol* 2015; **278:** 69-72. doi: [10.1016/j.jneuroim.2014.11.021](https://doi.org/10.1016/j.jneuroim.2014.11.021).

24.	Basheer S, Venkataswamy MM, Christopher R, Van Amelsvoort T, Srinath S, Girimaji SC et al. Immune aberrations in children with autism spectrum disorder: a case-control study from a tertiary care neuropsychiatric hospital in India. *Psychoneuroendocrinology* 2018; **94:** 162-167. doi: [10.1016/j.psyneuen.2018.05.002](https://doi.org/10.1016/j.psyneuen.2018.05.002).

25.	Belica I, Jansakova K, Celusakova H, Kopcikova M, Polonyiova K, Raskova B et al. Plasma cytokine concentrations of children with autism spectrum disorder and neurotypical siblings. *Cytokine* 2023; **170:** 156333. doi: [10.1016/j.cyto.2023.156333](https://doi.org/10.1016/j.cyto.2023.156333).

26.	Bilgiç A, Katircioglu D, Tas MB, Kilinç I, Oflaz MB, Akça Ö. Serum BAFF, APRIL, TWEAK, TNFSF18, TNFR2, and TNFRS12A levels in preschool children with autism spectrum disorder. *Int J Dev Disabil* 2024; 1-9. doi: [10.1080/20473869.2024.2359143](https://doi.org/10.1080/20473869.2024.2359143).

27.	Blardi P, de Lalla A, Ceccatelli L, Vanessa G, Auteri A, Hayek J. Variations of plasma leptin and adiponectin levels in autistic patients. *Neurosci Lett* 2010; **479**(1)**:** 54-57. doi: [10.1016/j.neulet.2010.05.027](https://doi.org/10.1016/j.neulet.2010.05.027).

28.	Cao X, Liu K, Liu J, Liu YW, Xu L, Wang H et al*.* Dysbiotic gut microbiota and dysregulation of cytokine profile in children and teens with autism spectrum disorder. *Front Neurosci* 2021; **15:** 635925. doi: [10.3389/fnins.2021.635925](https://doi.org/10.3389/fnins.2021.635925).

29.	Carissimi C, Laudadio I, Palone F, Fulci V, Cesi V, Cardona F et al. Functional analysis of gut microbiota and immunoinflammation in children with autism spectrum disorders. *Dig Liver Dis* 2019; **51**(10)**:** 1366-1374. doi: [10.1016/j.dld.2019.06.006](https://doi.org/10.1016/j.dld.2019.06.006).

30.	Carpita B, Massoni L, Battaglini S, Palego L, Cremone IM, Massimetti G et al. IL-6, homocysteine, and autism spectrum phenotypes: An investigation among adults with autism spectrum disorder and their first-degree relatives. *CNS Spectrums* 2023; **28**(5): 620-628. doi: [10.1017/S1092852923000019](https://doi.org/10.1017/s1092852923000019).

31.	Croonenberghs J, Bosmans E, Deboutte D, Kenis G, Maes M. Activation of the inflammatory response system in autism. *Neuropsychobiology* 2002; **45**(1)**:** 1-6. doi: [10.1159/000048665](https://doi.org/10.1159/000048665).

32.	Denney DR, Frei BW, Gaffney GR. Lymphocyte subsets and interleukin-2 receptors in autistic children. *J Autism Dev Disord* 1996; **26**(1)**:** 87-97.

33.	El Gohary TM, El Aziz NA, Darweesh M, Sadaa ES. Plasma level of transforming growth factor β 1 in children with autism spectrum disorder. *Egypt J Ear Nose Throat Allied Sci* 2015; **16:** 69-73.

34.	El Wakkad ASED, Saleh MT. The proinflammatory cytokines in children with autism. *Pak J Bio Sci* 2006; **9:** 2593-2599.

35.	El-Ansary AK, Ben Bacha AG, Al-Ayadhi LY. Proinflammatory and proapoptotic markers in relation to mono and di-cations in plasma of autistic patients from Saudi Arabia. *J Neuroinflammation* 2011; **8:** 142. doi: [10.1186/1742-2094-8-142](https://doi.org/10.1186/1742-2094-8-142).

36.	El-Ansary A, Al-Ayadhi L. Neuroinflammation in autism spectrum disorders. *J Neuroinflammation* 2012; **9:** 265. doi: [10.1186/1742-2094-9-265](https://doi.org/10.1186/1742-2094-9-265).

37.	El-Ansary A, Al-Ayadhi L. GABAergic/glutamatergic imbalance relative to excessive neuroinflammation in autism spectrum disorders. *J Neuroinflammation* 2014; **11:** 189. doi: [10.1186/s12974-014-0189-0](https://doi.org/10.1186/s12974-014-0189-0).

38.	El-Ansary A, Hassan WM, Qasem H, Das UN. Identification of biomarkers of impaired sensory profiles among autistic patients. *PLoS One* 2016; **11**(11)**:** 19. doi: [10.1371/journal.pone.0164153](https://doi.org/10.1371/journal.pone.0164153).

39.	Emanuele E, Orsi P, Boso M, Broglia D, Brondino N, Barale F et al. Low-grade endotoxemia in patients with severe autism. *Neurosci Lett* 2010; **471:** 162-165.

40.	Enstrom A, Onore C, Hertz-Picciotto I, Hansen R, Croen L, Van de Water J et al. Detection of IL-17 and IL-23 in plasma samples of children with autism. *Am J Biochem Biotechnol* 2008; **4**(2)**:** 114-120.

41.	Enstrom A, Onore C, Tarver A, Hertz-Picciotto I, Hansen R, Croen L et al. Peripheral blood leukocyte production of bdnf following mitogen stimulation in early onset and regressive autism. *Am J Biochem Biotechnol* 2008; **4**(2)**:** 121-129.

42.	Ferencova N, Visnovcova Z, Ondrejka I, Hrtanek I, Bujnakova I, Kovacova V et al. Peripheral inflammatory markers in autism spectrum disorder and attention deficit/hyperactivity disorder at adolescent age. *Int J Mol Sci* 2023; **24**(14): 11710.

43.	Filippova YY, Alekseeva AS, Devyatova EV, Rusakova KA, Burmistrova AL. Saliva versus plasma cytokines as possible predictors of autism severity. *Med Immunol* 2023; **25**(5): 1213-1218. doi: [10.15789/1563-0625-SVP-2735](https://doi.org/10.15789/1563-0625-SVP-2735)

44.	Fujita-Shimizu A, Suzuki K, Nakamura K, Miyachi T, Matsuzaki H, Kajizuka M et al. Decreased serum levels of adiponectin in subjects with autism. *Prog Neuropsychopharmacol Biol Psychiatry* 2010; **34**(3)**:** 455-458.

45.	Ghaffari MA, Mousavinejad E, Riahi F, Mousavinejad M, Afsharmanesh MR. Increased serum levels of tumor necrosis factor-alpha, resistin, and visfatin in the children with autism spectrum disorders: a case-control study. *Neurol Res Int* 2016; **2016:** 9060751. doi: [10.1155/2016/9060751](https://doi.org/10.1155/2016/9060751).

46.	Gomez-Fernandez A, de la Torre-Aguilar MJ, Gil-Campos M, Flores-Rojas K, Cruz-Rico MD, Martin-Borreguero P et al. Children with autism spectrum disorder with regression exhibit a different profile in plasma cytokines and adhesion molecules compared to children without such regression. *Front Pediatr* 2018; **6:** 9. doi: [10.3389/fped.2018.00264](https://doi.org/10.3389/fped.2018.00264).

47.	Grigorenko EL, Han SS, Yrigollen CM, Leng L, Mizue Y, Anderson GM et al. Macrophage migration inhibitory factor and autism spectrum disorders. *Pediatrics* 2008; **122**(2)**:** e438-445. doi: [10.1542/peds.2007-3604](https://doi.org/10.1542/peds.2007-3604).

48.	Guloksuz SA, Abali O, Aktas Cetin E, Bilgic Gazioglu S, Deniz G, Yildirim A et al. Elevated plasma concentrations of S100 calcium-binding protein B and tumor necrosis factor alpha in children with autism spectrum disorders. *Braz J Psychiatry* 2017; **39**(3)**:** 195-200.

49.	Hamed NO, Laila Al A, Osman MA, Elkhawad AO, Bjorklund G, Qasem H et al. Determination of neuroinflammatory biomarkers in autistic and neurotypical Saudi children. *Metab Brain Dis* 2019; **34**(4)**:** 1049-1060.

50.	Han YM, Cheung WK, Wong CK, Sze SL, Cheng TW, Yeung MK et al. Distinct cytokine and chemokine profiles in autism spectrum disorders. *Front Immunol* 2017; **8:** 11. doi: [10.3389/fimmu.2017.00011](https://doi.org/10.3389/fimmu.2017.00011).

51.	Han Y, Xiong W, Liu J, Dai W, Su Y, Gao L et al. associations of serum cytokine levels and interleukin-6-572c/g polymorphism with myelin damage in Chinese children with autism spectrum disorder. *Neuroscience* 2021; **465:** 95-104.

52.	Han YMY, Yau S-Y, Chan MMY, Wong C-K, Chan AS. Altered cytokine and BDNF levels in individuals with autism spectrum disorders. *Brain Sci* 2022; **12**(4): 460. doi: [10.3390/brainsci12040460](https://doi.org/10.3390/brainsci12040460).

53.	Hashim H, Abdelrahman H, Mohammed D, Karam R. Association between plasma levels of transforming growth factor-beta 1, IL-23 and IL-17 and the severity of autism in Egyptian children. *Res Autism Spectr Disord* 2013; **7**(1)**:** 199-204.

54.	Hu CC, Xu X, Xiong GL, Xu Q, Zhou BR, Li CY et al. Alterations in plasma cytokine levels in chinese children with autism spectrum disorder. *Autism Res* 2018; **11**(7)**:** 989-999.

55.	Ibrahim S, El-Waleely T, Zakaria N, Ismail R. A study of serum interleukin-12 in a sample of autistic children in Egypt. *Egypt J Psychiatr* 2015; **36:** 81. doi: [10.4103/1110-1105.158115](http://dx.doi.org/10.4103/1110-1105.158115).

56.	Kajizuka M, Miyachi T, Matsuzaki H, Iwata K, Shinmura C, Suzuki K et al. Serum levels of platelet-derived growth factor BB homodimers are increased in male children with autism. *Prog Neuropsychopharmacol Biol Psychiatry* 2010; **34:** 154-158.

57.	Karadag M, Subasi Turgut F, Ciftci Z, Hangul Z, Taysi S. Comparison of Serum USP9x and TGF-beta Levels in Children with Autism Spectrum Disorders with Healthy Controls. *Noropsikiyatri Ars* 2024; 61(4): 306-312.

58.	Kareem JASA, Al-Thwani AN. Levels of serotonin and interleukin 6 Iraqi autistic children. *Pak J Biotechnol* 2018; **15**(2)**:** 531-534.

59.	Kartalcı G, Çalışkan Demir A, Kartalcı Ş, Üremiş N, Türköz Y. Evaluation of blood zonulin levels, inflammatory processes and neuronal changes in children with autism spectrum disorder. *Psychiatr Danub* 2022; **34**(2)**:** 279-287.

60.	Kordulewska NK, Kostyra E, Piskorz-Ogórek K, Moszynska M, Cieslinska A, Fiedorowicz E et al*.* Serum cytokine levels in children with spectrum autism disorder: Differences in pro- and anti-inflammatory balance. *J Neuroimmunol* 2019; **337:** 577066. doi: [10.1016/j.jneuroim.2019.577066](https://doi.org/10.1016/j.jneuroim.2019.577066)

61.	Li H, Dang Y, Yan Y. Serum interleukin-17 A and homocysteine levels in children with autism. *BMC Neurosci* 2024; **25**(1): 17.

62.	Lochman I, Svachova V, Milkova Pavlikova K, Medricka H, Novak V, Trilecova L et al*.* Serum cytokine and growth factor levels in children with autism spectrum disorder. *Med Sci Monitor* 2018; **24:** 2639-2646.

63.	Lv T, Wang M, Kui L, Wu J, Xiao Y. Novel inflammatory biomarkers for autism spectrum disorder detected by plasma olink proteomics. *Children* 2025; **12**(2): 210.

64.	Manzardo AM, Henkhaus R, Dhillon S, Butler MG. Plasma cytokine levels in children with autistic disorder and unrelated siblings. *Int J Dev Neurosci* 2012; **30**(2)**:** 121-127.

65.	Meguid NA, Ahmed HH, Gad MA, Baki OMA, Aziz SS, Eskander EF. Overview in the etiopathogenesis of different phenotypes of autism. *Biosci Res* 2018; **15**(4)**:** 2950-2970.

66.	Mostafa GA, Al-Ayadhi LY. The possible link between elevated serum levels of epithelial cell-derived neutrophil-activating peptide-78 (ENA-78/CXCL5) and autoimmunity in autistic children. *Behav Brain Funct* 2015; 11:11.

67.	Napolioni V, Ober-Reynolds B, Szelinger S, Corneveaux JJ, Pawlowski T, Ober-Reynolds S et al. Plasma cytokine profiling in sibling pairs discordant for autism spectrum disorder. *J Neuroinflammation* 2013; **10**: 38. doi: [10.1186/1742-2094-10-38](https://doi.org/10.1186/1742-2094-10-38).

68.	Nie ZQ, Han D, Zhang K, Li M, Kwon HK, Im SH et al. TH1/Treg ratio may be a marker of autism in children with immune dysfunction. *Res Autism Spectr Disord* 2023; 101: 102085. doi: [10.1016/j.rasd.2022.102085](https://doi.org/10.1016/j.rasd.2022.102085).

69.	Ning J, Xu L, Shen CQ, Zhang YY, Zhao Q. Increased serum levels of macrophage migration inhibitory factor in autism spectrum disorders. *Neurotoxicology* 2019; **71:** 1-5. doi: [10.1016/j.neuro.2018.11.015](https://doi.org/10.1016/j.neuro.2018.11.015).

70.	Noori AS, Rajabi P, Sargolzaei J, Alaghmand A. Correlation of biochemical markers and inflammatory cytokines in autism spectrum disorder (ASD). *BMC Pediatr* 2024; 24(1): 696. doi: [10.1186/s12887-024-05182-3](https://doi.org/10.1186/s12887-024-05182-3).

71.	Nour-Eldine W, Manaph NPA, Ltaief SM, Aati NA, Mansoori MH, Al Abdulla S et al. Discovery of a novel cytokine signature for the diagnosis of autism spectrum disorder in young Arab children in Qatar. *Front Psychiatry* 2024; 15:1333534. doi: [10.3389/fpsyt.2024.1333534](https://doi.org/10.3389/fpsyt.2024.1333534).

72.	Okada K, Hashimoto K, Iwata Y, Nakamura K, Tsujii M, Tsuchiya KJ et al. Decreased serum levels of transforming growth factor-beta1 in patients with autism. *Prog Neuropsychopharmacol Biol Psychiatry* 2007; **31**(1)**:** 187-190. doi: [10.1016/j.pnpbp.2006.08.020](https://doi.org/10.1016/j.pnpbp.2006.08.020).

73.	Pardo CA, Farmer CA, Thurm A, Shebl FM, Ilieva J, Kalra S et al. Serum and cerebrospinal fluid immune mediators in children with autistic disorder: A longitudinal study. *Mol Autism* 2017; **8**: 1. doi: [10.1186/s13229-016-0115-7](https://doi.org/10.1186/s13229-016-0115-7).

74.	Pecorelli A, Cervellati F, Belmonte G, Montagner G, Waldon P, Hayek J et al. Cytokines profile and peripheral blood mononuclear cells morphology in Rett and autistic patients. *Cytokine* 2016; **77:** 180-188. doi: [10.1016/j.cyto.2015.10.002](https://doi.org/10.1016/j.cyto.2015.10.002).

75.	Peng G, Peng X, Tong T, Zhang X, Xu M, Peng X. Correlation analysis of expression of CC and CXC chemokines in children with autism spectrum disorder. *Medicine* 2021; **100**(24)**:** e26391. doi: [10.1097/MD.0000000000026391](https://doi.org/10.1097/md.0000000000026391).

76.	Ramsey JM, Guest PC, Broek JA, Glennon JC, Rommelse N, Franke B et al. Identification of an age-dependent biomarker signature in children and adolescents with autism spectrum disorders. *Mol Autism* 2013; **4**(1)**:** 27. doi: [10.1186/2040-2392-4-27](https://doi.org/10.1186/2040-2392-4-27).

77.	Rodrigues DH, Rocha NP, Sousa LFD, Barbosa IG, Kummer A, Teixeira AL. Changes in adipokine levels in autism spectrum disorders. *Neuropsychobiology* 2014; **69**(1)**:** 6-10. doi: [10.1159/000356234](https://doi.org/10.1159/000356234).

78.	Russo AJ. Decreased epidermal growth factor (EGF) associated with HMGB1 and increased hyperactivity in children with autism. *Biomark Insights* 2013; **8:** 35-41. doi: [10.4137/BMI.S11270](https://doi.org/10.4137/BMI.S11270).

79.	Sallam DE, Shaker YS, Mostafa GA, El-Hossiny RM, Taha SI, Ahamed MAEH. Evaluation of serum interleukin-17 A and interleukin-22 levels in pediatric patients with autism spectrum disorder: a pilot study. *BMC Pediatr* 2024; **24**(1): 18. doi: [10.1186/s12887-023-04484-2](https://doi.org/10.1186/s12887-023-04484-2).

80.	Saresella M, Piancone F, Marventano I, Zoppis M, Hernis A, Zanette M et al. Multiple inflammasome complexes are activated in autistic spectrum disorders. *Brain Behav Immun* 2016; **57:** 125-133. doi: [10.1016/j.bbi.2016.03.009](https://doi.org/10.1016/j.bbi.2016.03.009).

81.	Shaker N, Taha G, Kholeif H, Sayed NM , Sheikh MM, Abulmagd ML. Serum levels of s100b, interleukin-6 and anti-transglutaminase Ii Iga as immune markers in a sample of Egyptian children with autistic spectrum disorders. *Autism* 2016; **6**(5): 1000191. doi: [10.4172 /2165-7890.1000191](http://dx.doi.org/10.4172/2165-7890.1000191).

82.	Shen Y, Oua J, Liu M, Shi L, Li Y, Xiao L et al. Altered plasma levels of chemokines in autism and their association with social behaviors. *Psychiatry Res* 2016; **244:** 300-305. doi: [10.1016/j.psychres.2016.07.057](https://doi.org/10.1016/j.psychres.2016.07.057).

83.	Shen Y, Li Y, Shi L, Liu M, Wu R, Xia K et al. Autism spectrum disorder and severe social impairment associated with elevated plasma interleukin-8. *Pediatr Res* 2021; **89**(3)**:** 591-597. doi: [10.1038/s41390-020-0910-x](https://doi.org/10.1038/s41390-020-0910-x).

84.	Shmarina GV, Ershova ES, Simashkova NV, Nikitina SG, Chudakova JM, Veiko NN et al. Oxidized cell-free DNA as a stress-signaling factor activating the chronic inflammatory process in patients with autism spectrum disorders. *J Neuroinflammation* 2020; **17**(1)**:** 212. doi: [10.1186/s12974-020-01881-7](https://doi.org/10.1186/s12974-020-01881-7)

85.	Simsek F, Isik U, Aktepe E, Kilic F, Sirin FB, Bozkurt M. Comparison of serum VEGF, IGF-1, and HIF-1 alpha levels in children with autism spectrum disorder and healthy controls. *J Autism Dev Disord* 2021; **51**(10): 3564-3574. doi: [10.1007/s10803-020-04820-w](https://doi.org/10.1007/s10803-020-04820-w).

86.	Singh VK, Warren RP, Odell JD, Cole P. Changes of soluble interleukin-2, interleukin-2 receptor, T8 antigen, and interleukin-1 in the serum of autistic children. *Clin Immunol Immunopathol* 1991; **61**(3)**:** 448-455. doi: [10.1016/s0090-1229(05)80015-7](https://doi.org/10.1016/s0090-1229(05)80015-7).

87.	Singh VK. Plasma increase of interleukin-12 and interferon-gamma pathological significance in autism. *J Neuroimmunol* 1996; **66:** 143-145. doi: [10.1016/0165-5728(96)00014-8](https://doi.org/10.1016/0165-5728(96)00014-8).

88.	Singh S, Yazdani U, Gadad B, Zaman S, Hynan LS, Roatch N et al. Serum thyroid-stimulating hormone and interleukin-8 levels in boys with autism spectrum disorder. *J Neuroinflammation* 2017; **14**(1)**:** 113. doi: [10.1186/s12974-017-0888-4](https://doi.org/10.1186/s12974-017-0888-4).

89.	Steeb H, Ramsey JM, Guest PC, Stocki P, Cooper JD, Rahmoune H et al. Serum proteomic analysis identifies sex-specific differences in lipid metabolism and inflammation profiles in adults diagnosed with Asperger syndrome. *Mol Autism* 2014; **5**(1):4. doi: [10.1186/2040-2392-5-4](https://doi.org/10.1186/2040-2392-5-4).

90.	Sugihara G, Hashimoto K, Iwata Y, Nakamura K, Tsujii M, Tsuchiya KJ et al. Decreased serum levels of hepatocyte growth factor in male adults with high-functioning autism. *Prog Neuropsychopharmacol Biol Psychiatry* 2007; **31**(2)**:** 412-415. doi: [10.1016/j.pnpbp.2006.10.010](https://doi.org/10.1016/j.pnpbp.2006.10.010).

91.	Suzuki K, Matsuzaki H, Iwata K, Kameno Y, Shimmura C, Kawai S et al. Plasma cytokine profiles in subjects with high-functioning autism spectrum disorders. *PLoS One* 2011; **6**(5)**:** e20470. doi: [10.1371/journal.pone.0020470](https://doi.org/10.1371/journal.pone.0020470).

92.	Sweeten TL, Posey DJ, Shankar S, McDougle CJ. High nitric oxide production in autistic disorder: a possible role for interferon-gamma. *Biol Psychiatry* 2004; **55**(4)**:** 434-437. doi: [10.1016/j.biopsych.2003.09.001](https://doi.org/10.1016/j.biopsych.2003.09.001)

93.	Tobiasova Z, Zhang Y, Chae W, Bothwell ALM, Kawikova I, Leckman JF et al*.* Risperidone-related improvement of irritability in children with autism is not associated with changes in serum of epidermal growth factor and interleukin-13. *J Child Adolesc Psychopharmacol* 2011; **21**(6)**:** 555-564. doi: [10.1089/cap.2010.0134](https://doi.org/10.1089/cap.2010.0134).

94.	Tonhajzerova I, Ondrejka I, Mestanik M, Mikolka P, Hrtanek I, Mestanikova A et al. Inflammatory activity in autism spectrum disorder. *Adv Exp Med Biol* 2015; **861:** 93-98. doi: [10.1007/5584_2015_145](https://doi.org/10.1007/5584_2015_145).

95.	Tostes MH, Teixeira HC, Gattaz WF, Brandao MA, Raposo NR. Altered neurotrophin, neuropeptide, cytokines and nitric oxide levels in autism. *Pharmacopsychiatry* 2012; **45**(6)**:** 241-243. doi: [10.1055/s-0032-1301914](https://doi.org/10.1055/s-0032-1301914).

96.	Tsilioni I, Taliou A, Francis K, Theoharides TC. Children with autism spectrum disorders, who improved with a luteolin-containing dietary formulation, show reduced serum levels of TNF and IL-6. *Transl Psychiatry* 2015; **5**(9)**:** e647. doi: [10.1038/tp.2015.142](https://doi.org/10.1038/tp.2015.142).

97	Wang B, Qin Y, Chen Y, Zheng X, Chen Y, Zhao J et al. Adipose tissue may not be a major player in the inflammatory pathogenesis of autism spectrum disorder. *Brain Behav Immun Health* 2025; **43**: 100929. doi: [10.1016/j.bbih.2024.100929](https://emckclac-my.sharepoint.com/personal/k1197794_kcl_ac_uk/Documents/Collaborations/Theresia/8_Writing%20up/Resubmission/10.1016/j.bbih.2024.100929).

98.	Xie J, Huang L, Li XH, Li H, Zhou YM, Zhu H et al. Immunological cytokine profiling identifies TNF-alpha as a key molecule dysregulated in autistic children. *Oncotarget* 2017; **8**(47)**:** 82390-82398. doi: [10.18632/oncotarget.19326](https://doi.org/10.18632/oncotarget.19326)

99.	Yan C-L, Zhang J, Hou Y. Decreased plasma levels of lipoxin A4 in children with autism spectrum disorders. *NeuroReport* 2015; **26**(6): 341-345. doi: [10.1097/WNR.0000000000000350](https://doi.org/10.1097/wnr.0000000000000350)

100.	Yang CJ, Liu CL, Sang B, Du YJ, Zhu XM. The combined role of serotonin and interleukin-6 as biomarker for autism. *Neuroscience* 2015; **284:** 290-296. doi: [10.1016/j.neuroscience.2014.10.011](https://doi.org/10.1016/j.neuroscience.2014.10.011).

101.	Yang CJ, Tan HP, Yang FY, Liu CL, Sang B, Zhu XM et al. The roles of cortisol and pro-inflammatory cytokines in assisting the diagnosis of autism spectrum disorder. *Res Autism Spectr Disord* 2015; **9:** 174-181. doi: [10.1016/j.rasd.2014.10.012](https://doi.org/10.1016/j.rasd.2014.10.012).

102.	Zhang L, Xu Y, Sun S, Liang C, Li W, Li H et al. Integrative analysis of γδT cells and dietary factors reveals predictive values for autism spectrum disorder in children. *Brain Behav Immun* 2023; 111: 76-89. doi: [10.1016/j.bbi.2023.03.029](https://doi.org/10.1016/j.bbi.2023.03.029).

103.	Zurita MF, Cárdenas PA, Sandoval ME, Peña MC, Fornasini M, Flores N et al. Analysis of gut microbiome, nutrition and immune status in autism spectrum disorder: a case-control study in Ecuador. *Gut Microbes* 2020; **11:** 453-464. doi: [10.1080/19490976.2019.1662260](https://doi.org/10.1080/19490976.2019.1662260).

104.	Al Olaby RR, Hagerman R, Abbeduto L, Tassone F,. Identification of molecular biomarkers predictive of response to targeted treatment in Fragile X syndrome and autism spectrum disorder. *J Intellect Disabil Res* 2017; **61**(9)**:** 828. doi: <https://doi.org/10.1111/jir.12400>

105.	Al Olaby RR, Jiraanont P, Durbin-Johnson B, Jasoliya M, Tang HT, Hagerman R et al. Molecular biomarkers predictive of sertraline treatment response in young children with autism spectrum disorder. *Front Genet* 2020; **11:** 308. doi: [10.3389/fgene.2020.00308](https://doi.org/10.3389/fgene.2020.00308).

106.	Al-Ayadhi L, Alhowikan AM, Halepoto DM. Impact of auditory integrative training on transforming growth factor-beta1 and its effect on behavioral and social emotions in children with autism spectrum disorder. *Med Princ Pract* 2018; **27**(1)**:** 23-29. doi: [10.1159/000486572](https://doi.org/10.1159/000486572).

107.	Al-Ayadhi LY. Pro-inflammatory cytokines in autistic children in central Saudi Arabia. *Neurosciences* 2005; **10:** 155-158. Online publication accessed 31 January 2025 from <https://nsj.org.sa/content/nsj/10/2/155.full.pdf>

108.	Aldossari AA, Ansari MA, Nadeem A, Attia SM, Bakheet SA, Al-Ayadhi LY et al. Upregulation of inflammatory mediators in peripheral blood cd40+ cells in children with autism spectrum disorder. *Int J Mol Sci* 2023; **24**(8)**:** 7475. doi: [10.3390/ijms24087475](https://doi.org/10.3390/ijms24087475).

109.	Alekseeva AS, Filippova YY, Burmistrova AL. Expression of key molecules of the classical inflammation activation pathway in blood leukocytes of autistic children. *Russ J Immunol* 2024; **27**(4)**:** 825-830. doi: [10.46235/1028-7221-16783-EOK](https://doi.org/10.46235/1028-7221-16783-EOK).

110.	Ansari S, AdibSaber F, Elmieh A, Gholamrezaei S. The effect of water-based intervention on sleep habits and two sleep-related cytokines in children with autism. *Sleep Med* 2021; **82:** 78-83. doi: [10.1016/j.sleep.2021.03.045](https://doi.org/10.1016/j.sleep.2021.03.045).

111.	Ashwood P. Differential T cell levels of tumor necrosis factor receptor-II in children with autism. *Front Psychiatry* 2018; **9**: 543. doi: [10.3389/fpsyt.2018.00543](https://doi.org/10.3389/fpsyt.2018.00543).

112.	Balestrieri E, Cipriani C, Matteucci C, Bucci I, Miele MT, Grelli S et al. Children with autism spectrum disorder and their mothers share abnormal expression of selected endogenous retroviruses families and cytokines. *Front Immunol* 2019; **10:** 2244. doi: [10.3389/fimmu.2019.02244](https://doi.org/10.3389/fimmu.2019.02244).

113.	Bashir S, Al-Ayadhi LY. Effect of camel milk on thymus and activation-regulated chemokine in autistic children: double-blind study. *Pediatr Res* 2014; **75**(4)**:** 559-563. doi: [10.1038/pr.2013.248](https://doi.org/10.1038/pr.2013.248).

114.	Bryn V, Halvorsen B, Ueland T, Isaksen J, Kolkova K, Ravn K et al. Brain derived neurotrophic factor (BDNF) and autism spectrum disorders (ASD) in childhood. *Eur J Paediatr Neurol* 2015; **19**(4)**:** 411-414. DOI: [10.1016/j.ejpn.2015.03.005](https://doi.org/10.1016/j.ejpn.2015.03.005).

115.	Bryn V, Aass HC, Skjeldal OH, Isaksen J, Saugstad OD, Ormstad H. Cytokine profile in autism spectrum disorders in children. *J Mol Neurosci* 2017; **61**(1)**:** 1-7. doi: [10.1007/s12031-016-0847-z](https://doi.org/10.1007/s12031-016-0847-z).

116.	Businaro R, Corsi M, Azzara G, Di Raimo T, Laviola G, Romano E et al. Interleukin-18 modulation in autism spectrum disorders. *J Neuroinflammation* 2016; **13:** 2. doi: [10.1186/s12974-015-0466-6](https://doi.org/10.1186/s12974-015-0466-6).

117.	Cherevko NA, Novikov PS, Khudyakova MI, Arkhipov AM, Loginova EA, Vekovtsev AA et al. The influence of modulation of intestinal microbiota on clinical and immunological parameters and oxytocin levels in children with autism spectrum disorders. *Russ J Immunol* 2024; **27**(4)**:** 831-838. doi: [10.46235/1028-7221-16873-TIO](https://doi.org/10.46235/1028-7221-16873-TIO).

118.	Chez MG, Dowling T, Patel PB, Khanna P, Kominsky M. Elevation of tumor necrosis factor-alpha in cerebrospinal fluid of autistic children. *Pediatr Neurol* 2007; **36**(6)**:** 361-365. doi: [10.1016/j.pediatrneurol.2007.01.012](https://doi.org/10.1016/j.pediatrneurol.2007.01.012).

119.	Choi JE, Widjaja F, Careaga M, Bent S, Ashwood P, Hendren RL. Change in plasma cytokine levels during risperidone treatment in children with autism. *J Child Adolesc Psychopharmacol* 2014; **24**(10)**:** 586-589. doi: [10.1089/cap.2013.0108](https://doi.org/10.1089/cap.2013.0108).

120.	Chudakova YM, Nikitina SG, Balakireva EE, Shmarin VV, Salimova TA, Martynov AV et al. Immune alterations in children with autism spectrum disorders. *Russ J Immunol* 2024; **27**(4)**:** 819-824. doi: [10.46235/1028-7221-16837-IAI](https://doi.org/10.46235/1028-7221-16837-IAI).

121.	Dardani C, Robinson J, Zheng J, Sadik A, Pagoni P, Stergiakouli E et al. Immunological pathways underlying autism: findings from Mendelian randomization and genetic colocalisation analyses. *MedRxiv* 2022. doi: [10.1101/2022.02.16. 22271031](https://doi.org/10.1101/2022.02.16.%2022271031).

122.	Eftekharian MM, Komaki A, Ghafouri-Fard S, Omrani MD, Arsang-Jang S, Taheri M. Expression analysis of suppressor of cytokine signaling (socs) genes in blood of autistic patients. *Adv Neuroimmune Biol* 2020; **7**(3)**:** 149-154. doi: [10.3233/NIB-180144](https://doi.org/10.3233/NIB-180144).

123.	Ferguson BJ, Marler S, Altstein LL, Lee EB, Mazurek MO, McLaughlin A et al. Associations between cytokines, endocrine stress response, and gastrointestinal symptoms in autism spectrum disorder. *Brain Behav Immun* 2016; **58:** 57-62. doi: [10.1016/j.bbi.2016.05.009](https://doi.org/10.1016/j.bbi.2016.05.009).

124.	Filippova YY, Alekseeva AS, Burmistrova AL. Leukocyte cytokine expression is associated with severity of autism in children. *Russ J Immunol* 2023; **26**(4)**:** 593-598. doi: [10.46235/1028-7221-13911-LCE](https://doi.org/10.46235/1028-7221-13911-LCE).

125.	Hassan WM, Al-Ayadhi L, Bjørklund G, Alabdali A, Chirumbolo S, El-Ansary A. The use of multi-parametric biomarker profiles may increase the accuracy of ASD prediction. *J Mol Neurosci* 2018, **66**(1):85-101. doi: [10.1007/s12031-018-1136-9](https://doi.org/10.1007/s12031-018-1136-9).

126.	Inoue R, Sakaue Y, Kawada Y, Tamaki R, Yasukawa Z, Ozeki M et al. Dietary supplementation with partially hydrolyzed guar gum helps improve constipation and gut dysbiosis symptoms and behavioral irritability in children with autism spectrum disorder. *J Clin Biochem Nutr* 2019; **64**(3)**:** 217-223. doi: [10.3164/jcbn.18-105](https://doi.org/10.3164/jcbn.18-105).

127.	Jácome MCI, Chacòn LMM, Cuesta HV, Rizo CM, Santiesteban MW, Hernandez LR et al. Peripheral inflammatory markers contributing to comorbidities in autism. *Behav Sci* 2016; **6**(4)**:** 29. doi: [10.3390/bs6040029](https://doi.org/10.3390/bs6040029).

128.	Javadfar Z, Abdollahzad H, Moludi J, Rezaeian S, Amirian H, Foroughi AA et al. Effects of vitamin D supplementation on core symptoms, serum serotonin, and interleukin-6 in children with autism spectrum disorders: a randomized clinical trial. *Nutrition* 2020; **79-80:** 110986. doi: [10.1016/j.nut.2020.110986](https://doi.org/10.1016/j.nut.2020.110986).

129.	Kerley CP, Power C, Gallagher L, Coghlan D. Lack of effect of vitamin D3 supplementation in autism: a 20-week, placebo-controlled RCT. *Arch Dis Child* 2017; **102**(11)**:** 1030-1036. doi: [10.1136/archdischild-2017-312783](https://doi.org/10.1136/archdischild-2017-312783).

130.	Kılınç K, Türkoğlu S, Kocabaş R, Güler HA, Yılmaz Ç, Büyükateş A. What are the levels and interactions of neuroligin-1, neuroligin-3, and inflammatory cytokines (IL-6, IL-8) in children diagnosed with autism spectrum disorder? *Prog Neuropsychopharmacol Biol Psychiatry* 2025; **137**:111275. doi: [10.1016/j.pnpbp.2025.111275](https://doi.org/10.1016/j.pnpbp.2025.111275)

131.	Kim DHJ, Iosif AM, Ramirez-Celis A, Ashwood P, Ames JL, Lyall K et al. Neonatal immune signatures differ by sex regardless of neurodevelopmental disorder status: Macrophage migration inhibitory factor (MIF) alone reveals a sex by diagnosis interaction effect. *Brain Behav Immun* 2023; **111**: 328-333. doi: [10.1016/j.bbi.2023.05.002](https://doi.org/10.1016/j.bbi.2023.05.002)

132.	Kong XJ, Liu J, Liu K, Koh M, Sherman H, Liu S et al*.* Probiotic and oxytocin combination therapy in patients with autism spectrum disorder: a randomized, double-blinded, placebo-controlled pilot trial. *Nutrients* 2021; **13**(5):1552. doi: [10.3390/nu13051552](https://doi.org/10.3390/nu13051552).

133.	Long J, Dang H, Su W, Moneruzzaman M, Zhang H. Interactions between circulating inflammatory factors and autism spectrum disorder: a bidirectional Mendelian randomization study in European population. *Front Immunol* 2024; **15**: 1370276. doi: [10.3389/fimmu.2024.1370276](https://doi.org/10.3389/fimmu.2024.1370276).

134.	Malek M, Ashraf-Ganjouei A, Moradi K, Bagheri S, Mohammadi M-R, Akhondzadeh S. Prednisolone as adjunctive treatment to risperidone in children with regressive type of autism spectrum disorder: a randomized, placebo-controlled trial. *Clin Neuropharmacol* 2020; **43**(2)**:** 39-45. doi: [10.1097/WNF.0000000000000382](https://doi.org/10.1097/wnf.0000000000000382)

135.	Mankad D, Dupuis A, Smile S, Roberts W, Brian J, Lui T et al. A randomized, placebo controlled trial of omega-3 fatty acids in the treatment of young children with autism. *Mol Autism* 2015; **6**(1)**:** 18. doi: [10.1186/s13229-015-0010-7](https://doi.org/10.1186/s13229-015-0010-7).

136.	Masi A, Breen EJ, Alvares GA, Glozier N, Hickie IB, Hunt A et al. Cytokine levels and associations with symptom severity in male and female children with autism spectrum disorder. *Mol Autism* 2017; **8**(1)**:** 63. doi: [10.1186/s13229-017-0176-2](https://doi.org/10.1186/s13229-017-0176-2).

137.	Mazahery H, Conlon CA, Beck KL, Mugridge O, Kruger MC, Stonehouse W et al. Inflammation (IL-1 beta) modifies the effect of vitamin D and omega-3 long chain polyunsaturated fatty acids on core symptoms of autism spectrum disorder-an exploratory pilot study. *Nutrients* 2020; **12**(3)**:** 17. doi: [10.3390/nu12030661](https://doi.org/10.3390/nu12030661).

138.	Melamed IR, Heffron M, Testori A, Lipe K. A pilot study of high-dose intravenous immunoglobulin 5% for autism: impact on autism spectrum and markers of neuroinflammation. *Autism Res* 2018; **11**(3)**:** 421-433. doi: [10.1002/aur.1906](https://doi.org/10.1002/aur.1906)

139.	Nadeem A, Ahmad SF, Al-Harbi NO, Al-Ayadhi LY, Sarawi W, Attia SM et al. Imbalance in pro-inflammatory and anti-inflammatory cytokines milieu in B cells of children with autism. *Mol Immunol* 2022; **141:** 297-304. doi: [10.1016/j.molimm.2021.12.009](https://doi.org/10.1016/j.molimm.2021.12.009)

140.	Pardo CA, Buckley A, Thurm A, Lee LC, Azhagiri A, Neville DM et al. A pilot open-label trial of minocycline in patients with autism and regressive features. *J Neurodev Disord* 2013; **5**(1)**:** 9. doi: [10.1186/1866-1955-5-9](https://doi.org/10.1186/1866-1955-5-9).

141.	Prosperi M, Guiducci L, Peroni DG, Narducci C, Gaggini M, Calderoni S et al. Inflammatory biomarkers are correlated with some forms of regressive autism spectrum disorder. *Brain Sci* 2019; **9**(12): 366. doi: [10.3390/brainsci9120366](https://doi.org/10.3390/brainsci9120366).

142.	Raghavan R, Zuckerman B, Hong X, Wang G, Ji Y, Paige D et al. Fetal and infancy growth pattern, cord and early childhood plasma leptin, and development of autism spectrum disorder in the Boston birth cohort. *Autism Res* 2018; **11**(10)**:** 1416-1431. doi: [10.1002/aur.2011](https://doi.org/10.1002/aur.2011)

143.	Ricci S, Businaro R, Ippoliti F, Lo Vasco VR, Massoni F, Onofri E et al. Altered cytokine and BDNF levels in autism spectrum disorder. *Neurotox Res* 2013; **24**(4)**:** 491-501. doi: [10.1007/s12640-013-9393-4](https://doi.org/10.1007/s12640-013-9393-4)

144.	Riordan NH, Hincapie ML, Morales I, Fernandez G, Allen N, Leu C et al. Allogeneic human umbilical cord mesenchymal stem cells for the treatment of autism spectrum disorder in children: safety profile and effect on cytokine levels. *Stem Cells Transl Med* 2019; **8**(10)**:** 1008-1016. doi: [10.1002/sctm.19-0010](https://doi.org/10.1002/sctm.19-0010)

145.	Russo AJ. Decreased mitogen inducible gene 6 (MIG-6) associated with symptom severity in children with autism. *Biomark Insights* 2014; **9:** 85-89. doi: [10.4137/BMI.S15218](https://doi.org/10.4137/BMI.S15218)

146.	Russo AJ, Krigsman A, Jepson B, Wakefield A. Decreased serum hepatocyte growth factor (HGF) in autistic children with severe gastrointestinal disease. *Biomark Insights* 2009; **4:** 181-190. doi: [10.4137/bmi.s3656](https://doi.org/10.4137/bmi.s3656)

147.	Sanctuary MR, Kain JN, Chen SY, Kalanetra K, Lemay DG, Rose DR et al. Pilot study of probiotic/colostrum supplementation on gut function in children with autism and gastrointestinal symptoms. *PLoS One* 2019; **14**(1)**:** e0210064. doi: [10.1371/journal.pone.0210064](https://doi.org/10.1371/journal.pone.0210064).

148.	Sasayama D, Kurahashi K, Oda K, Yasaki T, Yamada Y, Sugiyama N et al*.* Negative correlation between serum cytokine levels and cognitive abilities in children with autism spectrum disorder. *J Intell* 2017; **5**(2)**:** 19. doi: [10.3390/jintelligence5020019](https://doi.org/10.3390/jintelligence5020019).

149.	Schwarz E, Guest PC, Rahmoune H, Wang L, Levin Y, Ingudomnukul E et al*.* Sex-specific serum biomarker patterns in adults with Asperger's syndrome. *Mol Psychiatry* 2011; **16:** 1213-1220. doi: [10.1038/mp.2010.102](https://doi.org/10.1038/mp.2010.102)

150.	Schwarz E, Guest PC, Rahmoune H, Harris LW, Wang L, Leweke FM et al. Identification of a biological signature for schizophrenia in serum. *Mol Psychiatry* 2012; **17:** 494-502. doi: [10.1038/mp.2011.42](https://doi.org/10.1038/mp.2011.42)

151.	Shim S, Ha S, Choi J, Kwon HK, Cheon KA. Alterations in plasma cytokine levels in Korean children with autism spectrum disorder. *Yonsei Med J* 2024; **65**(2): 70-77. doi [10.3349/ymj.2023.0362](https://doi.org/10.3349/ymj.2023.0362)

152.	Than UTT, Nguyen LT, Nguyen PH, Nguyen XH, Trinh DP, Hoang DH et al. Inflammatory mediators drive neuroinflammation in autism spectrum disorder and cerebral palsy. Sci Rep 2023; **13**(1): 22587. doi: [10.1038/s41598-023-49902-8](https://doi.org/10.1038/s41598-023-49902-8)

153.	Werner MCF, Wirgenes KV, Shadrin A, Lunding SH, Rødevand L, Hjell G et al. Immune marker levels in severe mental disorders: associations with polygenic risk scores of related mental phenotypes and psoriasis. *Transl Psychiatry* 2022; **12**(1)**:** 38. doi: [10.1038/s41398-022-01811-6](https://doi.org/10.1038/s41398-022-01811-6).

154.	Yousefi J, Khakzad MR, Hojati M, Ebrahimi SA, Hosseinpour M, Akhondian J. Is serum TGF-beta 1 and TGF-beta 2 levels correlated to children with autism intensity? *Iran J Child Neurol* 2021; **15**(2)**:** 47-57. doi: [10.22037/ijcn.v15i1.21826](https://doi.org/10.22037/ijcn.v15i1.21826)

155.	Zimmerman AW, Singh K, Connors SL, Liu H, Panjwani AA, Lee LC et al. Randomized controlled trial of sulforaphane and metabolite discovery in children with autism spectrum disorder. *Mol Autism* 2021; **12**(1)**:** 38. doi: [10.1186/s13229-021-00447-5](https://doi.org/10.1186/s13229-021-00447-5).

156.	Tsilioni I, Dodman N, Petra AI, Taliou A, Francis K, Moon-Fanelli A et al. Elevated serum neurotensin and CRH levels in children with autistic spectrum disorders and tail-chasing Bull Terriers with a phenotype similar to autism. *Transl Psychiatry* 2014; **4**(10)**:** e466. doi: [10.1038/tp.2014.106](https://doi.org/10.1038/tp.2014.106).

157.	Zimmerman AW, Jyonouchi H, Comi AM, Connors SL, Milstien S, Varsou A et al. Cerebrospinal fluid and serum markers of inflammation in autism. *Pediatr Neurol* 2005; **33**(3)**:** 195-201. doi: [10.1016/j.pediatrneurol.2005.03.014](https://doi.org/10.1016/j.pediatrneurol.2005.03.014)

158.	Alhakbany M, Al-Ayadhi L, El-Ansary A. CTRP3 as a novel biomarker in the plasma of Saudi children with autism. *PeerJ* 2022; **10:** e12630. doi: [10.7717/peerj.12630](https://doi.org/10.7717/peerj.12630).

159.	Al-Ayadhi LY, Mostafa GA. Increased serum osteopontin levels in autistic children: Relation to the disease severity. *Brain Behav Immun* 2011; **25:** 1393-1398. doi: [10.1016/j.bbi.2011.04.006](https://doi.org/10.1016/j.bbi.2011.04.006)

160.	Emanuele E, Boso M, Brondino N, Pietra S, Barale F, Ucelli di Nemi S et al. Increased serum levels of high mobility group box 1 protein in patients with autistic disorder. *Prog Neuropsychopharmacol Biol Psychiatry* 2010; **34**(4)**:** 681-683. doi: [10.1016/j.pnpbp.2010.03.020](https://doi.org/10.1016/j.pnpbp.2010.03.020)

161.	Makris G, Chouliaras G, Apostolakou F, Papageorgiou C, Chrousos GP, Papassotiriou I et al. Increased serum concentrations of high mobility group box 1 (HMGB1) protein in children with autism spectrum disorder. *Children (Basel)* 2021; **8**(6): 478. doi: [10.3390/children8060478](https://doi.org/10.3390/children8060478).

162.	Nadeem A, Ahmad SF, Attia SM, Al-Ayadhi LY, Al-Harbi NO, Bakheet SA. Dysregulation in IL-6 receptors is associated with upregulated IL-17A related signaling in CD4+T cells of children with autism. *Prog Neuro-Psychopharmacol Biol Psychiatry* 2020; **97:** 7. doi: [10.1016/j.pnpbp.2019.109783](https://doi.org/10.1016/j.pnpbp.2019.109783).

163.	Raeisy H, Bayati P, Noorbakhsh F, Hakim Shooshtari M, Eftekhar Ardebili M, Shekarabi M et al. C1q/TNF-related protein-1: potential biomarker for early diagnosis of autism spectrum disorder. *Int J Immunopathol Pharmacol* 2022; **36:** 3946320221079471. doi: [10.1177/03946320221079471](https://doi.org/10.1177/03946320221079471).

164.	Rose D, Ashwood P. Rapid communication: Plasma interleukin-35 in children with autism. *Brain Sci* 2019; **9**(7): 152. doi: [10.3390/brainsci9070152](https://doi.org/10.3390/brainsci9070152).

165.	Russo AJ. Increased epidermal growth factor receptor (EGFR) associated with hepatocyte growth factor (HGF) and symptom severity in children with autism spectrum disorders (ASDs). *J Cent Nerv Syst Dis* 2014; **6**: 79-83. doi: [10.4137/JCNSD.S13767](https://doi.org/10.4137/jcnsd.s13767)

166.	Tsilioni I, Pantazopoulos H, Conti P, Leeman SE, Theoharides TC. IL-38 inhibits microglial inflammatory mediators and is decreased in amygdala of children with autism spectrum disorder. *Proc Natl Acad Sci U S A* 2020; **117**(28)**:** 16475-16480. doi: [10.1073/pnas.2004666117](https://doi.org/10.1073/pnas.2004666117)
